# *De Novo* Characterization of the Spleen Transcriptome of the Large Yellow Croaker (*Pseudosciaena crocea*) and Analysis of the Immune Relevant Genes and Pathways Involved in the Antiviral Response

**DOI:** 10.1371/journal.pone.0097471

**Published:** 2014-05-12

**Authors:** Yinnan Mu, Mingyu Li, Feng Ding, Yang Ding, Jingqun Ao, Songnian Hu, Xinhua Chen

**Affiliations:** 1 Key Laboratory of Marine Biogenetics and Resources, Third Institute of Oceanography, State Oceanic Administration, Xiamen, China; 2 Collaborative Innovation Center of Deep Sea Biology, Third Institute of Oceanography, State Oceanic Administration, Xiamen, China; 3 The CAS Key Laboratory of Genome Sciences and Information, Beijing Institute of Genomics, Chinese Academy of Sciences, Beijing, China; 4 Biological and Environmental Sciences and Engineering Division, King Abdullah University of Science and Technology, Thuwal, Saudi Arabia; University of Hong Kong, China

## Abstract

The large yellow croaker (*Pseudosciaena crocea*) is an economically important marine fish in China. To understand the molecular basis for antiviral defense in this species, we used Illumia paired-end sequencing to characterize the spleen transcriptome of polyriboinosinic:polyribocytidylic acid [poly(I:C)]-induced large yellow croakers. The library produced 56,355,728 reads and assembled into 108,237 contigs. As a result, 15,192 unigenes were found from this transcriptome. Gene ontology analysis showed that 4,759 genes were involved in three major functional categories: biological process, cellular component, and molecular function. We further ascertained that numerous consensus sequences were homologous to known immune-relevant genes. Kyoto Encyclopedia of Genes and Genomes orthology mapping annotated 5,389 unigenes and identified numerous immune-relevant pathways. These immune-relevant genes and pathways revealed major antiviral immunity effectors, including but not limited to: pattern recognition receptors, adaptors and signal transducers, the interferons and interferon-stimulated genes, inflammatory cytokines and receptors, complement components, and B-cell and T-cell antigen activation molecules. Moreover, the partial genes of Toll-like receptor signaling pathway, RIG-I-like receptors signaling pathway, Janus kinase-Signal Transducer and Activator of Transcription (JAK-STAT) signaling pathway, and T-cell receptor (TCR) signaling pathway were found to be changed after poly(I:C) induction by real-time polymerase chain reaction (PCR) analysis, suggesting that these signaling pathways may be regulated by poly(I:C), a viral mimic. Overall, the antivirus-related genes and signaling pathways that were identified in response to poly(I:C) challenge provide valuable leads for further investigation of the antiviral defense mechanism in the large yellow croaker.

## Introduction

The large yellow croaker (*Pseudosciaena crocea*) is an economically important marine fish in China. With the rapid development of the large yellow croaker culture industry in recent years, there have been increasingly severe outbreaks of infectious disease caused by viruses, bacteria and *Cryptocaryon irritans* [1], [2], resulting in great economic losses. However, information on the mechanisms underlying the immune response to these pathogens, especially viruses, is currently limited in this fish species. This creates a significant dilemma in the development of effective strategies to prevent these diseases.

During the infection of host cells, RNA viruses produce double-stranded RNA (dsRNA) in the process of RNA-dependent RNA synthesis [3]. Some DNA viruses also generate dsRNA during their life cycle [3], [4]. Thus, these replicative intermediate dsRNA, along with genomic dsRNA from dsRNA viruses, can function as a pathogen-associated molecular pattern signaling viral infection [3], [4], [5]. Polyriboinosinic:polyribocytidylic acid [poly(I:C)], which is the synthetic analog of viral dsRNA, can trigger the innate immune system to secrete type I interferons (IFNs) in vertebrates, including fish [6]. Poly(I:C) has been used as a viral mimic for the study of the immune response to viruses, and it has similar but not all of the stimulating effects of viral dsRNA [7], [8], [9]. As in higher vertebrates, the IFN system is considered to be one of the first lines of defense against viruses in fish [10], although the exact antiviral mechanism remains to be elucidated.

Recently, the advent of RNA sequencing (RNA-seq) technology and progresses in bioinformatics, especially in the development of *de novo* assembly tools, have provided a powerful platform for the rapid and nearly complete characterization of transcriptomic events in various species [11], [12]. Using RNA-seq technology, several studies have reported the transcriptome profile of model and aquaculture fish after viral or bacterial challenge, thereby revealing insight into immune-relevant genes and pathways. Examples of these species are the zebrafish (*Danio rerio*) [13], sea bass (*Lateolabrax japonicas*) [14], orange-spotted grouper (*Epinephelus coioides*) [15], common carp (*Cyprinus carpio*) [16], and turbot (*Scophthalmus maximus*) [17]. Previously, we reported a transcriptome profile from the large yellow croaker following bacterial infection (*Aeromonas hydrophila*) [18]. However, the molecular basis and mechanism for antiviral defense are still poorly understood in this fish species. To further understand the molecular basis for antiviral defense and explore the antiviral genes and pathways in this fish, we used the Solexa/Illumina sequencing technology to conduct a transcriptome profiling analysis of large yellow croakers that were challenged with poly(I:C). We ultimately obtained a transcriptome database containing 15,192 unigenes. Unigenes that were found to be involved in the immune response and immune-relevant pathways were further analyzed. Moreover, real-time PCR was used to analyze the expression changes of partial immune-relevant genes after poly(I:C) induction. These data provide valuable leads for further investigation of the antiviral defense mechanism in the large yellow croaker.

## Results and Discussion

### Sequence analysis of the transcriptome in poly(I:C)-induced fish

**Figure 1:**
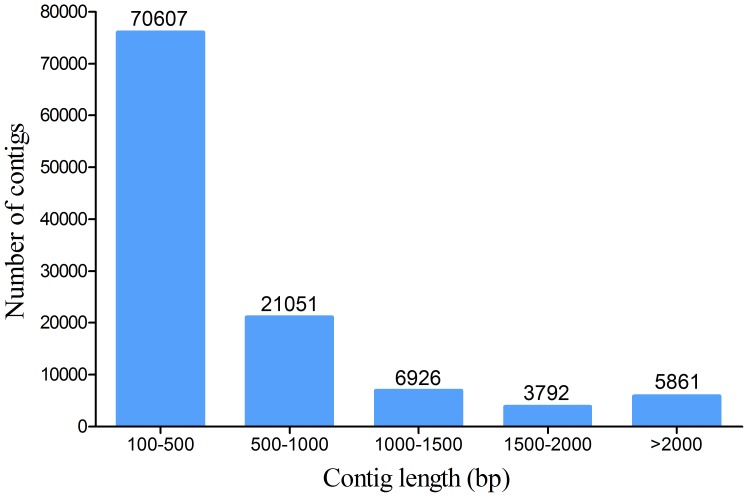
Length statistics of contigs obtained from the large yellow croaker transcriptome library. The length distribution of the transcriptome library is shown. Sequences with lengths of 500-1000 bp were most abundant, making up 65% of the contigs.

This library produced 56,355,728 reads from high-throughput paired-end sequencing. The high-quality reads were assembled into 108,237 contigs using the short oligonucleotide analysis package (SOAP) *de novo* software, with a maximum contig length of 24,820 bp. The length statistics of all contigs are presented in **Fig. 1**. Using the zebrafish RefSeq mRNA database as the reference, tBLASTX similarity searches revealed that 22,120 of the 108,237 contigs (20%) shared homology with zebrafish genes when a cutoff E-value of 1e^−05^ was used. If query sequences hit the same zebrafish genes, they were clustered. Ultimately, 8,849 unigenes were annotated from 22,120 contigs. The remaining 86,117 contigs were further searched against the non-redundant database, and 6,343 genes were annotated after clustering. Overall, 15,192 unigenes were annotated from the spleen transcriptome of poly(I:C)-induced fish (**Table S1**). In addition, 78,793 contigs failed to match proteins in the non-redundant database and, therefore, represented potentially novel genes.

### Categories of the most abundantly expressed genes in the transcriptome profile

We estimated gene expression levels by quantifying the abundance of reads belonging to the same gene from the transcriptome. Among the 20 most highly expressed transcripts (**Table 1**), many genes coded for the ribosomal proteins, such as ribosomal protein (RPS) 15a, RPS25, RPS27a, RPL6, RPL10E, RPL13a, RPL23a, RPL26, RPL28, RPL34, RPL35, RPL36, and RPL36a. The ribosomal proteins are integral components of the basal cellular machinery that is involved in ribosome biogenesis, which is required in every living cell [19]. Other than functioning in protein biosynthesis within the ribosome, some ribosomal proteins are involved in other cellular processes. RPS25 is involved in the regulation of the Mouse double minute 2 homolog (MDM2)-p53 pathway in coordinating the cellular response to stress [20]. Phosphorylation of RPL13a is essential for the translational repression of inflammatory genes by the IFN-γ-activated inhibitor of translation complex [21]. Moreover, the top 20 genes included those that are involved in protein degradation (ubiquitin A-52 residue ribosomal protein fusion product 1) [22], oxygen transport (hemoglobin subunit β-2) [23], DNA replication (origin recognition complex subunit 2) [24], and senescence (senescence-associated protein).

**Table 1:** Top 20 most abundant unigenes in the transcriptome of the large yellow croaker.

No.	Gene Name	Reads Number	Description	Blast E-Value
1		229963	hypothetical protein	2.00E-08
2	SAP	135992	senescence-associated protein	2.00E-10
3	SAP	80274	putative senescence-associated protein	5.00E-28
4		79645	hypothetical protein	2.00E-14
5	HBB2	30036	hemoglobin subunit beta-2	1.00E-38
6	RPL35	21572	60S ribosomal protein L35	1.00E-06
7	RPL36a	19528	60S ribosomal protein L36a	3.00E-16
8	UBA52	17051	ubiquitin A-52 residue ribosomal protein fusion product 1	7.00E-41
9	RPL36	17022	60S ribosomal protein L36	3.00E-16
10	ORC2L,	16568	origin recognition complex subunit 2-like	2.00E-63
11	RPS25	14815	40S ribosomal protein S25	3.00E-30
12	RPS15a	14414	ribosomal protein S15a	4.00E-37
13	RPS27a	13087	ribosomal protein S27a	2.00E-33
14	RPL23a	12850	ribosomal protein L23a	2.00E-23
15	RPL13a	12581	ribosomal protein L13a	3.00E-41
16	RPL10E	11540	60S acidic ribosomal protein P0 (L10E)	2.00E-62
17	RPL6	10863	ribosomal protein L6	4.00E-48
18	RPL28	10037	ribosomal protein L28-like	8.00E-16
19	RPL34	9821	60S ribosomal protein L34	1.00E-67
20	RPL26	9852	ribosomal protein L26	7.00E-34

### Annotation of unique sequences, based on Gene Ontology (GO) and Kyoto Encyclopedia of Genes and Genomes (KEGG)

**Figure 2:**
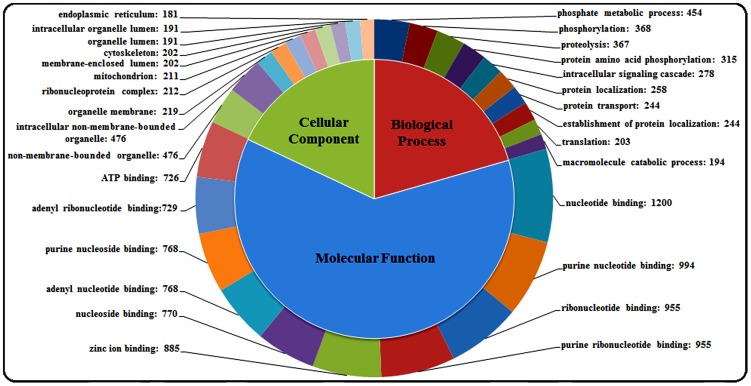
Annotation of large yellow croaker unique sequences, according to GO. GO terms were derived based on the similarity search with web-based database, DAVID. The top ten GO terms in the cellular component, molecular function, and biological process groups are displayed.

GO[25] analysis of these genes was performed using the web-based Database Annotation, Visualization, and Integrated Discovery (DAVID) to produce an overview of the gene expression profile of the transcriptome library [26], [27] (**Table S2**). According to their GO terms, 4,759 of the total 15,192 genes were classified into three major functional categories: “biological process”, “molecular function”, and “cellular component”, which were represented by 12,104, 14,168, and 5,696 genes, respectively. Because some genes were classified into more than one subcategory within each of the three major categories, the sum of genes in the subcategories could exceed 100%. The top ten GO terms of each functional category are shown in **Fig. 2**. Phosphate metabolic process (15.46%) and phosphorylation (12.53%) were the main subcategories of the biological process group. The largest subcategory found in the cellular component group was non-membrane-bounded organelle (32.99%). In the molecular function category, nucleotide binding (36.34%) was the most abundant GO term.

**Figure 3:**
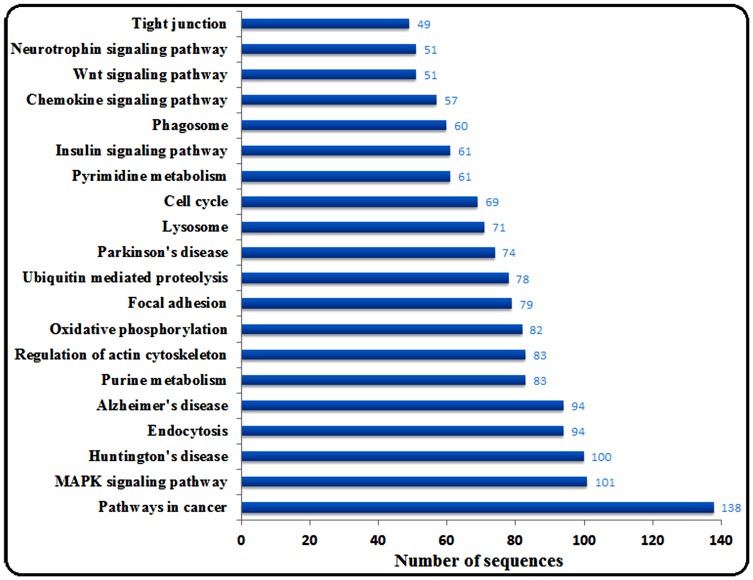
KEGG orthology classification of unigenes. KEGG gene function was performed using the web-based database, DAVID. The numbers of sequences assigned to the twenty top KO categories were calculated.

To identify the biological pathways from this transcriptome, we mapped 15,192 genes to canonical signaling pathways that are found in the KEGG. A number of assembled unigenes (5,389 total, 35.47%) were assigned with KEGG orthology (KO) identifiers, and 251 pathways were associated (**Table S3**). The top 20 pathways are summarized in **Fig. 3**. The results of GO and KEGG annotations provide important information for further investigation of the antiviral response and processes in the large yellow croaker.

### Annotation of immune-relevant genes and pathways

To gain more insight into the molecular basis of the immune system in the large yellow croaker, the immune-relevant genes were analyzed from 15,192 unigenes (**Table S4**). The main immune-relevant genes that were homologous to other species are described in **Table 2**. These include the most important elements of innate and adaptive immunity, such as the pattern recognition receptors (TLR1-3, TLR5, TLR9, TLR22, MDA5, NOD1-3, NLRC3, NLRC5, and NLRX1), signal transducers (MyD88, TRAF3, IRAK4, TRAF6, TAK1, TBK1, LCK, and SPL76), interleukins (IL-1β, IL-2, IL-6, IL-8, IL-10, IL-12p35/p40, IL-12b, and IL-17d), the classical complement pathway (C1q, C3b, C6, Cfb, and Cfb/C2-b), lectin pathway(MASP2, MBL2, and MBL2la), growth factor-β family (TGF-β1 and TGF-β2), transcriptional factors (NF-κB, AP-1, p38, JNK, NFAT, and IRF1-10), antigen-presenting and processing molecules (MHCIα, β2m, and MHCIIβ), leukocyte differentiation antigens (CD3, CD4, CD8, CD22, CD45, and CD81), and other molecules that are involved in the immune response (Caspase family members, chemokines and chemokine receptors, lectins, Galectin, erlectin, and Malectin, **Table S4**). As expected, two type I IFN molecules (IFN1/3) were found in the transcriptome. This was consistent with the previous studies showing that the expression of fish type I IFNs is increased by poly(I:C) [28], [29], [30], [31]. KEGG analysis revealed that unigenes were enriched in various known immune-relevant signaling pathways. Most of the immune-relevant pathways are listed in **Table 3**. Importantly, these immune-relevant genes and signaling pathways provide the basis for further identification of the biological response mechanism to viral infections in the large yellow croaker.

**Table 2:** Immune-relevant genes found in the poly(I:C)-induced transcriptome.

Gene name	Description	Gene name	Description
	Pattern recognition receptors		Interferons and interferon stimulated genes
TLR1	toll-like receptor 1	IFN1	interferon 1
TLR2	toll-like receptor 2	IFN3	TPA: type I interferon
TLR3	toll-like receptor 3	IFI30	interferon gamma inducible protein 30
TLR5b	toll-like receptor 5b	IFI44l	interferon-induced protein 44-like
TLR9	toll-like receptor 9	ISG56	interferon-inducible protein 56
TLR22	toll-like receptor 22	Gig1	interferon-inducible protein Gig1
NOD1	Nod 1 protein	Mxa	Interferon-induced GTP-binding protein MxA
NOD2	Nod 2 protein	Mxb	Interferon-induced GTP-binding protein MxB
NOD3	Nod 3 protein	Mxe	Interferon-induced GTP-binding protein MxE
NLRC3	NLR family, pyrin domain containing 3	ISG20	Interferon-stimulated exonuclease gene 20-like 2
NLRC5	NLRC5 receptor	IFRD1	interferon-related developmental regulator 1
NLRX1	NLRX1 receptor	IFRD2	interferon-related developmental regulator 2
MDA5	Melanoma differentiation associated protein 5	viperin	Radical S-adenosyl methionine domain containing protein 2
	Adapters, effectors and signal transducers	ADAR	adenosine deaminase, RNA-specific
MyD88	myeloid differentiation primary response protein 88	eif2ak2	eukaryotic translation initiation factor 2 alpha kinase 2
TRAF3	TNF receptor-associated factor 3		Interferon regulatory factors
TRAF6	TNF receptor-associated factor 6	IRF1	interferon regulatory factor 1
IRAK4	interleukin-1 receptor-associated kinase 4	IRF2	interferon regulatory factor 2
TAB1	TAK1-Binding Protein 1	IRF2a	interferon regulatory factor 2a
TAB2	TAK1-Binding Protein 2	IRF2b	interferon regulatory factor 2b
TAK1	TGF-beta activated kinase 1	IRF3	interferon regulatory factor 3
TBK1	TANK-binding kinase 1	IRF4	interferon regulatory factor 4
FADD	FAS-associated death domain protein	IRF5	interferon regulatory factor 5
TICAM1	toll-like receptor adaptor molecule 1	IRF6	interferon regulatory factor 6
TANK	TRAF-interacting protein	IRF7	interferon regulatory factor 7
AKT	RAC serine/threonine-protein kinase	IRF8	interferon regulatory factor 8
TOLLIP	toll-interacting protein	IRF9	interferon regulatory factor 9
TRADD	Tumor necrosis factor receptor type 1-associated DEATH domain protein	IRF10	interferon regulatory factor 10
Rac1	RAS-related C3 botulinum substrate 1		JAK-STAT signaling pathway
NAP1	nucleosome assembly protein 1	Jak1	Tyrosine-protein kinase Jak1
RIP2	receptor-interacting serine-threonine kinase 2	Jak2a	Janus kinase 2a
CRAD9	caspase recruitment domain protein 9	Jak2	Janus kinase 2
PIK3C3	catalytic phosphatidylinositol 3-kinase 3	Jak3	JAK3 tyrosine kinase
NFκB2	nuclear factor of kappa light polypeptide gene enhancer in B-cells 2, p49/p100	Stat1a	signal transducer and activation of transcription 1a
IKKε	Inhibitor-κB kinase ε	Stat1b	signal transducer and activator of transcription 1b
AP-1	AP-1	Stat3	signal transducer and activation of transcription 3
P38	p38 MAP kinase	Stat4	signal transducer and activator of transcription 4
JNK	c-Jun NH(2)-terminal kinase	Stat5.1	signal transducer and activator of transcription 5.1
NFAT	nuclear factor of activated T-cells	Stat5.2	signal transducer and activator of transcription 5.2
MAPK1	mitogen-activated protein kinase 1	Stat6	signal transducer and activator of transcription 6
MAPK3	mitogen-activated protein kinase 3		Complement System
MAPK6	mitogen-activated protein kinase 6	C1qBP	complement component 1, q subcomponent binding protein
MAPK7	mitogen-activated protein kinase 7	C3b	complement component c3b
MAPK8	mitogen-activated protein kinase 8	C6	complement component 6
MAPK10	mitogen-activated protein kinase 10	CFB	complement factor B
MAPK11	mitogen-activated protein kinase 11	CFB/C2B	complement factor B/C2-B
MAPK12	mitogen-activated protein kinase 12	MASP2	mannan-binding lectin serine peptidase 2
	Inflammatory cytokines and receptors	MBL2	mannose-binding lectin (protein C) 2
IL-1β	interleukin-1, beta		T-cell and B-cell antigen activation
IL-2	interleukin-2	IgH	immunoglobulin heavy chain IgH
IL-6	interleukin-6	IgL	immunoglobulin light chain type 1
IL-8	interleukin-8	IgD	immunoglobulin D
IL-10	interleukin-10	MHCIα	MHC class IA antigen
IL-12p35	interleukin-12 p35 subunit	MHCIIβ	MHC class II beta antigen
IL-12p40	interleukin-12 p40 chain	β2m	β2 microglobulin
IL-12b	interleukin-12b	bcl2	bcl2
IL-17d	interleukin-17d	bcl6	B-cell lymphoma 6 protein
IL-1R1	interleukin-1 receptor-like 1 ligand	bcl7b	B-cell CLL/lymphoma 7b
IL-2Rβ	interleukin-2 receptor, beta	bcl11a	B-cell CLL/lymphoma 11A
IL-2Rγa	interleukin-2 receptor, gamma a	Blnk	B-cell linker
IL-6R	interleukin-6 receptor	TCRa	T-cell receptor alpha
IL-7R	interleukin-7 receptor	TCRb	T-cell receptor beta chain
IL-10Rβ	interleukin-10 receptor beta chain	TRBC	T cell receptor beta chain constant region
IL12Rβ2	interleukin-12 receptor, beta 2a, like	TVA1	T-cell receptor alpha chain V region HPB-MLT precursor
IL-17R	interleukin-17 receptor	TCRb	T-cell receptor beta chain precursor
IL-17RA	interleukin-17 receptor A precursor	TCRV-a	T cell receptor V alpha chain
IL-21R	interleukin-21 receptor	TCRV-a6	T-cell receptor V-alpha6 chain precursor
CC	CC chemokine	CD3ε	CD3 epsilon
CC3	C-C motif chemokine 3 precursor	CD3-γ/δ	CD3 gamma/delta
CC20	chemokine (C-C motif) ligand 20-like	CD4-2	CD4-2 protein
CXCl14	chemokine (C-X-C motif) ligand 14	CD4-4	CD4-2 protein
CXCl12a	chemokine (C-X-C motif) ligand 12a	CD8	CD8 beta
CXCl12b	chemokine (C-X-C motif) ligand 12b	CD22	CD22 molecule
Cmklr1	chemokine-like receptor 1	CD45	CD45
CXCr7b	chemokine (C-X-C motif) receptor 7b	CD81	CD81 antigen
CXCr3.1	chemokine (C-X-C motif) receptor 3.1	LCK	lymphocyte cell-specific protein tyrosine kinase
CXCr3.2	chemokine (C-X-C motif) receptor 3.2	FYN	tyrosine-protein kinase Fyn
CXCr4a	chemokine (C-X-C motif) receptor 4a	SLP76	lymphocyte cytosolic protein 2
CXCr4b	chemokine (C-X-C motif), receptor 4b	NCK	NCK adaptor protein
CCr6a	chemokine (C-C motif) receptor 6a	PAK	p21-activated kinase 1
CCr7	chemokine (C-C motif) receptor 7	VAV	guanine nucleotide exchange factor VAV
CCr12.3	C-C chemokine receptor family-like	ITK	IL2-inducible T-cell kinase
TNFb	TNF superfamily, member 2	CaN	serine/threonine-protein phosphatase 2B catalytic subunit
TNF-α	tumor necrosis factor alpha	Raf	RAF proto-oncogene serine/threonine-protein kinase
TGF-β1	transforming growth factor, beta 1	Ras	GTPaseHRas
TGF-β2	transforming growth factor, beta 2		

**Table 3:** T**able 3.** Immune-relevant pathways annotated in the spleen transcriptome.

Pathway name	KO identifier	Mapped genes	Known genes
Toll-like receptor signaling pathway	ko04620	37	76
NOD-like receptor signaling pathway	ko04621	25	51
RIG-I-like receptor signaling pathway	ko04622	23	53
Natural killer cell-mediated cytotoxicity	ko04650	31	79
Chemokine signaling pathway	ko04062	57	139
Cytokine-cytokine receptor interaction	ko04060	42	224
Leukocyte transendothelial migration	ko04670	45	72
Jak-STAT signaling pathway	ko04630	35	121
Complement and coagulation cascades	ko04610	20	69
Apoptosis	ko04210	39	64
Antigen processing and presentation	ko04612	22	41
Fc epsilon RI signaling pathway	ko04664	25	44
Fc gamma R-mediated phagocytosis	ko04666	39	55
T-cell receptor signaling pathway	ko04660	46	81
B-cell receptor signaling pathway	ko04662	33	52

### Toll-like receptor (TLR) signaling pathway

**Figure 4:**
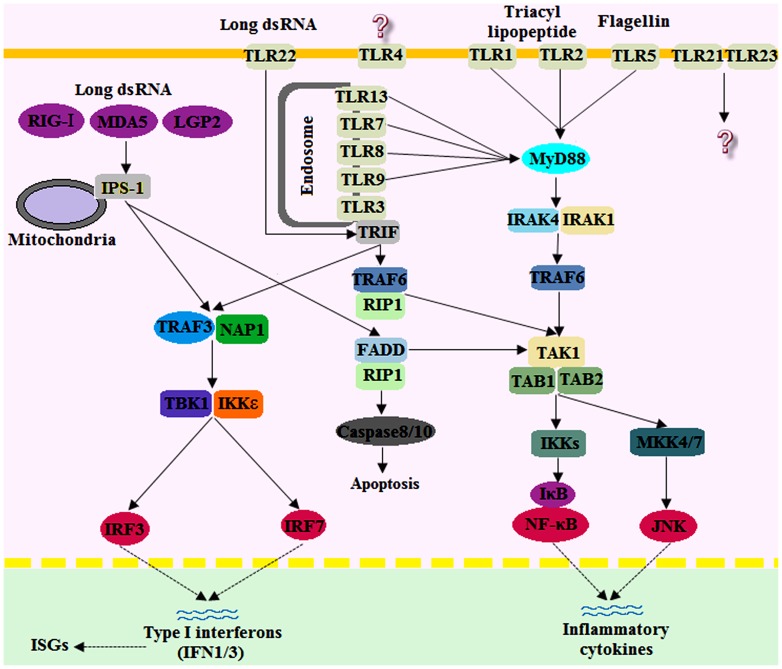
Putative Toll-like receptor and RIG-I-like receptor signaling pathway. The putative Toll-like receptor and RIG-I-like receptor signaling pathways of the large yellow croaker were constructed based on the knowledge of TLR signaling in mammalian species. However, most interactions need to be confirmed experimentally.

**Figure 5:**
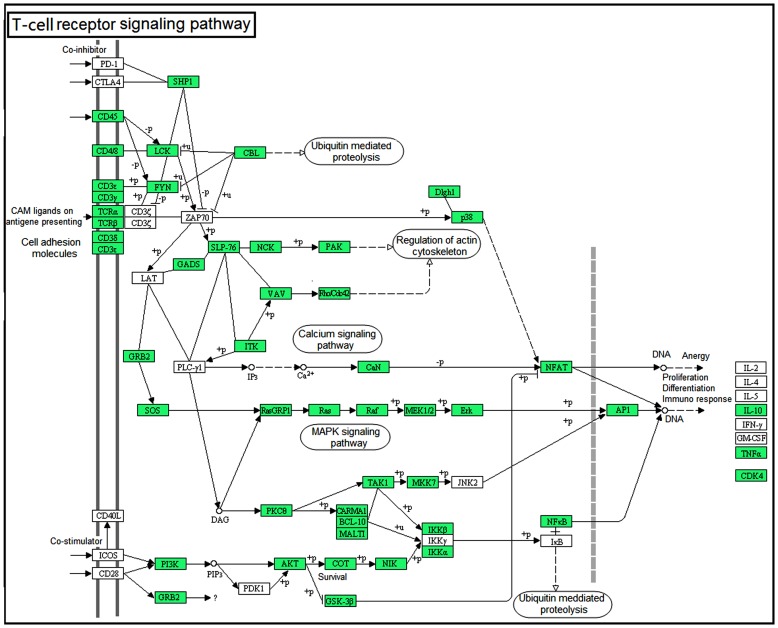
Real-time PCR analysis of selected genes. Total RNA was extracted from the spleens of large yellow croakers sampled at 0, 12 and 24(I:C) induction. Real-time PCR was used to validate gene expression changes in the pattern recognition receptors (A), signal transducers (B), interferons and interleukin (C), interferon-stimulated genes (D), JAK-STAT pathway (F), and T-cell receptor (TCR) signaling pathway. Increases and decreases in the relative levels of transcripts with respect to the control β-actin gene are shown.^*^ *P*<0.05, ^**^ *P*<0.01.

TLRs are the most important class of pattern recognition receptors (PRRs), and they recognize a broad range of pathogens, including viruses, bacteria, and fungi. TLRs recognize their ligands through interactions with the LRRs and trigger the activation of intracellular signaling through a cytoplasmic MyD88-dependent pathway or a MyD88-independent pathway [32]. All TLRs depend, at least in part, on the MyD88 adaptor for full signal transduction activity, with the exception of TLR3. TLR3 exclusively uses the MyD88-independent pathway, where it can signal by recruiting the adaptor protein, TIR-domain-containing adapter-inducing interferon-β (TRIF). TRIF associates with TANK-binding kinase 1 (TBK1), which initiates the phosphorylation and activation of IFN regulatory factor 3 (IRF3), thus leading to the expression of type I IFNs [33], [34]. In the present study, a series of TLRs, corresponding adaptor proteins, transcription factors, and downstream effectors were identified in the spleen transcriptome of the large yellow croakers. The identified TLRs included those that are normally found in mammals (TLR1-3, TLR5, and TLR9), along with the fish-specific TLR22. Important adaptor proteins that are identified in mammals, such as MyD88, IRAK4, TRAF6, and TAK1 in the MyD88-dependent pathway and TRAF3, TBK1, and IRF3 in the MyD88-independent pathway, were also found in this transcriptome. Downstream effectors, such as type I IFNs (IFN1/3), TNF-α, IL-1β, IL-6, IL-8, and IL-12, were successfully identified (**Table 2**). These results suggest that TLRs and the TLR signaling pathway are conserved from fish to mammals. A putative draft of the TLR signaling pathway was constructed based on the knowledge of pathways that are known in mammals and fish (**Fig. 4**). TLR3 and TLR22 serve as surveillants for infections with the dsRNA virus, and both can activate signaling pathways for antiviral protection in fish. In the endoplastic reticulum, TLR3 recognizes relatively short-sized dsRNA, whereas TLR22 recognizes long-sized dsRNA on the cell surface. Both TLRs link to the MyD88-independent pathway via TICAM-1 or TRIF in fish cells [35]. Real-time PCR analysis revealed that TLR3, TLR22, signal transducers (TRAF3 and TBK1), transcriptional factor IRF3/7, and downstream molecules (IFN1/3 and IL-12) were up-regulated at different levels upon poly(I:C) induction (**Fig. 5A, B and C**). These results provide evidence that the TLR3- and TLR22-mediated pathways may play an important role in the antiviral immunity of the large yellow croaker.

### RIG-I-like receptor signaling pathway

The second group of PRRs involved in the recognition of viral nucleic acids are the cytoplasmic RNA helicases termed the RIG-I-like receptors (RLRs), including retinoic acid-inducible gene 1 (RIG-I), MDA5 and laboratory of genetics and physiology 2 (LGP2) [36]. RIG-I and MDA5 detect dsRNA intermediates and interact with the adaptor molecule, IPS-1 [37], which is localized on the mitochondria. IPS-1 recruits TRAF3 and activates both TBK1 and IKKε to induce the phosphorylation and nuclear translocation of IRF3 and IRF7, as well as the production of IFNs and IFN-stimulated genes (ISGs) [38], [39]. The only RLR that was identified in the poly(I:C)-induced transcriptome was MDA5 (**Table 2** and **Fig. 4**). MDA5 and LPG2 seem to be common to all teleost genomes, whereas teleost RIG-I has been identified only for cyprinids and salmonids. This suggests that RIG-I is either lost from particular fish genomes or has diverged to a level that is no longer recognizable [40], [41]. Whether RIG-I exists in the large yellow croaker genome is unknown. However, important intermediates of the RLR signaling pathway, such as TRAF3, TBK1, and IKKε, were identified (**Table 2**). Real-time PCR analysis also revealed that MDA5, TRAF3, and TBK1 expression was increased by poly(I:C) (**Fig. 5A and B**), suggesting that the RLR signaling pathway may be involved in the poly(I:C)-induced response. These data provide useful information for understanding the role of RLRs in antiviral immunity in the large yellow croaker.

### JAK-STAT signaling pathway

The JAK-STAT pathway is initiated in response to cytokines, such as IFNs and ILs, and it has important roles in the regulation of immune responses [42], [43]. Upon the binding of type I IFNs to their cell surface receptors, Jak1 and Tyrosine kinase 2 are activated. This leads to the phosphorylation of the cytoplasmic region of the receptors to allow for the recruitment of Stat1 and Stat2, which results in the formation of the IFN-stimulated gene factor3 (ISGF3). It then translocates into the nucleus and induces the expression of ISGs [44]. In our study, three Jaks (Jak1, Jak2a, and Jak2b), seven Stats (Stat1a, Stat1b, Stat3, Stat4, Stat5.1, Stat5.2, and Stat6), and several ISGs (MxA, MxB, MxE, PKR, ADAR, viperin, and IRFs, **Table 2**) were found. Real-time PCR analysis revealed that the expression of Stats (Stat1a, Stat3, and Stat6) and ISGs (MxA, PRK, and viperin) were all increased within 24 hours (**Fig. 5D and E**). Therefore, the identified changes in gene expression suggest that the JAK-STAT pathway may be activated by poly(I:C) stimulation. Stat1 and Stat2 play known roles in the type I IFN-induced antiviral response, whereas Stat3 can negatively regulate the response [45]. The coordinated up-regulation of Stat3 by poly(I:C) suggests that the JAK-STAT pathway may be negatively regulated to maintain the immune balance in fish. Further studies are needed to elucidate the regulatory mechanisms of the JAK-STAT pathway in the antiviral response in fish.

### T-cell receptor (TCR) and B-cell receptor (BCR) signaling pathways

**Figure 6:**
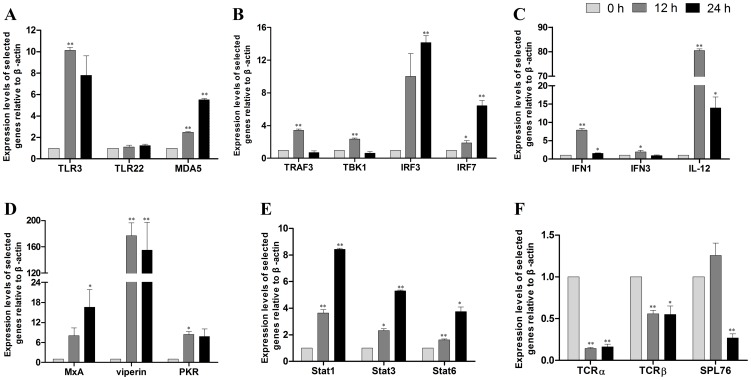
Map of the T-cell receptor signaling pathway, as generated by KEGG. Genes that were identified from the transcriptome of the large yellow croaker spleen are shown in green. White denotes genes that were not identified in the transcriptome analysis.

The adaptive immunity, which relies on the generation of random and highly diverse repertoires of T- and B-lymphocyte receptors that are encoded by recombinant activation genes (RAGs), contributes to a more specific and efficient response against pathogen infections [46]. The TCR is a complex of integral membrane proteins, which consist of the highly variable α and β chains that are expressed with the invariant CD3 chain molecules [47]. Activation of the TCR pathway initiates positive and negative cascades that ultimately result in cellular proliferation, differentiation, cytokine production, and/or activation-induced cell death [48]. Upon engagement of the TCR by antigen presented on MHC molecules, LCK is activated, and zeta-chain-associated protein kinase 70 activation is promoted [49]. Afterwards, LAT and SLP-76 are phosphorylated, thus resulting in the activation of the Ras pathway, calcium mobilization, and cytoskeletal reorganization [49]. The present study successfully identified a large number of relevant components of the TCR signaling pathway, such as several hallmarks (TCRα/β, CD3ε/γ/δ, CD4, CD8α, and CD8β), co-stimulatory factor (CD40 and CD83) and signaling transducers (LCK, SPL76, CaN, Ras, and Raf)**(** **Fig. 6** **)**. Real-time PCR analysis showed that some TCR signaling pathway members, including TCRα/β chain and SPL76, were down-regulated significantly (**Fig. 5F**), implying that the TCR signaling pathway may be suppressed in the early period (12 h) following poly(I:C) induction.

Activation of the BCR signaling pathway by binding of the antigen to the BCR complex causes B-cell growth and proliferation, as well as the creation of an amplified clone of effector cells that secretes the antigen-specific immunoglobulin [50]. Signaling through the BCR requires a highly coordinated set of interactions involving several transmembrane and cytosolic proteins, such as Ig heavy chain, Ig light chain, CD22, CD81, LYN, BTK, and BLNK, which were all identified in this transcriptome. These results show that the basic components and signaling pathways necessary for adaptive immunity existed in the large yellow croaker, and a majority was conserved with mammals. Clearly, to enrich our knowledge of the adaptive immune response in fish, further studies need to be performed on TCR and BCR signaling pathways.

## Conclusions

In this study, we performed a global transcriptional profiling analysis of poly(I:C)-induced large yellow croakers to explore the antivirus-relevant genes and pathways. Through annotations to the NCBI database, 15,192 identified unigenes were obtained. Our analysis provided a broad overview of the large yellow croaker transcriptome, which contained members of all of the major classes of immune-relevant genes. A considerable amount of immune-relevant genes and pathways in the large yellow croaker showed significant similarities to that in mammals, suggesting that mechanisms underlying the innate and adaptive immunity in fish may be conserved in vertebrates. Meanwhile, a large set of immune-relevant genes revealed major antiviral immunity effectors or factors involved in antiviral pathways. These results provide valuable leads for further investigations into the antiviral immune response of this economically important marine fish.

## Materials and Methods

### Ethics Statement

This study was carried out in strict accordance with the Regulations for the Administration of Affairs Concerning Experimental Animals established by the Fujian Provincial Department of Science and Technology. Animal experiments were approved by the Animal Care and Use Committee of the Third Institute of Oceanography, State Oceanic Administration. All surgeries were performed under Tricaine-S anesthesia, and all efforts were made to minimize suffering.

### Fish and induction experiments

Large yellow croakers (length: 16±1.5 cm; weight: 100±8.6 g) were purchased from the Mari-culture farm in Lianjian, Fuzhou, China. The fish were maintained at 25°C in aerated water tanks (dissolved oxygen concentration: 7.2 mg/L) with a flow-through seawater supply. After 7 days of acclimation, these fish were used for the following experiments. Twenty fish were injected intramuscularly with poly(I:C) at a dose of 0.5 mg/100 g as previously described [1]. Spleens were harvested from 6 fish at 12 h after induction and frozen immediately in liquid nitrogen until RNA extraction and transcriptome analysis were performed.

### RNA isolation

Total RNA was extracted from about 100 mg of spleen tissues from 6 fish with TRIZOL Reagent (Invitrogen, USA), according to the manufacturer's instructions. The RNA samples were incubated for 1 h at 37°C with 10 units of DNaseI (Takara, China) to remove residual genomic DNA. The quality and quantity of the purified RNA was determined by measuring the absorbance at 260 nm/280 nm, using a Nanodrop ND-1000 spectrophotometer (LabTech, USA). The RIN value was 8.9 by the 2100 Bioanalyzer (Agilent Technologies, USA).

### Library preparation and sequencing

To survey the gene expression profile in the spleens of large yellow croakers induced by poly(I:C), a paired-end library was constructed according to the manufacturer's protocol. Polyadenylated RNA was isolated using the Oligotex mRNA Midi Kit (Qiagen Inc., Valencia, CA, USA). Two hundred nanograms of mRNA were used for the library preparation. The RNA-seq library was constructed using Illumina Whole Transcriptome Analysis Kit following the standard protocol (Illumina GA II Sequencing System). An 80 bp paired-end run was performed on the Illumina GAII platform (Illumina, Inc., San Diego, CA, USA) to assemble the whole transcriptome *de novo*. The generated sequence data have been submitted to the NCBI SRA database, and the accession number is SRP035897.

### Assembly of transcripts and annotation

Transcripts were assembled using the SOAP *de novo* software (http://soap.genomics.org.cn/soapdenovo.html). As a result, 108,237 contigs were generated. To annotate these contigs, we first aligned them with the zebrafish RefSeq mRNA database. The remaining non-annotated contigs were further aligned to the non-redundant database [51]. The annotated contigs were clustered and designated as unigenes when two or more query sequences were annotated to the same gene. Gene Ontology was performed using the web-based Database DAVID [26]. Since DAVID only takes gene identifiers from certain species (not including *Pseudosciaena crocea*), we then used gene identifiers of 8843 zebrafish orthologs to perform the functional annotation and 4,759 genes were found to be involved in the three functional categories. KEGG Automatic Annotation Server (KAAS) system was used for pathway reconstruction. 15,192 unigenes were compared against the existing genes in KEGG using Blastx. Those that are most similar to the existing genes are then mapped onto the existing pathways.

### Real-time PCR analysis

Real-time PCR analysis was performed using the Mastercycler epgradient realplex^4^ (Eppendorf, Germany) with SYBR Green as the fluorescent dye, according to the manufacturer's protocol (Takara, China). Primer set was designed based on each identified gene sequence of transcriptome library by Primer Primer 5.0 (**Table S5**). The specificity and amplification efficiency of these primers were tested before real-time PCR. No primers showed dimers in melting curves, and single band was observed on agarose gels **(Fig.S1)**. Total RNA was extracted from spleen tissues of three fish sampled at 0, 12 and 24 h after stimulation with poly(I:C). First-strand cDNA was synthesized from 2 µg total RNA and used as a template for real-time PCR with gene-specific primers. Real-time PCR was performed in a total volume of 20 µL, and cycling conditions were 95°C for 1 min, followed by 40 cycles of 94°C for 5 s, 58°C for 15 s, and 72°C for 20 s. The expression levels of each gene were expressed relative to the expression levels of β-actin in each sample by using the 2^−ΔΔCT^ method [52]. Each real-time PCR assay was repeated three times with different batches of fish. The data of real-time PCR were analyzed using GraphPad Prism 5 software and expressed as the standard error of the mean (SEM). Two-tailed Student's t test was used for the significance test between the experimental group and the control group. A *P*-value <0.05 was considered to be statistically significant.

## Supporting Information

Figure S1 **The agarose gel electrophoresis and melt curve analysis of amplification products of partial genes.**
(DOCX)

Table S1 **The list of 15192 unigenes that were identified in the transcriptome of the large yellow croaker.**
(XLSX)

Table S2 **Gene Ontology function annotation results for the transcriptomic sequences of the large yellow croaker using DAVID.**
(XLSX)

Table S3 **KEGG mapping of the pathways in the large yellow croaker.**
(DOC)

Table S4 **Immune-relevant genes.**
(DOC)

Table S5 **Primers for quantitative real-time PCR.** Primer set was designed based on each identified gene sequence of transcriptome library by Primer Primer 5.0.
(DOC)
