# Correction: *De Novo* Characterization of the Spleen Transcriptome of the Large Yellow Croaker (*Pseudosciaena crocea*) and Analysis of the Immune Relevant Genes and Pathways Involved in the Antiviral Response

**DOI:** 10.1371/journal.pone.0101069

**Published:** 2014-06-24

**Authors:** The PLOS ONE Staff

The images for Figure 5 and Figure 6 are switched. Please see the correct figures and legends here.

**Figure 5:**
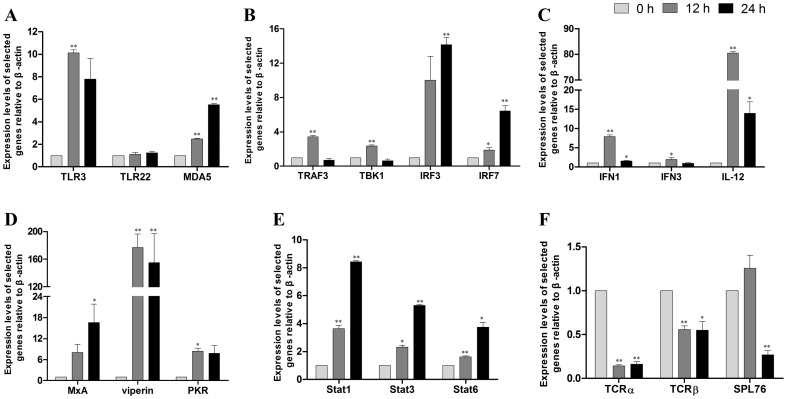
Real-time PCR analysis of selected genes. Total RNA was extracted from the spleens of large yellow croakers sampled at 0, 12 and 24 h after poly(I:C) induction. Real-time PCR was used to validate gene expression changes in the pattern recognition receptors (A), signal transducers (B), interferons and interleukin (C), interferon-stimulated genes (D), JAK-STAT pathway (F), and T-cell receptor (TCR) signaling pathway. Increases and decreases in the relative levels of transcripts with respect to the control β-actin gene are shown.^*^ *P*< 0.05, ^**^ *P*< 0.01.

**Figure 6:**
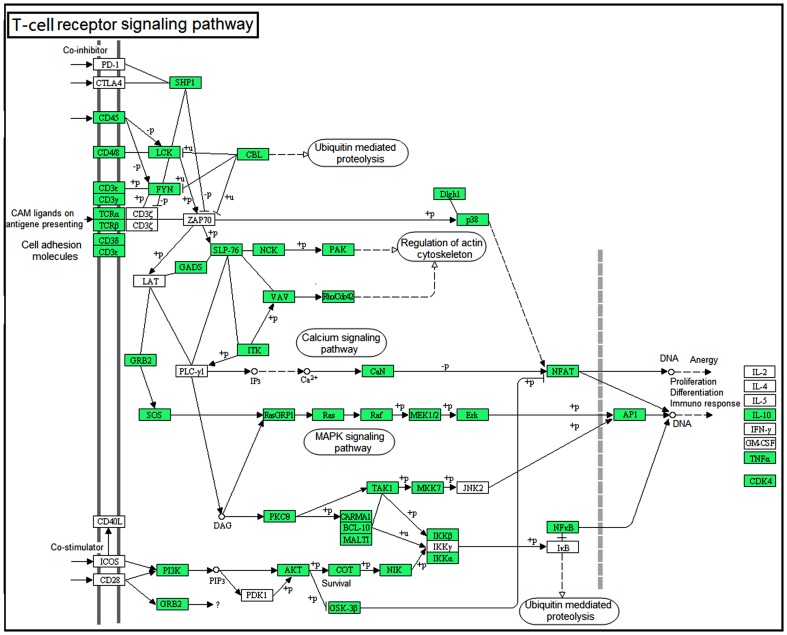
Map of the T-cell receptor signaling pathway, as generated by KEGG. Genes that were identified from the transcriptome of the large yellow croaker spleen are shown in green. White denotes genes that were not identified in the transcriptome analysis.
