# Supplementary material for: De Novo Characterization of the Spleen Transcriptome of the Large Yellow Croaker (Pseudosciaena crocea) and Analysis of the Immune Relevant Genes and Pathways Involved in the Antiviral Response
Source: PLoS One. 2014 May 12;9(5):e97471. doi: 10.1371/journal.pone.0097471 (PMC4018400; doi:10.1371/journal.pone.0097471)
Supplement: Figure S1 — The agarose gel electrophoresis and melt curve analysis of amplification products of partial genes. (DOCX) [file pone.0097471.s001.docx]

1. **β-actin**

**A B**

**M 1 2 3**

**
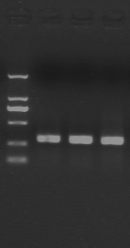
**

1. **TCRβ**

**A B**

**M 1 2 3**


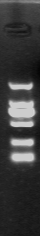

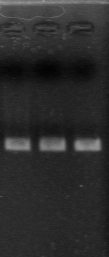


**(3) STAT3**

**A B**

**M 1 2 3**


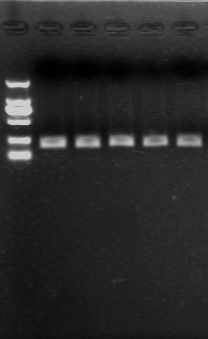
 ****

**(4) IL-12**

**A B**

**1 2 M**


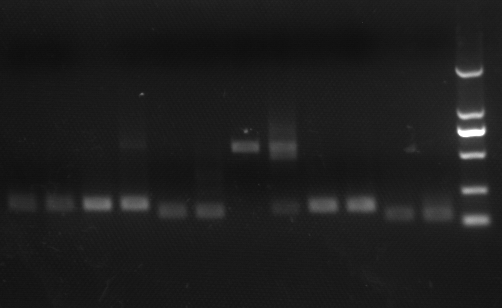

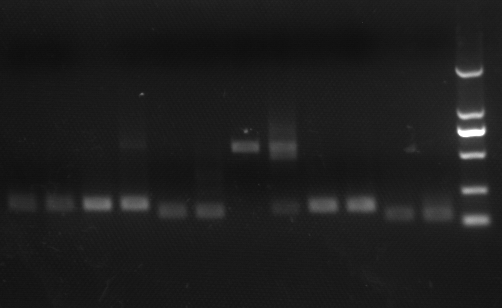


**(5) MDA5**

**A B**

**M 1 2 3**

**
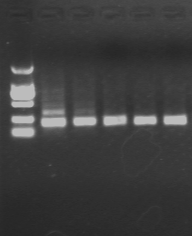

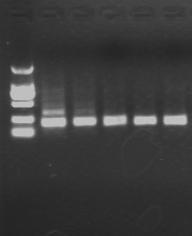
**

**Figure S1. The agarose gel electrophoresis and melt curve analysis of amplification products of partial genes.**

A: The agarose gel electrophoresis analysis. M: marker, 2000 bp, 1000 bp, 750 bp, 500 bp, 250 bp, 100 bp; 1: control; 2: Expression analysis of genes in spleen at 12 h after induction with poly (I: C); 3: Expression analysis of genes in spleen at 24 h after induction with poly (I: C). B: Melt curve analysis
