# Supplementary material for: De Novo Characterization of the Spleen Transcriptome of the Large Yellow Croaker (Pseudosciaena crocea) and Analysis of the Immune Relevant Genes and Pathways Involved in the Antiviral Response
Source: PLoS One. 2014 May 12;9(5):e97471. doi: 10.1371/journal.pone.0097471 (PMC4018400; doi:10.1371/journal.pone.0097471)
Supplement: Table S3 — KEGG mapping of the pathways in the large yellow croaker. (DOC) [file pone.0097471.s004.doc]

Table S3. The pathways in large yellow croaker mapped by KEGG

| NO. | Accession number | KEGG pathway name | Number of genes |
| --- | --- | --- | --- |
| 1 | 00010 | [Glycolysis / Gluconeogenesis](http://www.genome.jp/kegg-bin/mark_pathway_www?@ko00010/reference%3Dwhite/default%3D%23bfffbf/K00844/K01810/K00850/K03841/K01623/K01803/K00134/K00927/K01834/K01689/K00873/K12406/K00161/K00162/K00627/K00382/K00016/K00121/K00002/K00128/K00129/K01895/K01835/K01837/K01596) | 25 |
| 2 | 00020 | [Citrate cycle (TCA cycle)](http://www.genome.jp/kegg-bin/mark_pathway_www?@ko00020/reference%3Dwhite/default%3D%23bfffbf/K01647/K01648/K01681/K00030/K00164/K00658/K00382/K01899/K01900/K00234/K00235/K00236/K00237/K01679/K00025/K00026/K01596/K00161/K00162/K00627) | 20 |
| 3 | 00030 | [Pentose phosphate pathway](http://www.genome.jp/kegg-bin/mark_pathway_www?@ko00030/reference%3Dwhite/default%3D%23bfffbf/K01810/K00036/K01057/K13937/K00033/K01783/K00615/K00616/K01807/K01619/K00852/K01835/K00948/K00851/K01623/K00850/K03841) | 17 |
| 4 | 00040 | [Pentose and glucuronate interconversions](http://www.genome.jp/kegg-bin/mark_pathway_www?@ko00040/reference%3Dwhite/default%3D%23bfffbf/K01195/K00699/K00012/K00963/K13247/K01783/K00854/K01805/K00011) | 9 |
| 5 | 00051 | [Fructose and mannose metabolism](http://www.genome.jp/kegg-bin/mark_pathway_www?@ko00051/reference%3Dwhite/default%3D%23bfffbf/K01809/K01840/K00971/K00966/K01711/K02377/K05305/K00844/K00850/K03841/K01103/K01112/K00846/K00008/K00011/K01805/K01623/K01803) | 18 |
| 6 | 00052 | [Galactose metabolism](http://www.genome.jp/kegg-bin/mark_pathway_www?@ko00052/reference%3Dwhite/default%3D%23bfffbf/K00849/K01784/K00963/K01835/K00844/K12309/K07966/K00965/K01189/K00011/K00850/K12316) | 12 |
| 7 | 00053 | [Ascorbate and aldarate metabolism](http://www.genome.jp/kegg-bin/mark_pathway_www?@ko00053/reference%3Dwhite/default%3D%23bfffbf/K00012/K00699/K00128) | 3 |
| 8 | 00061 | [Fatty acid biosynthesis](http://www.genome.jp/kegg-bin/mark_pathway_www?@ko00061/reference%3Dwhite/default%3D%23bfffbf/K11262/K00645/K00665/K09458/K00059/K01071) | 6 |
| 9 | 00062 | [Fatty acid elongation in mitochondria](http://www.genome.jp/kegg-bin/mark_pathway_www?@ko00062/reference%3Dwhite/default%3D%23bfffbf/K07508/K07509/K00022/K07515/K07511/K01074) | 6 |
| 10 | 00071 | [Fatty acid metabolism](http://www.genome.jp/kegg-bin/mark_pathway_www?@ko00071/reference%3Dwhite/default%3D%23bfffbf/K00626/K07508/K07509/K00022/K07515/K07511/K00232/K00248/K00249/K00255/K09478/K09479/K00252/K01897/K08765/K13238/K00121/K00128) | 18 |
| 11 | 00072 | [Synthesis and degradation of ketone bodies](http://www.genome.jp/kegg-bin/mark_pathway_www?@ko00072/reference%3Dwhite/default%3D%23bfffbf/K01641/K01640/K01027/K00626/K00019) | 5 |
| 12 | 00100 | [Steroid biosynthesis](http://www.genome.jp/kegg-bin/mark_pathway_www?@ko00100/reference%3Dwhite/default%3D%23bfffbf/K00801/K01824/K01052/K00637) | 4 |
| 13 | 00120 | [Primary bile acid biosynthesis](http://www.genome.jp/kegg-bin/mark_pathway_www?@ko00120/reference%3Dwhite/default%3D%23bfffbf/K07430/K07431/K12405) | 3 |
| 14 | 00130 | [Ubiquinone and other terpenoid-quinone biosynthesis](http://www.genome.jp/kegg-bin/mark_pathway_www?@ko00130/reference%3Dwhite/default%3D%23bfffbf/K06125/K06126/K06127/K06134) | 4 |
| 15 | 00140 | [Steroid hormone biosynthesis](http://www.genome.jp/kegg-bin/mark_pathway_www?@ko00140/reference%3Dwhite/default%3D%23bfffbf/K01131/K01015/K00070/K07430/K13368/K13369/K13370/K07424/K07410/K00699) | 10 |
| 16 | 00190 | [Oxidative phosphorylation](http://www.genome.jp/kegg-bin/mark_pathway_www?@ko00190/reference%3Dwhite/default%3D%23bfffbf/K00331/K00337/K03878/K03879/K03883/K03934/K03935/K03936/K03937/K03938/K03939/K03941/K03942/K03945/K03948/K03949/K03950/K03951/K03952/K03953/K03954/K03955/K03956/K03958/K03959/K03960/K03961/K03962/K03963/K03964/K03965/K03966/K03968/K00236/K00237/K00234/K00235/K00411/K00412/K00415/K00416/K00417/K00420/K02262/K02256/K02261/K02263/K02264/K02265/K02266/K02267/K02268/K02270/K02271/K02272/K02258/K02259/K02260/K02112/K02135/K02134/K02136/K02128/K02126/K02127/K02131/K02130/K02140/K02146/K02151/K02148/K02145/K02147/K02150/K02155/K02154/K02149/K02153/K02144/K03662/K01507/K11725) | 82 |
| 17 | 00195 | [Photosynthesis](http://www.genome.jp/kegg-bin/mark_pathway_www?@ko00195/reference%3Dwhite/default%3D%23bfffbf/K02716/K02717/K02694/K02698/K02641/K02112) | 6 |
| 18 | 00196 | [Photosynthesis - antenna proteins](http://www.genome.jp/kegg-bin/mark_pathway_www?@ko00196/reference%3Dwhite/default%3D%23bfffbf/K08907/K08908/K08912) | 3 |
| 19 | 00230 | [Purine metabolism](http://www.genome.jp/kegg-bin/mark_pathway_www?@ko00230/reference%3Dwhite/default%3D%23bfffbf/K01517/K00948/K00764/K11787/K01952/K01587/K01756/K00602/K00759/K01081/K03783/K00760/K00088/K00940/K01510/K12304/K12305/K01511/K01519/K01518/K01951/K00364/K01487/K00942/K00873/K12406/K10807/K10808/K02999/K03000/K03005/K03006/K03010/K03011/K03012/K03013/K03014/K03015/K03016/K03017/K03007/K03008/K03009/K03018/K03021/K03023/K03026/K03027/K03019/K03020/K03022/K03024/K03025/K02320/K02321/K02684/K02685/K02328/K03505/K02324/K02325/K02326/K01514/K08042/K08043/K08046/K08047/K08049/K12319/K01120/K13755/K13296/K13758/K01939/K01490/K00856/K00893/K00939/K01509/K00962/K01522/K13811/K01513) | 83 |
| 20 | 00232 | [Caffeine metabolism](http://www.genome.jp/kegg-bin/mark_pathway_www?@ko00232/reference%3Dwhite/default%3D%23bfffbf/K07409/K00622) | 2 |
| 21 | 00240 | [Pyrimidine metabolism](http://www.genome.jp/kegg-bin/mark_pathway_www?@ko00240/reference%3Dwhite/default%3D%23bfffbf/K00254/K13421/K13800/K13809/K00940/K00962/K01510/K12304/K12305/K01511/K01519/K01937/K02999/K03000/K03005/K03006/K03010/K03011/K03012/K03013/K03014/K03015/K03016/K03017/K03007/K03008/K03009/K03018/K03021/K03023/K03026/K03027/K03019/K03020/K03022/K03024/K03025/K02320/K02321/K02684/K02685/K02328/K03505/K02324/K02325/K02326/K00876/K01081/K00757/K00761/K01464/K00384/K10807/K10808/K01520/K00560/K03783/K00893/K01493/K00857/K01518) | 61 |
| 22 | 00250 | [Alanine, aspartate and glutamate metabolism](http://www.genome.jp/kegg-bin/mark_pathway_www?@ko00250/reference%3Dwhite/default%3D%23bfffbf/K00813/K03334/K01953/K13566/K00814/K01940/K01755/K01939/K01756/K01580/K13524/K00135/K00139/K00261/K00294/K01915/K01425/K01948/K00820/K00764) | 20 |
| 23 | 00260 | [Glycine, serine and threonine metabolism](http://www.genome.jp/kegg-bin/mark_pathway_www?@ko00260/reference%3Dwhite/default%3D%23bfffbf/K00600/K00831/K00639/K00643/K00281/K00605/K00382/K00542/K00108/K00314/K00552/K01697/K01758) | 13 |
| 24 | 00270 | [Cysteine and methionine metabolism](http://www.genome.jp/kegg-bin/mark_pathway_www?@ko00270/reference%3Dwhite/default%3D%23bfffbf/K01758/K00548/K00789/K00797/K00802/K00772/K08964/K09880/K08967/K03334/K00558/K01251/K01697/K00456/K00813/K01011/K00016) | 17 |
| 25 | 00280 | [Valine, leucine and isoleucine degradation](http://www.genome.jp/kegg-bin/mark_pathway_www?@ko00280/reference%3Dwhite/default%3D%23bfffbf/K00826/K03334/K00166/K00167/K09699/K00382/K00248/K00249/K00253/K09478/K11538/K07515/K07511/K00022/K08683/K07508/K07509/K01966/K05606/K01847/K05605/K00020/K00140/K00128/K13524/K01968/K05607/K01640/K01027/K00626/K01641) | 31 |
| 26 | 00281 | [Geraniol degradation](http://www.genome.jp/kegg-bin/mark_pathway_www?@ko00281/reference%3Dwhite/default%3D%23bfffbf/K01640/K00257/K00022) | 3 |
| 27 | 00290 | [Valine, leucine and isoleucine biosynthesis](http://www.genome.jp/kegg-bin/mark_pathway_www?@ko00290/reference%3Dwhite/default%3D%23bfffbf/K00826/K01869/K01870/K01873/K00161/K00162) | 6 |
| 28 | 00300 | [Lysine biosynthesis](http://www.genome.jp/kegg-bin/mark_pathway_www?@ko00300/reference%3Dwhite/default%3D%23bfffbf/K00142/K00290) | 2 |
| 29 | 00310 | [Lysine degradation](http://www.genome.jp/kegg-bin/mark_pathway_www?@ko00310/reference%3Dwhite/default%3D%23bfffbf/K00290/K00142/K00164/K00658/K00252/K07515/K07511/K00022/K00626/K06101/K11427/K11420/K11424/K11422/K11423/K11431/K11428/K11419/K11429/K00128/K00471/K00473/K13645/K11703) | 24 |
| 30 | 00311 | [Penicillin and cephalosporin biosynthesis](http://www.genome.jp/kegg-bin/mark_pathway_www?@ko00311/reference%3Dwhite/default%3D%23bfffbf/K01467) | 1 |
| 31 | 00312 | [beta-Lactam resistance](http://www.genome.jp/kegg-bin/mark_pathway_www?@ko00312/reference%3Dwhite/default%3D%23bfffbf/K01467) | 1 |
| 32 | 00330 | [Arginine and proline metabolism](http://www.genome.jp/kegg-bin/mark_pathway_www?@ko00330/reference%3Dwhite/default%3D%23bfffbf/K00542/K00933/K00797/K00802/K11182/K00128/K00657/K00819/K00318/K00294/K12657/K11142/K00472/K00813/K01940/K01755/K01476/K01425/K01915/K00261/K01948/K01436) | 22 |
| 33 | 00340 | [Histidine metabolism](http://www.genome.jp/kegg-bin/mark_pathway_www?@ko00340/reference%3Dwhite/default%3D%23bfffbf/K01593/K11182/K00128/K00129/K00599) | 5 |
| 34 | 00350 | [Tyrosine metabolism](http://www.genome.jp/kegg-bin/mark_pathway_www?@ko00350/reference%3Dwhite/default%3D%23bfffbf/K00813/K03334/K01800/K01555/K00506/K01593/K00129/K00121/K00135/K00599/K07253) | 11 |
| 35 | 00360 | [Phenylalanine metabolism](http://www.genome.jp/kegg-bin/mark_pathway_www?@ko00360/reference%3Dwhite/default%3D%23bfffbf/K01593/K00129/K00813/K03334/K07253/K11188) | 6 |
| 36 | 00361 | [gamma-Hexachlorocyclohexane degradation](http://www.genome.jp/kegg-bin/mark_pathway_www?@ko00361/reference%3Dwhite/default%3D%23bfffbf/K07424/K01078/K01077) | 3 |
| 37 | 00362 | [Benzoate degradation via hydroxylation](http://www.genome.jp/kegg-bin/mark_pathway_www?@ko00362/reference%3Dwhite/default%3D%23bfffbf/K07508/K07509) | 2 |
| 38 | 00380 | [Tryptophan metabolism](http://www.genome.jp/kegg-bin/mark_pathway_www?@ko00380/reference%3Dwhite/default%3D%23bfffbf/K00463/K01432/K01556/K00452/K00164/K00252/K07515/K07511/K00022/K00626/K00816/K01593/K00128/K07409/K07410/K01867/K03334/K11182/K03781) | 19 |
| 39 | 00400 | [Phenylalanine, tyrosine and tryptophan biosynthesis](http://www.genome.jp/kegg-bin/mark_pathway_www?@ko00400/reference%3Dwhite/default%3D%23bfffbf/K00813/K03334) | 2 |
| 40 | 00401 | [Novobiocin biosynthesis](http://www.genome.jp/kegg-bin/mark_pathway_www?@ko00401/reference%3Dwhite/default%3D%23bfffbf/K00813) | 1 |
| 41 | 00410 | [beta-Alanine metabolism](http://www.genome.jp/kegg-bin/mark_pathway_www?@ko00410/reference%3Dwhite/default%3D%23bfffbf/K01580/K00128/K13524/K00797/K00802/K01464/K05605/K07515/K07511/K00249) | 10 |
| 42 | 00430 | [Taurine and hypotaurine metabolism](http://www.genome.jp/kegg-bin/mark_pathway_www?@ko00430/reference%3Dwhite/default%3D%23bfffbf/K00456/K01580/K10712/K00681) | 4 |
| 43 | 00440 | [Phosphonate and phosphinate metabolism](http://www.genome.jp/kegg-bin/mark_pathway_www?@ko00440/reference%3Dwhite/default%3D%23bfffbf/K00967/K00993/K13644/K00968) | 4 |
| 44 | 00450 | [Selenoamino acid metabolism](http://www.genome.jp/kegg-bin/mark_pathway_www?@ko00450/reference%3Dwhite/default%3D%23bfffbf/K00681/K01758/K01697/K01251/K00599/K00789/K01874/K13811/K01763/K01008) | 10 |
| 45 | 00460 | [Cyanoamino acid metabolism](http://www.genome.jp/kegg-bin/mark_pathway_www?@ko00460/reference%3Dwhite/default%3D%23bfffbf/K00681/K00600) | 2 |
| 46 | 00471 | [D-Glutamine and D-glutamate metabolism](http://www.genome.jp/kegg-bin/mark_pathway_www?@ko00471/reference%3Dwhite/default%3D%23bfffbf/K01425/K00261) | 2 |
| 47 | 00480 | [Glutathione metabolism](http://www.genome.jp/kegg-bin/mark_pathway_www?@ko00480/reference%3Dwhite/default%3D%23bfffbf/K00681/K11204/K11205/K01920/K11142/K01255/K00799/K13299/K00383/K00033/K00036/K05360/K00432/K00797/K00802/K10807/K10808) | 17 |
| 48 | 00500 | [Starch and sucrose metabolism](http://www.genome.jp/kegg-bin/mark_pathway_www?@ko00500/reference%3Dwhite/default%3D%23bfffbf/K12316/K01194/K00012/K00699/K01195/K00963/K01835/K00844/K11809/K01810/K00700/K00688/K00693/K01196/K01513) | 15 |
| 49 | 00510 | [N-Glycan biosynthesis](http://www.genome.jp/kegg-bin/mark_pathway_www?@ko00510/reference%3Dwhite/default%3D%23bfffbf/K01001/K00729/K07432/K07441/K00721/K09659/K03843/K03844/K06316/K03845/K03846/K03847/K03848/K03849/K03850/K07151/K12666/K12667/K12668/K12669/K12670/K07252/K01228/K01230/K00726/K01231/K00736/K00717/K07966/K07968/K00778/K00737/K00738/K00744/K13748) | 35 |
| 50 | 00511 | [Other glycan degradation](http://www.genome.jp/kegg-bin/mark_pathway_www?@ko00511/reference%3Dwhite/default%3D%23bfffbf/K01186/K12309/K12373/K01191/K12312/K01192/K01227/K01206/K01444/K01201) | 10 |
| 51 | 00512 | [O-Glycan biosynthesis](http://www.genome.jp/kegg-bin/mark_pathway_www?@ko00512/reference%3Dwhite/default%3D%23bfffbf/K00710/K00731/K09653/K03368/K09905) | 5 |
| 52 | 00513 | [High-mannose type N-glycan biosynthesis](http://www.genome.jp/kegg-bin/mark_pathway_www?@ko00513/reference%3Dwhite/default%3D%23bfffbf/K01230) | 1 |
| 53 | 00514 | [O-Mannosyl glycan biosynthesis](http://www.genome.jp/kegg-bin/mark_pathway_www?@ko00514/reference%3Dwhite/default%3D%23bfffbf/K00728/K09666) | 2 |
| 54 | 00520 | [Amino sugar and nucleotide sugar metabolism](http://www.genome.jp/kegg-bin/mark_pathway_www?@ko00520/reference%3Dwhite/default%3D%23bfffbf/K12373/K00884/K01836/K00972/K12409/K01787/K05304/K01639/K00326/K00844/K01443/K00621/K02564/K00820/K01810/K01835/K00963/K00965/K00012/K00849/K01784/K01840/K00966/K00971/K01711/K01809/K05305/K02377) | 28 |
| 55 | 00521 | [Streptomycin biosynthesis](http://www.genome.jp/kegg-bin/mark_pathway_www?@ko00521/reference%3Dwhite/default%3D%23bfffbf/K00844/K01835/K01858/K01092/K01710) | 5 |
| 56 | 00523 | [Polyketide sugar unit biosynthesis](http://www.genome.jp/kegg-bin/mark_pathway_www?@ko00523/reference%3Dwhite/default%3D%23bfffbf/K01710) | 1 |
| 57 | 00524 | [Butirosin and neomycin biosynthesis](http://www.genome.jp/kegg-bin/mark_pathway_www?@ko00524/reference%3Dwhite/default%3D%23bfffbf/K00844) | 1 |
| 58 | 00531 | [Glycosaminoglycan degradation](http://www.genome.jp/kegg-bin/mark_pathway_www?@ko00531/reference%3Dwhite/default%3D%23bfffbf/K01136/K01217/K01135/K01197/K01195/K07964/K01565/K10532/K01205/K12309/K01137/K12373) | 12 |
| 59 | 00532 | [Glycosaminoglycan biosynthesis - chondroitin sulfate](http://www.genome.jp/kegg-bin/mark_pathway_www?@ko00532/reference%3Dwhite/default%3D%23bfffbf/K00771/K00733/K10158/K00746/K13499/K00747/K04742/K04743/K01794/K08105/K03193/K08106) | 12 |
| 60 | 00533 | [Glycosaminoglycan biosynthesis - keratan sulfate](http://www.genome.jp/kegg-bin/mark_pathway_www?@ko00533/reference%3Dwhite/default%3D%23bfffbf/K00717/K07966/K07968/K00741/K09671/K09664/K00781/K03368/K03494) | 9 |
| 61 | 00534 | [Glycosaminoglycan biosynthesis - heparan sulfate](http://www.genome.jp/kegg-bin/mark_pathway_www?@ko00534/reference%3Dwhite/default%3D%23bfffbf/K00771/K00733/K10158/K02369/K02370/K02366/K02367/K02576/K02577/K02578/K02579/K01793/K02514/K07809) | 14 |
| 62 | 00561 | [Glycerolipid metabolism](http://www.genome.jp/kegg-bin/mark_pathway_www?@ko00561/reference%3Dwhite/default%3D%23bfffbf/K00128/K00011/K00002/K00864/K13506/K13509/K13513/K13523/K01080/K00901/K11155/K01046/K01059/K09881/K01189) | 15 |
| 63 | 00562 | [Inositol phosphate metabolism](http://www.genome.jp/kegg-bin/mark_pathway_www?@ko00562/reference%3Dwhite/default%3D%23bfffbf/K00914/K00888/K13711/K00889/K01099/K05858/K05857/K05860/K05859/K00922/K01110/K00920/K00923/K01109/K00921/K01092/K00999/K01858/K01107/K01106/K03103/K00911/K00328/K10572/K00140/K01803) | 26 |
| 64 | 00563 | [Glycosylphosphatidylinositol(GPI)-anchor biosynthesis](http://www.genome.jp/kegg-bin/mark_pathway_www?@ko00563/reference%3Dwhite/default%3D%23bfffbf/K03857/K03858/K03859/K03861/K07542/K05286/K05289/K05290/K05292/K05293/K05294/K05310) | 12 |
| 65 | 00564 | [Glycerophospholipid metabolism](http://www.genome.jp/kegg-bin/mark_pathway_www?@ko00564/reference%3Dwhite/default%3D%23bfffbf/K00006/K00111/K13506/K13509/K13513/K13523/K00649/K01517/K01080/K00901/K13644/K01115/K01047/K13510/K13515/K06128/K06129/K06130/K00866/K00968/K00993/K00894/K00967/K00981/K08730/K01613/K00995/K08744/K13511/K13514/K00999/K13516) | 32 |
| 66 | 00565 | [Ether lipid metabolism](http://www.genome.jp/kegg-bin/mark_pathway_www?@ko00565/reference%3Dwhite/default%3D%23bfffbf/K00803/K01080/K00993/K13644/K01047/K01122/K01115/K13510/K01062) | 9 |
| 67 | 00590 | [Arachidonic acid metabolism](http://www.genome.jp/kegg-bin/mark_pathway_www?@ko00590/reference%3Dwhite/default%3D%23bfffbf/K01047/K08021/K00432/K07418/K00461/K00681/K00490/K11987/K01831/K01832/K05309/K08022/K07422) | 13 |
| 68 | 00591 | [Linoleic acid metabolism](http://www.genome.jp/kegg-bin/mark_pathway_www?@ko00591/reference%3Dwhite/default%3D%23bfffbf/K01047/K07409/K07418/K07424) | 4 |
| 69 | 00592 | [alpha-Linolenic acid metabolism](http://www.genome.jp/kegg-bin/mark_pathway_www?@ko00592/reference%3Dwhite/default%3D%23bfffbf/K01047/K00232) | 2 |
| 70 | 00600 | [Sphingolipid metabolism](http://www.genome.jp/kegg-bin/mark_pathway_www?@ko00600/reference%3Dwhite/default%3D%23bfffbf/K00654/K04708/K12348/K01441/K04711/K04712/K04714/K12351/K12353/K12354/K04715/K01080/K04716/K04718/K01634/K00720/K01201/K07553/K12309/K01202/K01186/K01189) | 22 |
| 71 | 00601 | [Glycosphingolipid biosynthesis - lacto and neolacto series](http://www.genome.jp/kegg-bin/mark_pathway_www?@ko00601/reference%3Dwhite/default%3D%23bfffbf/K03766/K07820/K00781/K03494/K00709/K07966/K07968/K07635/K00741/K07970/K00742) | 11 |
| 72 | 00603 | [Glycosphingolipid biosynthesis - globo series](http://www.genome.jp/kegg-bin/mark_pathway_www?@ko00603/reference%3Dwhite/default%3D%23bfffbf/K01189/K12373/K00722/K03368) | 4 |
| 73 | 00604 | [Glycosphingolipid biosynthesis - ganglio series](http://www.genome.jp/kegg-bin/mark_pathway_www?@ko00604/reference%3Dwhite/default%3D%23bfffbf/K00725/K03368/K06615/K03370/K03372/K06616/K03376/K12309/K12373) | 9 |
| 74 | 00620 | [Pyruvate metabolism](http://www.genome.jp/kegg-bin/mark_pathway_www?@ko00620/reference%3Dwhite/default%3D%23bfffbf/K01895/K00161/K00162/K00382/K00627/K00873/K12406/K11262/K01512/K00128/K00016/K01759/K01069/K00049/K00011/K00027/K00029/K00025/K00026/K01596/K00626) | 21 |
| 75 | 00624 | [1- and 2-Methylnaphthalene degradation](http://www.genome.jp/kegg-bin/mark_pathway_www?@ko00624/reference%3Dwhite/default%3D%23bfffbf/K00121/K00257) | 2 |
| 76 | 00626 | [Naphthalene and anthracene degradation](http://www.genome.jp/kegg-bin/mark_pathway_www?@ko00626/reference%3Dwhite/default%3D%23bfffbf/K00599) | 1 |
| 77 | 00630 | [Glyoxylate and dicarboxylate metabolism](http://www.genome.jp/kegg-bin/mark_pathway_www?@ko00630/reference%3Dwhite/default%3D%23bfffbf/K00025/K00026/K01647/K01681/K00049/K01602/K01816/K00288/K13403/K01432) | 10 |
| 78 | 00631 | [1,2-Dichloroethane degradation](http://www.genome.jp/kegg-bin/mark_pathway_www?@ko00631/reference%3Dwhite/default%3D%23bfffbf/K00128) | 1 |
| 79 | 00632 | [Benzoate degradation via CoA ligation](http://www.genome.jp/kegg-bin/mark_pathway_www?@ko00632/reference%3Dwhite/default%3D%23bfffbf/K01512/K00252/K07515/K07511/K00626) | 5 |
| 80 | 00633 | [Trinitrotoluene degradation](http://www.genome.jp/kegg-bin/mark_pathway_www?@ko00633/reference%3Dwhite/default%3D%23bfffbf/K00622) | 1 |
| 81 | 00640 | [Propanoate metabolism](http://www.genome.jp/kegg-bin/mark_pathway_www?@ko00640/reference%3Dwhite/default%3D%23bfffbf/K00626/K01895/K01908/K00249/K07515/K07511/K05605/K11262/K13524/K01966/K05606/K01847/K01899/K01900/K00140/K00128/K00016) | 17 |
| 82 | 00641 | [3-Chloroacrylic acid degradation](http://www.genome.jp/kegg-bin/mark_pathway_www?@ko00641/reference%3Dwhite/default%3D%23bfffbf/K00121/K00128) | 2 |
| 83 | 00643 | [Styrene degradation](http://www.genome.jp/kegg-bin/mark_pathway_www?@ko00643/reference%3Dwhite/default%3D%23bfffbf/K01800/K01555) | 2 |
| 84 | 00650 | [Butanoate metabolism](http://www.genome.jp/kegg-bin/mark_pathway_www?@ko00650/reference%3Dwhite/default%3D%23bfffbf/K00626/K00022/K07515/K07511/K00248/K00109/K00135/K00139/K13524/K01580/K01641/K01640/K01027/K00019/K00128/K00161/K00162) | 17 |
| 85 | 00670 | [One carbon pool by folate](http://www.genome.jp/kegg-bin/mark_pathway_www?@ko00670/reference%3Dwhite/default%3D%23bfffbf/K00288/K13403/K00600/K11787/K00602/K00604/K00605/K00548/K00560/K00289/K00297/K01934) | 12 |
| 86 | 00680 | [Methane metabolism](http://www.genome.jp/kegg-bin/mark_pathway_www?@ko00680/reference%3Dwhite/default%3D%23bfffbf/K03781/K11188/K00121/K01070/K00297/K00600) | 6 |
| 87 | 00710 | [Carbon fixation in photosynthetic organisms](http://www.genome.jp/kegg-bin/mark_pathway_www?@ko00710/reference%3Dwhite/default%3D%23bfffbf/K01602/K00927/K01623/K03841/K00615/K01807/K01803/K01783/K00813/K00873/K00814/K00029/K00025/K00026) | 14 |
| 88 | 00720 | [Reductive carboxylate cycle (CO2 fixation)](http://www.genome.jp/kegg-bin/mark_pathway_www?@ko00720/reference%3Dwhite/default%3D%23bfffbf/K01648/K01679/K01681/K01895) | 4 |
| 89 | 00730 | [Thiamine metabolism](http://www.genome.jp/kegg-bin/mark_pathway_www?@ko00730/reference%3Dwhite/default%3D%23bfffbf/K04487/K01112/K00949) | 3 |
| 90 | 00740 | [Riboflavin metabolism](http://www.genome.jp/kegg-bin/mark_pathway_www?@ko00740/reference%3Dwhite/default%3D%23bfffbf/K01112/K00861/K00953/K01078/K01513) | 5 |
| 91 | 00750 | [Vitamin B6 metabolism](http://www.genome.jp/kegg-bin/mark_pathway_www?@ko00750/reference%3Dwhite/default%3D%23bfffbf/K00868/K13248/K00831) | 3 |
| 92 | 00760 | [Nicotinate and nicotinamide metabolism](http://www.genome.jp/kegg-bin/mark_pathway_www?@ko00760/reference%3Dwhite/default%3D%23bfffbf/K06210/K01950/K01242/K03462/K03783/K01081/K01513/K03426/K00324/K00858) | 10 |
| 93 | 00770 | [Pantothenate and CoA biosynthesis](http://www.genome.jp/kegg-bin/mark_pathway_www?@ko00770/reference%3Dwhite/default%3D%23bfffbf/K09680/K01598/K01513/K02318/K00859/K00826/K01464) | 7 |
| 94 | 00780 | [Biotin metabolism](http://www.genome.jp/kegg-bin/mark_pathway_www?@ko00780/reference%3Dwhite/default%3D%23bfffbf/K01942) | 1 |
| 95 | 00785 | [Lipoic acid metabolism](http://www.genome.jp/kegg-bin/mark_pathway_www?@ko00785/reference%3Dwhite/default%3D%23bfffbf/K03644/K03801) | 2 |
| 96 | 00790 | [Folate biosynthesis](http://www.genome.jp/kegg-bin/mark_pathway_www?@ko00790/reference%3Dwhite/default%3D%23bfffbf/K01495/K01077/K01930/K01307/K01737/K00357) | 6 |
| 97 | 00830 | [Retinol metabolism](http://www.genome.jp/kegg-bin/mark_pathway_www?@ko00830/reference%3Dwhite/default%3D%23bfffbf/K00515/K00121/K11153/K11155/K07437/K12664/K07409/K07424/K00699/K09516) | 10 |
| 98 | 00860 | [Porphyrin and chlorophyll metabolism](http://www.genome.jp/kegg-bin/mark_pathway_www?@ko00860/reference%3Dwhite/default%3D%23bfffbf/K00643/K01885/K01698/K01749/K01719/K01599/K00228/K00231/K01772/K02259/K00510/K00214/K05901/K00699/K01195/K01764/K00522) | 17 |
| 99 | 00900 | [Terpenoid backbone biosynthesis](http://www.genome.jp/kegg-bin/mark_pathway_www?@ko00900/reference%3Dwhite/default%3D%23bfffbf/K00626/K01641/K00021/K13273/K01597/K01823/K00787/K00804/K12504/K12505/K11778) | 11 |
| 100 | 00901 | [Indole alkaloid biosynthesis](http://www.genome.jp/kegg-bin/mark_pathway_www?@ko00901/reference%3Dwhite/default%3D%23bfffbf/K01593) | 1 |
| 101 | 00903 | [Limonene and pinene degradation](http://www.genome.jp/kegg-bin/mark_pathway_www?@ko00903/reference%3Dwhite/default%3D%23bfffbf/K00128/K07515/K07511) | 3 |
| 102 | 00910 | [Nitrogen metabolism](http://www.genome.jp/kegg-bin/mark_pathway_www?@ko00910/reference%3Dwhite/default%3D%23bfffbf/K01672/K01948/K00605/K00261/K01915/K01425/K01953/K01758) | 8 |
| 103 | 00920 | [Sulfur metabolism](http://www.genome.jp/kegg-bin/mark_pathway_www?@ko00920/reference%3Dwhite/default%3D%23bfffbf/K13811/K00380/K01015/K04742/K00387) | 5 |
| 104 | 00930 | [Caprolactam degradation](http://www.genome.jp/kegg-bin/mark_pathway_www?@ko00930/reference%3Dwhite/default%3D%23bfffbf/K00002/K07515/K07511/K00022) | 4 |
| 105 | 00940 | [Phenylpropanoid biosynthesis](http://www.genome.jp/kegg-bin/mark_pathway_www?@ko00940/reference%3Dwhite/default%3D%23bfffbf/K11188) | 1 |
| 106 | 00944 | [Flavone and flavonol biosynthesis](http://www.genome.jp/kegg-bin/mark_pathway_www?@ko00944/reference%3Dwhite/default%3D%23bfffbf/K01195) | 1 |
| 107 | 00950 | [Isoquinoline alkaloid biosynthesis](http://www.genome.jp/kegg-bin/mark_pathway_www?@ko00950/reference%3Dwhite/default%3D%23bfffbf/K03334/K00813/K01593) | 3 |
| 108 | 00960 | [Tropane, piperidine and pyridine alkaloid biosynthesis](http://www.genome.jp/kegg-bin/mark_pathway_www?@ko00960/reference%3Dwhite/default%3D%23bfffbf/K00813) | 1 |
| 109 | 00965 | [Betalain biosynthesis](http://www.genome.jp/kegg-bin/mark_pathway_www?@ko00965/reference%3Dwhite/default%3D%23bfffbf/K01593) | 1 |
| 110 | 00970 | [Aminoacyl-tRNA biosynthesis](http://www.genome.jp/kegg-bin/mark_pathway_www?@ko00970/reference%3Dwhite/default%3D%23bfffbf/K01885/K02433/K02434/K01886/K01872/K01876/K01893/K01880/K01868/K01875/K10837/K03341/K01883/K01874/K00604/K01873/K01869/K01870/K04567/K01887/K01881/K01892/K01889/K01890/K01866/K01867) | 26 |
| 111 | 00980 | [Metabolism of xenobiotics by cytochrome P450](http://www.genome.jp/kegg-bin/mark_pathway_www?@ko00980/reference%3Dwhite/default%3D%23bfffbf/K07409/K07410/K07424/K00799/K13299/K01253/K00129/K00121/K00699) | 9 |
| 112 | 00982 | [Drug metabolism - cytochrome P450](http://www.genome.jp/kegg-bin/mark_pathway_www?@ko00982/reference%3Dwhite/default%3D%23bfffbf/K07424/K00485/K00799/K13299/K00121/K00129/K00699/K07409) | 8 |
| 113 | 00983 | [Drug metabolism - other enzymes](http://www.genome.jp/kegg-bin/mark_pathway_www?@ko00983/reference%3Dwhite/default%3D%23bfffbf/K00760/K00088/K01951/K00569/K01519/K03927/K00699/K01195/K07424/K01464/K00757/K00876/K00857/K13421/K00622) | 15 |
| 114 | 01040 | [Biosynthesis of unsaturated fatty acids](http://www.genome.jp/kegg-bin/mark_pathway_www?@ko01040/reference%3Dwhite/default%3D%23bfffbf/K10203/K10251/K10258/K00059/K00507/K00232/K07515/K01068) | 8 |
| 115 | 01051 | [Biosynthesis of ansamycins](http://www.genome.jp/kegg-bin/mark_pathway_www?@ko01051/reference%3Dwhite/default%3D%23bfffbf/K00615) | 1 |
| 116 | 01055 | [Biosynthesis of vancomycin group antibiotics](http://www.genome.jp/kegg-bin/mark_pathway_www?@ko01055/reference%3Dwhite/default%3D%23bfffbf/K01710) | 1 |
| 117 | 02010 | [ABC transporters](http://www.genome.jp/kegg-bin/mark_pathway_www?@ko02010/reference%3Dwhite/default%3D%23bfffbf/K02035/K05641/K05642/K05643/K05648/K05653/K05657/K05661/K05665/K05672/K05673/K05677/K05678/K05681) | 14 |
| 118 | 02020 | [Two-component system](http://www.genome.jp/kegg-bin/mark_pathway_www?@ko02020/reference%3Dwhite/default%3D%23bfffbf/K01077/K02259/K00027/K01467/K01915/K00626) | 6 |
| 119 | 03010 | [Ribosome](http://www.genome.jp/kegg-bin/mark_pathway_www?@ko03010/reference%3Dwhite/default%3D%23bfffbf/K02992/K02948/K02950/K02985/K02987/K02991/K02993/K02995/K02947/K02953/K02957/K02962/K02964/K02966/K02974/K02975/K02976/K02978/K02979/K02980/K02998/K02906/K02871/K02879/K02887/K02899/K02925/K02932/K02934/K02936/K02940/K02866/K02868/K02873/K02872/K02875/K02880/K02883/K02882/K02891/K02894/K02893/K02896/K02898/K02900/K02903/K02908/K02910/K02915/K02920/K02921/K02923/K02924/K02927/K02941/K02943) | 56 |
| 120 | 03018 | [RNA degradation](http://www.genome.jp/kegg-bin/mark_pathway_www?@ko03018/reference%3Dwhite/default%3D%23bfffbf/K12584/K07573/K03679/K03681/K12585/K12586/K12587/K12589/K11600/K12590/K03678/K12591/K12592/K12593/K03514/K12597/K12598/K12599/K12600/K12603/K12604/K12605/K12581/K12606/K12607/K01148/K12613/K12614/K12615/K12616/K12619/K12621/K12622/K12623/K12624/K12625/K12626/K12627/K01689/K00962/K04043/K04077/K00970) | 43 |
| 121 | 03020 | [RNA polymerase](http://www.genome.jp/kegg-bin/mark_pathway_www?@ko03020/reference%3Dwhite/default%3D%23bfffbf/K03006/K03010/K03011/K03012/K03013/K03014/K03015/K03016/K03017/K03007/K03008/K03009/K03018/K03021/K03023/K03026/K03027/K03019/K03020/K03022/K03024/K03025/K02999/K03000/K03005) | 25 |
| 122 | 03022 | [Basal transcription factors](http://www.genome.jp/kegg-bin/mark_pathway_www?@ko03022/reference%3Dwhite/default%3D%23bfffbf/K03120/K03125/K03128/K03129/K03130/K03131/K03132/K03133/K03134/K03135/K03126/K03127/K03124/K03122/K03123/K03121/K03138/K03139/K03136/K03137/K03141/K03142/K03143/K03144) | 24 |
| 123 | 03030 | [DNA replication](http://www.genome.jp/kegg-bin/mark_pathway_www?@ko03030/reference%3Dwhite/default%3D%23bfffbf/K03111/K03469/K02320/K02321/K02684/K02685/K02328/K03505/K02324/K02325/K02326/K02540/K02209/K02542/K02210/K07466/K10739/K10740/K04802/K10754/K10755/K10756/K10743/K10744/K10745/K04799/K10747) | 27 |
| 124 | 03040 | [Spliceosome](http://www.genome.jp/kegg-bin/mark_pathway_www?@ko03040/reference%3Dwhite/default%3D%23bfffbf/K12812/K12815/K12816/K12817/K12818/K12819/K12820/K11087/K11096/K11088/K11097/K11098/K11099/K11093/K11095/K12821/K12822/K12823/K12824/K11092/K11094/K12825/K12826/K12827/K12828/K12829/K12830/K12831/K12833/K12834/K12835/K12836/K12837/K12838/K12839/K12840/K12841/K12842/K12621/K12622/K12623/K12624/K12625/K12626/K12627/K12843/K12662/K09567/K12844/K12845/K12846/K12847/K11984/K12848/K12850/K12855/K12856/K12858/K12859/K10599/K12860/K12861/K12862/K12863/K12864/K03283/K12865/K06063/K12867/K12868/K12869/K12870/K12733/K09564/K12871/K12872/K12873/K12874/K12875/K12876/K12877/K12879/K12880/K12881/K12882/K12883/K12741/K12884/K12886/K12887/K12890/K12891/K12892/K12893/K12896/K12900) | 96 |
| 125 | 03050 | [Proteasome](http://www.genome.jp/kegg-bin/mark_pathway_www?@ko03050/reference%3Dwhite/default%3D%23bfffbf/K03033/K03035/K03036/K03037/K03038/K03039/K03031/K03029/K03028/K03032/K03062/K03066/K03064/K03065/K03063/K06698/K06699/K02730/K02728/K02731/K02725/K02738/K02739/K02735/K02734/K02737/K02736/K02741/K02740/K11599) | 30 |
| 126 | 03060 | [Protein export](http://www.genome.jp/kegg-bin/mark_pathway_www?@ko03060/reference%3Dwhite/default%3D%23bfffbf/K03070/K10956/K09481/K07342/K12275/K09540/K09490/K03109/K03104/K03108/K03107/K03105/K13431/K12946/K12947/K12948/K13280/K09647/K09648) | 19 |
| 127 | 03070 | [Bacterial secretion system](http://www.genome.jp/kegg-bin/mark_pathway_www?@ko03070/reference%3Dwhite/default%3D%23bfffbf/K03070) | 1 |
| 128 | 03320 | [PPAR signaling pathway](http://www.genome.jp/kegg-bin/mark_pathway_www?@ko03320/reference%3Dwhite/default%3D%23bfffbf/K08745/K08746/K08747/K08749/K08752/K08756/K08524/K08525/K04504/K08530/K00507/K07431/K08762/K01059/K01897/K00232/K08765/K00255/K00249/K06086/K06272/K06276/K01596/K00864) | 24 |
| 129 | 03410 | [Base excision repair](http://www.genome.jp/kegg-bin/mark_pathway_www?@ko03410/reference%3Dwhite/default%3D%23bfffbf/K03660/K10567/K03648/K03649/K03575/K03652/K01142/K01151/K10771/K02330/K03512/K10802/K10803/K10776/K10798/K04802/K02328/K03505/K02324/K02325/K02326/K04799/K10747) | 23 |
| 130 | 03420 | [Nucleotide excision repair](http://www.genome.jp/kegg-bin/mark_pathway_www?@ko03420/reference%3Dwhite/default%3D%23bfffbf/K03868/K10609/K10610/K10140/K10838/K10839/K10570/K10841/K10842/K06634/K10843/K10844/K03141/K03142/K03143/K03144/K10846/K10847/K07466/K10739/K10740/K10848/K10849/K02328/K03505/K02324/K02325/K02326/K04802/K10754/K10755/K10756/K10747) | 33 |
| 131 | 03430 | [Mismatch repair](http://www.genome.jp/kegg-bin/mark_pathway_www?@ko03430/reference%3Dwhite/default%3D%23bfffbf/K03111/K10858/K08734/K08737/K08735/K08736/K08739/K10754/K10755/K10756/K04802/K07466/K10739/K10740/K02328/K03505/K10747) | 17 |
| 132 | 03440 | [Homologous recombination](http://www.genome.jp/kegg-bin/mark_pathway_www?@ko03440/reference%3Dwhite/default%3D%23bfffbf/K03111/K10866/K10865/K07466/K10739/K10740/K04482/K10873/K08775/K10879/K10880/K02328/K03505/K10901/K03165/K10882) | 16 |
| 133 | 03450 | [Non-homologous end-joining](http://www.genome.jp/kegg-bin/mark_pathway_www?@ko03450/reference%3Dwhite/default%3D%23bfffbf/K10884/K10885/K06642/K03512/K10886/K10980/K10866/K10865/K04799) | 9 |
| 134 | 04010 | [MAPK signaling pathway](http://www.genome.jp/kegg-bin/mark_pathway_www?@ko04010/reference%3Dwhite/default%3D%23bfffbf/K05315/K04859/K04345/K02677/K04346/K04347/K04348/K06268/K12326/K04350/K12361/K08018/K08052/K04352/K04353/K02582/K04358/K04359/K03176/K04361/K04362/K05093/K05089/K04364/K03099/K07827/K07830/K07831/K04366/K04368/K04369/K04370/K04371/K04372/K04373/K04374/K04375/K04378/K04379/K04380/K04381/K01047/K13376/K03158/K04386/K04387/K04674/K04388/K04392/K04393/K02187/K02308/K03175/K04402/K04403/K04404/K04405/K04406/K04407/K04408/K04409/K04410/K04415/K04419/K04420/K04424/K04426/K04427/K04428/K04429/K04430/K04431/K04436/K04437/K04438/K04439/K04440/K04441/K04442/K04443/K04444/K04445/K04448/K04449/K04450/K04453/K04454/K04455/K04456/K04457/K04459/K04460/K04461/K03283/K04466/K04467/K04468/K02580/K04469/K04735/K06855) | 101 |
| 135 | 04011 | [MAPK signaling pathway - yeast](http://www.genome.jp/kegg-bin/mark_pathway_www?@ko04011/reference%3Dwhite/default%3D%23bfffbf/K04393/K04409/K02677/K09291/K04441/K01759) | 6 |
| 136 | 04012 | [ErbB signaling pathway](http://www.genome.jp/kegg-bin/mark_pathway_www?@ko04012/reference%3Dwhite/default%3D%23bfffbf/K04361/K05859/K04515/K02677/K04707/K05725/K04438/K07365/K04409/K04410/K05734/K04430/K04431/K04440/K04448/K04375/K08523/K05085/K06279/K04364/K03099/K07827/K08845/K04366/K04368/K04369/K04371/K09593/K11224/K00922/K02649/K04456/K07203/K07205/K02158/K03083/K06625) | 37 |
| 137 | 04013 | [MAPK signaling pathway - fly](http://www.genome.jp/kegg-bin/mark_pathway_www?@ko04013/reference%3Dwhite/default%3D%23bfffbf/K04364/K03099/K07827/K04368/K04371/K07293/K04361/K12380/K02678/K03211) | 10 |
| 138 | 04020 | [Calcium signaling pathway](http://www.genome.jp/kegg-bin/mark_pathway_www?@ko04020/reference%3Dwhite/default%3D%23bfffbf/K05849/K05850/K04131/K04266/K04267/K04141/K04142/K04632/K08042/K08043/K08047/K08049/K04345/K05853/K04964/K04809/K05217/K05219/K04198/K03914/K04158/K04279/K04264/K04361/K05085/K05089/K04634/K05857/K05858/K05859/K05860/K04958/K04959/K04960/K01242/K04718/K05862/K05863/K02183/K07190/K00907/K04515/K05869/K04348/K06268/K13755/K00911/K02677) | 48 |
| 139 | 04060 | [Cytokine-cytokine receptor interaction](http://www.genome.jp/kegg-bin/mark_pathway_www?@ko04060/reference%3Dwhite/default%3D%23bfffbf/K10033/K04359/K05448/K05449/K13376/K04667/K04665/K04662/K04663/K05050/K04175/K04184/K05055/K05056/K05058/K05061/K05062/K04738/K05079/K05081/K05089/K05098/K05097/K04361/K05090/K05092/K05136/K04722/K02583/K03158/K05152/K05143/K05146/K04388/K04674/K04670/K04675/K13567/K04671/K05164/K04386/K04387) | 42 |
| 140 | 04062 | [Chemokine signaling pathway](http://www.genome.jp/kegg-bin/mark_pathway_www?@ko04062/reference%3Dwhite/default%3D%23bfffbf/K10033/K05050/K04175/K04184/K04447/K11220/K04692/K11224/K04630/K08042/K08043/K08046/K08047/K08049/K04345/K05854/K06279/K04364/K03099/K07827/K04366/K04368/K04371/K02649/K00922/K04456/K09408/K04467/K02580/K04735/K03083/K07363/K05730/K04392/K04409/K04393/K05747/K04513/K04514/K04536/K04537/K07826/K04542/K04347/K04547/K12365/K12367/K05725/K05760/K04438/K05858/K12361/K04353/K05731/K00910/K08291/K04439) | 57 |
| 141 | 04070 | [Phosphatidylinositol signaling system](http://www.genome.jp/kegg-bin/mark_pathway_www?@ko04070/reference%3Dwhite/default%3D%23bfffbf/K00888/K13711/K00889/K00901/K00911/K00914/K00920/K00921/K00922/K00923/K10572/K00981/K00999/K01092/K01099/K01110/K01106/K01107/K01109/K05858/K05857/K05860/K05859/K04958/K04959/K04960/K02649/K02677/K03084/K02183) | 30 |
| 142 | 04080 | [Neuroactive ligand-receptor interaction](http://www.genome.jp/kegg-bin/mark_pathway_www?@ko04080/reference%3Dwhite/default%3D%23bfffbf/K04131/K04138/K04140/K04141/K04142/K04158/K04010/K04198/K05236/K01312/K03914/K04234/K04259/K04261/K04263/K04264/K04265/K04266/K04267/K04270/K04272/K04273/K04279/K04289/K04290/K04291/K04292/K04296/K04309/K04577/K04585/K04615/K04616/K05175/K04809/K05217/K05219/K05199/K05207/K05222/K05770/K05771/K05547/K08362/K05062/K05081) | 46 |
| 143 | 04110 | [Cell cycle](http://www.genome.jp/kegg-bin/mark_pathway_www?@ko04110/reference%3Dwhite/default%3D%23bfffbf/K04503/K10151/K02089/K02091/K04681/K06067/K06620/K04683/K03083/K13376/K04500/K04501/K10500/K06623/K06625/K06626/K02206/K03094/K03868/K03875/K06627/K06628/K02214/K06629/K02087/K05868/K06630/K06631/K06632/K06634/K03348/K03350/K03352/K03353/K03354/K03355/K03357/K03358/K12456/K06636/K06669/K06671/K06670/K02178/K02180/K06637/K06638/K02537/K13728/K03364/K06639/K06640/K04728/K02216/K06641/K04498/K06642/K06643/K04402/K04802/K06645/K02603/K02604/K02606/K02607/K02540/K02209/K02542/K02210) | 69 |
| 144 | 04111 | [Cell cycle - yeast](http://www.genome.jp/kegg-bin/mark_pathway_www?@ko04111/reference%3Dwhite/default%3D%23bfffbf/K02603/K02604/K02606/K02607/K02540/K02209/K02542/K02210/K02214/K06628/K03348/K03350/K03352/K03353/K03354/K03355/K03357/K03358/K03094/K03868/K02537/K02178/K02180/K02216/K04354/K04382/K03456/K06685/K06639/K03364/K02219/K02516/K06662/K06641/K06636/K06669/K06670/K06671/K06672/K06674/K06675/K06676/K06677/K06678) | 44 |
| 145 | 04112 | [Cell cycle - Caulobacter](http://www.genome.jp/kegg-bin/mark_pathway_www?@ko04112/reference%3Dwhite/default%3D%23bfffbf/K03544/K01358) | 2 |
| 146 | 04113 | [Meiosis - yeast](http://www.genome.jp/kegg-bin/mark_pathway_www?@ko04113/reference%3Dwhite/default%3D%23bfffbf/K04345/K02603/K02604/K02606/K02607/K02540/K02209/K02542/K02210/K06628/K02214/K06639/K06269/K06662/K02216/K06636/K06669/K06671/K02537/K03348/K03350/K03352/K03353/K03354/K03355/K03357/K03358/K02178/K11584/K04382/K03456) | 31 |
| 147 | 04114 | [Oocyte meiosis](http://www.genome.jp/kegg-bin/mark_pathway_www?@ko04114/reference%3Dwhite/default%3D%23bfffbf/K05459/K08556/K08042/K08043/K08046/K08047/K08049/K04345/K04368/K04371/K04373/K06630/K02087/K02206/K06269/K08836/K06631/K03868/K03094/K03362/K03348/K03350/K03352/K03353/K03354/K03355/K03357/K03358/K12456/K06636/K06669/K02178/K02537/K13728/K06626/K11584/K03456/K04382/K04958/K04959/K04960/K02183/K04348/K06268/K04515) | 45 |
| 148 | 04115 | [p53 signaling pathway](http://www.genome.jp/kegg-bin/mark_pathway_www?@ko04115/reference%3Dwhite/default%3D%23bfffbf/K04728/K06641/K06640/K02216/K06643/K10127/K06625/K04503/K10151/K02089/K02091/K06626/K02206/K05868/K02087/K04402/K10130/K04398/K02159/K10134/K10136/K04506/K08738/K02084/K02187/K05459/K10140/K10808/K10141/K01110/K07207/K10143/K10144/K10146/K10147) | 35 |
| 149 | 04120 | [Ubiquitin mediated proteolysis](http://www.genome.jp/kegg-bin/mark_pathway_www?@ko04120/reference%3Dwhite/default%3D%23bfffbf/K10684/K10685/K10686/K10699/K10573/K06688/K06689/K10687/K10575/K04555/K10576/K10577/K10578/K04554/K04552/K10579/K10580/K10581/K10582/K02207/K10583/K10688/K04649/K10586/K10588/K10590/K10591/K10592/K10593/K10594/K10595/K10614/K10615/K10597/K10598/K10599/K10600/K06643/K04707/K04556/K04506/K03175/K10143/K10144/K04725/K04706/K10605/K10608/K03868/K03094/K03362/K03875/K10260/K10291/K03870/K03872/K03873/K03871/K03869/K10456/K10447/K10609/K10610/K10140/K10570/K10571/K10612/K04694/K03358/K03364/K03348/K03350/K03352/K03353/K03354/K03355/K03357/K12456) | 78 |
| 150 | 04130 | [SNARE interactions in vesicular transport](http://www.genome.jp/kegg-bin/mark_pathway_www?@ko04130/reference%3Dwhite/default%3D%23bfffbf/K08486/K13502/K08487/K08490/K08492/K08493/K08495/K08496/K08497/K08498/K08504/K08507/K08508/K08509/K13505/K08512/K08513/K08515/K08516/K08517) | 20 |
| 151 | 04140 | [Regulation of autophagy](http://www.genome.jp/kegg-bin/mark_pathway_www?@ko04140/reference%3Dwhite/default%3D%23bfffbf/K07198/K08269/K08333/K08334/K00914/K08336/K08337/K08339/K08341/K08342/K08343) | 11 |
| 152 | 04142 | [Lysosome](http://www.genome.jp/kegg-bin/mark_pathway_www?@ko04142/reference%3Dwhite/default%3D%23bfffbf/K02154/K02146/K02144/K03662/K02155/K13289/K01363/K01379/K01366/K01371/K01365/K01374/K01368/K08568/K01369/K01279/K01189/K12309/K12316/K01201/K01217/K01205/K01202/K01195/K12373/K01192/K01186/K01135/K01137/K01136/K01565/K01052/K06129/K01158/K12348/K01444/K12382/K12383/K01074/K06528/K13443/K12386/K12301/K12387/K05642/K06546/K12305/K12388/K12389/K12390/K10532/K13444/K08239/K10087/K01125/K06564/K10089/K04644/K04646/K12393/K12394/K12396/K12397/K12398/K12399/K12400/K12401/K12402/K12403/K12404/K04992) | 71 |
| 153 | 04144 | [Endocytosis](http://www.genome.jp/kegg-bin/mark_pathway_www?@ko04144/reference%3Dwhite/default%3D%23bfffbf/K13376/K04674/K04388/K04679/K04500/K06503/K04361/K05098/K05093/K03176/K05085/K05090/K01528/K07941/K00889/K01115/K04644/K04646/K11824/K11826/K11827/K04707/K10591/K06643/K03175/K11981/K12470/K11247/K11248/K12471/K12473/K12474/K12475/K04175/K05050/K03914/K04141/K04142/K00910/K08291/K04439/K04677/K06278/K12959/K04513/K03283/K11839/K11866/K07897/K12182/K04705/K12183/K12184/K12185/K12188/K12190/K12189/K12195/K12194/K12193/K12191/K12192/K12196/K12199/K12197/K12198/K12200/K12476/K12477/K12478/K07887/K07889/K12480/K12481/K12482/K12483/K07904/K07905/K12484/K12485/K07892/K06093/K06069/K04393/K12486/K12487/K12488/K12489/K12490/K12491/K12492/K12493/K12494/K12495) | 94 |
| 154 | 04145 | [Phagosome](http://www.genome.jp/kegg-bin/mark_pathway_www?@ko04145/reference%3Dwhite/default%3D%23bfffbf/K13505/K13813/K05692/K13882/K08492/K08517/K06752/K07887/K07889/K12478/K00914/K06503/K12182/K02145/K02147/K02148/K02149/K02150/K02151/K02153/K02154/K02146/K02144/K02155/K03662/K07897/K10414/K10415/K10416/K07374/K07375/K06528/K00921/K10089/K01365/K01368/K10956/K09481/K07342/K05653/K08057/K08054/K06498/K06461/K03990/K03991/K06487/K05719/K06493/K06588/K04659/K10159/K06560/K06563/K13885/K08009/K08008/K04392/K08010/K08012) | 60 |
| 155 | 04146 | [Peroxisome](http://www.genome.jp/kegg-bin/mark_pathway_www?@ko04146/reference%3Dwhite/default%3D%23bfffbf/K13336/K13337/K05677/K13339/K13340/K13341/K13342/K13343/K13344/K13345/K13346/K06664/K13348/K13350/K13352/K13353/K12261/K00232/K12405/K13237/K12663/K05678/K08746/K01897/K03426/K13355/K11992/K00624/K05940/K00649/K00803/K13356/K13273/K01640/K03781/K11187/K04564/K13299) | 38 |
| 156 | 04150 | [mTOR signaling pathway](http://www.genome.jp/kegg-bin/mark_pathway_www?@ko04150/reference%3Dwhite/default%3D%23bfffbf/K05459/K02649/K00922/K06276/K04456/K07206/K07207/K07208/K08266/K07203/K07204/K08267/K08268/K05448/K05449/K03258/K02991/K07205/K03259/K08269/K04371/K04373/K08270/K08272/K07198) | 25 |
| 157 | 04210 | [Apoptosis](http://www.genome.jp/kegg-bin/mark_pathway_www?@ko04210/reference%3Dwhite/default%3D%23bfffbf/K04722/K03158/K04386/K02373/K03171/K04400/K04398/K02187/K04397/K04396/K04725/K02161/K08738/K02084/K02310/K02311/K04727/K01173/K04728/K02861/K04730/K04733/K04466/K04467/K02580/K04735/K02582/K03176/K04738/K00922/K02649/K04456/K04739/K04345/K02158/K02159/K04348/K06268/K03853) | 39 |
| 158 | 04260 | [Cardiac muscle contraction](http://www.genome.jp/kegg-bin/mark_pathway_www?@ko04260/reference%3Dwhite/default%3D%23bfffbf/K04859/K09290/K10375/K10351/K12041/K00411/K00412/K00415/K00416/K00417/K00420/K02262/K02256/K02261/K02263/K02264/K02265/K02266/K02267/K02268/K02270/K02271/K02272/K01539/K01540/K05742) | 26 |
| 159 | 04270 | [Vascular smooth muscle contraction](http://www.genome.jp/kegg-bin/mark_pathway_www?@ko04270/reference%3Dwhite/default%3D%23bfffbf/K04634/K01047/K02183/K00907/K12751/K12755/K05858/K04958/K04959/K04960/K02677/K06068/K08845/K04366/K04368/K04369/K04371/K12313/K06269/K04346/K04639/K07532/K12330/K04513/K04514/K04266/K04267/K04263/K12333/K04577/K08448/K08449/K04632/K08042/K08043/K08046/K08047/K08049/K04345/K12337/K12319) | 41 |
| 160 | 04310 | [Wnt signaling pathway](http://www.genome.jp/kegg-bin/mark_pathway_www?@ko04310/reference%3Dwhite/default%3D%23bfffbf/K00181/K00444/K02432/K02354/K02376/K03068/K02353/K03097/K03115/K03083/K02105/K02085/K03456/K11584/K04382/K08957/K02157/K02620/K04491/K04493/K04494/K04496/K04497/K04498/K04499/K04500/K04501/K04427/K04468/K04448/K04503/K10151/K04504/K04345/K04506/K04507/K03094/K04508/K03362/K03868/K04510/K04513/K04514/K04392/K04440/K05858/K04515/K04348/K06268/K02677/K04446) | 51 |
| 161 | 04320 | [Dorso-ventral axis formation](http://www.genome.jp/kegg-bin/mark_pathway_www?@ko04320/reference%3Dwhite/default%3D%23bfffbf/K04361/K04364/K03099/K07827/K04368/K04371/K03211/K02215/K02678/K02599) | 10 |
| 162 | 04330 | [Notch signaling pathway](http://www.genome.jp/kegg-bin/mark_pathway_www?@ko04330/reference%3Dwhite/default%3D%23bfffbf/K06051/K06052/K05948/K02599/K06053/K06054/K02353/K06057/K06058/K06059/K04522/K06060/K06170/K06171/K06172/K06061/K04498/K06063/K04496/K04497/K06065/K06066/K06067) | 23 |
| 163 | 04340 | [Hedgehog signaling pathway](http://www.genome.jp/kegg-bin/mark_pathway_www?@ko04340/reference%3Dwhite/default%3D%23bfffbf/K11988/K06225/K06226/K06229/K06230/K00444/K04662/K04663/K06231/K06232/K06234/K04345/K03083/K08957/K08958/K02218/K03362) | 17 |
| 164 | 04350 | [TGF-beta signaling pathway](http://www.genome.jp/kegg-bin/mark_pathway_www?@ko04350/reference%3Dwhite/default%3D%23bfffbf/K04659/K04660/K04662/K04663/K04665/K13376/K04667/K04670/K04671/K04388/K04674/K04675/K04676/K04500/K04501/K04677/K04679/K04680/K04681/K04683/K04498/K03868/K03094/K04371/K04513/K04514/K03456/K04382) | 28 |
| 165 | 04360 | [Axon guidance](http://www.genome.jp/kegg-bin/mark_pathway_www?@ko04360/reference%3Dwhite/default%3D%23bfffbf/K06843/K04392/K07520/K04348/K06268/K04446/K04513/K04514/K05462/K05703/K05463/K05725/K07524/K05103/K05106/K05108/K05113/K04393/K04409/K04410/K05734/K04352/K07827/K04371/K04630/K06838/K07526/K06840/K06550/K03083/K02090/K07527/K05743/K05765/K07531/K06521/K06841/K07532/K06529/K05719/K06572) | 41 |
| 166 | 04370 | [VEGF signaling pathway](http://www.genome.jp/kegg-bin/mark_pathway_www?@ko04370/reference%3Dwhite/default%3D%23bfffbf/K05098/K05859/K02677/K04718/K07827/K04366/K04368/K04369/K04371/K01047/K04348/K06268/K04446/K11987/K05725/K05760/K04393/K04441/K04443/K04444/K04455/K00922/K02649/K04392/K04456/K02158) | 26 |
| 167 | 04510 | [Focal adhesion](http://www.genome.jp/kegg-bin/mark_pathway_www?@ko04510/reference%3Dwhite/default%3D%23bfffbf/K06236/K06237/K06238/K06242/K05635/K04659/K05717/K06251/K03900/K06476/K06483/K06584/K06585/K06487/K05719/K06493/K06588/K06590/K06591/K04359/K05459/K05448/K05449/K05089/K05098/K04361/K05097/K05732/K13709/K04513/K04514/K10351/K12755/K06269/K00907/K05692/K03853/K05699/K06271/K04437/K05760/K06272/K06274/K06275/K06276/K04456/K03083/K02105/K02677/K05725/K00922/K02649/K01110/K05730/K04392/K04409/K04410/K05734/K04393/K04438/K06277/K04353/K04440/K04448/K06278/K12959/K05703/K06279/K04364/K03099/K04366/K04368/K04371/K04375/K04503/K10151/K04725/K02158/K02161) | 79 |
| 168 | 04512 | [ECM-receptor interaction](http://www.genome.jp/kegg-bin/mark_pathway_www?@ko04512/reference%3Dwhite/default%3D%23bfffbf/K06236/K06237/K06238/K06242/K05635/K04659/K05717/K06251/K03900/K06476/K06483/K06584/K06585/K06487/K05719/K06493/K06588/K06590/K06591/K06262/K06263) | 21 |
| 169 | 04514 | [Cell adhesion molecules (CAMs)](http://www.genome.jp/kegg-bin/mark_pathway_www?@ko04514/reference%3Dwhite/default%3D%23bfffbf/K06752/K06746/K06547/K06567/K06467/K06478/K06087/K06088/K06735/K06785/K06533/K06471/K06461/K06496/K06483/K05719/K06585/K06590/K06816/K06531/K06592/K06736/K06491/K06550/K06781/K06487/K06591/K06584/K07377/K05693/K06760/K07380/K06770/K06797) | 34 |
| 170 | 04520 | [Adherens junction](http://www.genome.jp/kegg-bin/mark_pathway_www?@ko04520/reference%3Dwhite/default%3D%23bfffbf/K06531/K06592/K06082/K04393/K04392/K05747/K05748/K05702/K06084/K06085/K06086/K05699/K05701/K05690/K02105/K05691/K05692/K04513/K05693/K05696/K05697/K03097/K03115/K02620/K04491/K04527/K04361/K04362/K05703/K05705/K04371/K05706/K04674/K04388/K04500/K04501/K04498/K04427/K04468) | 39 |
| 171 | 04530 | [Tight junction](http://www.genome.jp/kegg-bin/mark_pathway_www?@ko04530/reference%3Dwhite/default%3D%23bfffbf/K06087/K06088/K06735/K06785/K06091/K06093/K06069/K06094/K04393/K04382/K03456/K04354/K02677/K06068/K03097/K03115/K04630/K06095/K05701/K06098/K04424/K02105/K06100/K06101/K02089/K05692/K10351/K12755/K10352/K04513/K05702/K07827/K07830/K07831/K06103/K06104/K06105/K06106/K05691/K06107/K05699/K05705/K06109/K06111/K05631/K05629/K06112/K01110/K04456) | 49 |
| 172 | 04540 | [Gap junction](http://www.genome.jp/kegg-bin/mark_pathway_www?@ko04540/reference%3Dwhite/default%3D%23bfffbf/K07372/K04289/K04630/K04359/K05089/K04361/K04364/K03099/K07827/K04366/K04368/K04369/K04371/K04420/K07374/K07375/K02087/K05701/K04141/K04632/K08042/K08043/K08046/K08047/K08049/K04345/K04158/K04634/K05858/K04958/K04959/K04960/K02677/K12319/K07376) | 35 |
| 173 | 04610 | [Complement and coagulation cascades](http://www.genome.jp/kegg-bin/mark_pathway_www?@ko04610/reference%3Dwhite/default%3D%23bfffbf/K03899/K03900/K01314/K03904/K03917/K03907/K03908/K01343/K01348/K03909/K03914/K01331/K01332/K03989/K03990/K03991/K03992/K03996/K04004/K04010) | 20 |
| 174 | 04612 | [Antigen processing and presentation](http://www.genome.jp/kegg-bin/mark_pathway_www?@ko04612/reference%3Dwhite/default%3D%23bfffbf/K06698/K03283/K09489/K04079/K08054/K08056/K08057/K05653/K08059/K01369/K01363/K06752/K01365/K01368/K08060/K08061/K08062/K08063/K05870/K08064/K08065/K08066) | 22 |
| 175 | 04614 | [Renin-angiotensin system](http://www.genome.jp/kegg-bin/mark_pathway_www?@ko04614/reference%3Dwhite/default%3D%23bfffbf/K01283/K13289/K01389/K01393/K01257) | 5 |
| 176 | 04620 | [Toll-like receptor signaling pathway](http://www.genome.jp/kegg-bin/mark_pathway_www?@ko04620/reference%3Dwhite/default%3D%23bfffbf/K10159/K05401/K10168/K05404/K01371/K04392/K00922/K02649/K04456/K05402/K05403/K02373/K04398/K04733/K04730/K03175/K04403/K04404/K04427/K04467/K02580/K04735/K04415/K04368/K04369/K04371/K04430/K04431/K04441/K04440/K04448/K04379/K02861/K09446/K07211/K05410/K11220) | 37 |
| 177 | 04621 | [NOD-like receptor signaling pathway](http://www.genome.jp/kegg-bin/mark_pathway_www?@ko04621/reference%3Dwhite/default%3D%23bfffbf/K08727/K10165/K12792/K03175/K04467/K02580/K04735/K04427/K04403/K04404/K04371/K04440/K04441/K04398/K12795/K12796/K04725/K11859/K12798/K01370/K12803/K12804/K04079/K09487/K12806) | 25 |
| 178 | 04622 | [RIG-I-like receptor signaling pathway](http://www.genome.jp/kegg-bin/mark_pathway_www?@ko04622/reference%3Dwhite/default%3D%23bfffbf/K12647/K05410/K07211/K03171/K02373/K02861/K04398/K04400/K04467/K02580/K04735/K04427/K03175/K04440/K04441/K10652/K08601/K08339/K08336/K12653/K12656/K11594/K09578) | 23 |
| 179 | 04623 | [Cytosolic DNA-sensing pathway](http://www.genome.jp/kegg-bin/mark_pathway_www?@ko04623/reference%3Dwhite/default%3D%23bfffbf/K03018/K03021/K03023/K03026/K03027/K03019/K03020/K03022/K03024/K03025/K02580/K04735/K05410/K07211/K02861/K04467/K01370/K12968) | 18 |
| 180 | 04626 | [Plant-pathogen interaction](http://www.genome.jp/kegg-bin/mark_pathway_www?@ko04626/reference%3Dwhite/default%3D%23bfffbf/K02183/K04368/K00864/K02358/K12795/K09487/K04079) | 7 |
| 181 | 04630 | [Jak-STAT signaling pathway](http://www.genome.jp/kegg-bin/mark_pathway_www?@ko04630/reference%3Dwhite/default%3D%23bfffbf/K04738/K05055/K05056/K05058/K05061/K05062/K05079/K05081/K05136/K11217/K04447/K11219/K11220/K04692/K11222/K11224/K04693/K04498/K04694/K04695/K04698/K04503/K10151/K04703/K04704/K04705/K04706/K04707/K07293/K04364/K03099/K00922/K02649/K04456/K05697) | 35 |
| 182 | 04640 | [Hematopoietic cell lineage](http://www.genome.jp/kegg-bin/mark_pathway_www?@ko04640/reference%3Dwhite/default%3D%23bfffbf/K05092/K06503/K01389/K06467/K05055/K06498/K05090/K06461/K04386/K04387/K05061/K05079/K05056/K06493/K06476/K06263/K06262/K06483) | 18 |
| 183 | 04650 | [Natural killer cell mediated cytotoxicity](http://www.genome.jp/kegg-bin/mark_pathway_www?@ko04650/reference%3Dwhite/default%3D%23bfffbf/K05697/K07293/K05730/K04392/K04409/K04368/K04369/K04371/K05856/K07983/K05855/K07361/K05859/K07984/K00922/K02649/K05703/K06279/K04364/K03099/K07827/K08845/K04366/K04348/K06268/K04446/K02677/K04722/K01353/K07818/K02187) | 31 |
| 184 | 04660 | [T cell receptor signaling pathway](http://www.genome.jp/kegg-bin/mark_pathway_www?@ko04660/reference%3Dwhite/default%3D%23bfffbf/K06478/K05856/K05703/K07361/K07363/K07364/K07365/K05730/K07366/K04364/K04409/K04410/K05734/K04513/K04393/K12076/K04441/K04348/K06268/K04446/K03099/K04350/K07827/K04366/K04368/K04369/K04371/K04379/K04448/K07367/K07368/K07369/K04427/K04431/K04467/K02580/K04735/K02649/K00922/K04456/K04415/K04466/K03083/K05697/K04707/K02089) | 46 |
| 185 | 04662 | [B cell receptor signaling pathway](http://www.genome.jp/kegg-bin/mark_pathway_www?@ko04662/reference%3Dwhite/default%3D%23bfffbf/K05854/K05855/K07370/K12229/K05730/K04392/K04348/K06268/K04446/K04364/K03099/K07827/K04366/K04368/K04369/K04371/K04379/K04448/K07367/K07368/K07369/K04467/K02580/K04735/K06508/K02649/K00922/K04456/K03083/K03084/K06467/K05697/K12230) | 33 |
| 186 | 04664 | [Fc epsilon RI signaling pathway](http://www.genome.jp/kegg-bin/mark_pathway_www?@ko04664/reference%3Dwhite/default%3D%23bfffbf/K07983/K05855/K05854/K07370/K03084/K05859/K00922/K02649/K04456/K04392/K04430/K04431/K04440/K04441/K07361/K05730/K05703/K04364/K03099/K07827/K04366/K04368/K04369/K04371/K01047) | 25 |
| 187 | 04666 | [Fc gamma R-mediated phagocytosis](http://www.genome.jp/kegg-bin/mark_pathway_www?@ko04666/reference%3Dwhite/default%3D%23bfffbf/K06498/K06478/K05854/K05855/K00922/K02649/K04456/K05859/K04366/K04368/K04371/K12561/K01115/K01080/K04718/K02677/K05768/K05730/K04393/K05747/K06274/K05754/K05755/K05756/K05757/K05758/K04392/K05748/K04409/K05743/K05765/K00889/K07941/K04438/K12367/K12488/K03084/K01528/K12559) | 39 |
| 188 | 04670 | [Leukocyte transendothelial migration](http://www.genome.jp/kegg-bin/mark_pathway_www?@ko04670/reference%3Dwhite/default%3D%23bfffbf/K06785/K06461/K06735/K06483/K05719/K06471/K06533/K06087/K06088/K08007/K05763/K05692/K00922/K02649/K04392/K08008/K08009/K08010/K08012/K02105/K05690/K05691/K07293/K04441/K05859/K02677/K05725/K05760/K05732/K13709/K04513/K04514/K10351/K12755/K05702/K04353/K08013/K06274/K05699/K04630/K04351/K07363/K05730/K04393/K07873) | 45 |
| 189 | 04672 | [Intestinal immune network for IgA production](http://www.genome.jp/kegg-bin/mark_pathway_www?@ko04672/reference%3Dwhite/default%3D%23bfffbf/K06752/K04184/K06483/K06590/K04466) | 5 |
| 190 | 04710 | [Circadian rhythm - mammal](http://www.genome.jp/kegg-bin/mark_pathway_www?@ko04710/reference%3Dwhite/default%3D%23bfffbf/K02633/K02295/K02296/K02223/K08532/K08533/K03729/K03730/K03868/K03094/K03362/K10269) | 12 |
| 191 | 04711 | [Circadian rhythm - fly](http://www.genome.jp/kegg-bin/mark_pathway_www?@ko04711/reference%3Dwhite/default%3D%23bfffbf/K02218/K02633/K02223/K02296/K09057/K03083) | 6 |
| 192 | 04712 | [Circadian rhythm - plant](http://www.genome.jp/kegg-bin/mark_pathway_www?@ko04712/reference%3Dwhite/default%3D%23bfffbf/K03097/K03115) | 2 |
| 193 | 04720 | [Long-term potentiation](http://www.genome.jp/kegg-bin/mark_pathway_www?@ko04720/reference%3Dwhite/default%3D%23bfffbf/K04345/K06269/K04515/K04353/K04348/K06268/K02183/K04498/K04374/K05869/K07827/K08845/K04366/K04368/K04369/K04371/K04373/K04958/K04959/K04960/K02677/K04634/K05858) | 23 |
| 194 | 04722 | [Neurotrophin signaling pathway](http://www.genome.jp/kegg-bin/mark_pathway_www?@ko04722/reference%3Dwhite/default%3D%23bfffbf/K02582/K03176/K05101/K07193/K04364/K03099/K07827/K04366/K04368/K04369/K04371/K04373/K04445/K04374/K02161/K04438/K06277/K04353/K04441/K04443/K06279/K09593/K02649/K00922/K04456/K02580/K04735/K09408/K02158/K03083/K07187/K05859/K02183/K04515/K05869/K07293/K02583/K04513/K04393/K04392/K04426/K04431/K04440/K04448/K02159/K03175/K12463/K06630/K04730/K04733/K12388) | 51 |
| 195 | 04730 | [Long-term depression](http://www.genome.jp/kegg-bin/mark_pathway_www?@ko04730/reference%3Dwhite/default%3D%23bfffbf/K12319/K07376/K03456/K04382/K07827/K08845/K04366/K04368/K04369/K04371/K05207/K04630/K04534/K04632/K04346/K04639/K01047/K02677/K04634/K05858/K05199/K05854/K04958/K04959/K04960/K05459) | 26 |
| 196 | 04740 | [Olfactory transduction](http://www.genome.jp/kegg-bin/mark_pathway_www?@ko04740/reference%3Dwhite/default%3D%23bfffbf/K04345/K07376/K08043/K04515/K02183) | 5 |
| 197 | 04742 | [Taste transduction](http://www.genome.jp/kegg-bin/mark_pathway_www?@ko04742/reference%3Dwhite/default%3D%23bfffbf/K04536/K04547/K04960/K04632/K08046/K04345/K04885) | 7 |
| 198 | 04744 | [Phototransduction](http://www.genome.jp/kegg-bin/mark_pathway_www?@ko04744/reference%3Dwhite/default%3D%23bfffbf/K04439/K04536/K13765/K02183) | 4 |
| 199 | 04745 | [Phototransduction - fly](http://www.genome.jp/kegg-bin/mark_pathway_www?@ko04745/reference%3Dwhite/default%3D%23bfffbf/K04634/K04547/K05858/K02677/K02183/K05692/K00910/K04515/K13806) | 9 |
| 200 | 04810 | [Regulation of actin cytoskeleton](http://www.genome.jp/kegg-bin/mark_pathway_www?@ko04810/reference%3Dwhite/default%3D%23bfffbf/K03914/K04358/K04359/K04361/K04362/K05093/K05089/K05717/K06476/K06483/K06584/K06585/K06487/K06461/K06462/K06594/K05719/K06493/K06588/K06590/K06591/K04131/K04346/K04639/K04347/K05725/K04438/K03099/K07827/K07830/K07831/K00922/K02649/K05730/K05731/K08845/K04366/K04368/K04369/K04371/K12330/K07532/K05732/K04513/K04392/K04393/K04409/K04410/K05734/K04514/K00907/K06269/K10351/K12755/K05741/K05742/K00889/K00920/K00921/K05743/K05747/K05748/K05749/K05750/K05751/K05752/K05754/K05755/K05756/K05757/K05758/K05692/K05759/K05760/K08007/K05762/K05763/K05765/K05766/K05767/K05768/K05699/K02085) | 83 |
| 201 | 04910 | [Insulin signaling pathway](http://www.genome.jp/kegg-bin/mark_pathway_www?@ko04910/reference%3Dwhite/default%3D%23bfffbf/K04527/K07187/K02649/K00922/K06276/K04456/K03083/K00693/K06269/K07189/K07190/K02183/K00688/K13296/K04345/K04739/K06069/K07192/K07193/K06086/K04707/K04438/K06277/K07194/K07195/K07197/K11262/K00665/K12406/K00844/K07198/K07199/K07200/K07201/K03841/K01596/K07203/K07204/K02991/K07205/K03259/K07206/K07207/K07208/K02158/K06279/K04364/K03099/K07827/K08845/K04366/K04368/K04369/K04371/K04372/K04375/K04694/K04695/K05696/K04440/K03084) | 61 |
| 202 | 04912 | [GnRH signaling pathway](http://www.genome.jp/kegg-bin/mark_pathway_www?@ko04912/reference%3Dwhite/default%3D%23bfffbf/K04632/K08042/K08043/K08046/K08047/K08049/K04345/K04374/K04634/K05858/K04958/K04959/K04960/K02183/K04515/K04420/K04428/K04441/K01047/K01115/K04393/K04430/K04431/K04440/K04448/K07763/K08523/K04361/K04364/K03099/K07827/K04366/K04368/K04369/K04371/K04375) | 36 |
| 203 | 04914 | [Progesterone-mediated oocyte maturation](http://www.genome.jp/kegg-bin/mark_pathway_www?@ko04914/reference%3Dwhite/default%3D%23bfffbf/K08556/K02649/K00922/K04440/K04441/K04630/K08042/K08043/K08046/K08047/K08049/K04345/K02206/K02087/K06627/K04079/K04368/K04371/K04373/K05868/K06631/K06645/K05459/K04456/K13296/K07827/K08845/K04366/K02178/K06638/K02537/K13728/K03364/K03348/K03350/K03352/K03353/K03354/K03355/K03357/K03358/K12456) | 42 |
| 204 | 04916 | [Melanogenesis](http://www.genome.jp/kegg-bin/mark_pathway_www?@ko04916/reference%3Dwhite/default%3D%23bfffbf/K04632/K08042/K08043/K08046/K08047/K08049/K04345/K05870/K09048/K04498/K09455/K00444/K02432/K02354/K02376/K04534/K04634/K02353/K03083/K02105/K02620/K04491/K07827/K04366/K04368/K04369/K04371/K00506/K04198/K04630/K05858/K02183/K04515/K02677) | 34 |
| 205 | 04920 | [Adipocytokine signaling pathway](http://www.genome.jp/kegg-bin/mark_pathway_www?@ko04920/reference%3Dwhite/default%3D%23bfffbf/K03158/K03171/K07203/K04440/K04467/K02580/K04735/K07187/K04456/K01897/K05062/K04447/K04692/K07198/K07199/K07200/K01596/K07293/K08524/K08525/K07297/K07359/K08765/K07299) | 24 |
| 206 | 04930 | [Type II diabetes mellitus](http://www.genome.jp/kegg-bin/mark_pathway_www?@ko04930/reference%3Dwhite/default%3D%23bfffbf/K04527/K07187/K02649/K00922/K04371/K07203/K04694/K04695/K04440/K00844/K00873/K12406) | 12 |
| 207 | 04940 | [Type I diabetes mellitus](http://www.genome.jp/kegg-bin/mark_pathway_www?@ko04940/reference%3Dwhite/default%3D%23bfffbf/K01580/K04077/K06752/K07818/K01353) | 5 |
| 208 | 04950 | [Maturity onset diabetes of the young](http://www.genome.jp/kegg-bin/mark_pathway_www?@ko04950/reference%3Dwhite/default%3D%23bfffbf/K08028/K06054/K12406) | 3 |
| 209 | 04960 | [Aldosterone-regulated sodium reabsorption](http://www.genome.jp/kegg-bin/mark_pathway_www?@ko04960/reference%3Dwhite/default%3D%23bfffbf/K13302/K07827/K01539/K01540/K05459/K04527/K07187/K02649/K00922/K06276/K02677/K04371) | 12 |
| 210 | 04962 | [Vasopressin-regulated water reabsorption](http://www.genome.jp/kegg-bin/mark_pathway_www?@ko04962/reference%3Dwhite/default%3D%23bfffbf/K04632/K08046/K08043/K08049/K04345/K05870/K09048/K07904/K07905/K10414/K10415/K10416/K10418/K10424/K10426/K10427/K10428/K06027/K13502/K07887/K07889/K09866/K09876) | 23 |
| 211 | 04964 | [Proximal tubule bicarbonate reclamation](http://www.genome.jp/kegg-bin/mark_pathway_www?@ko04964/reference%3Dwhite/default%3D%23bfffbf/K09864/K01539/K01540/K13576/K01425/K00261/K13577/K00025/K01596) | 9 |
| 212 | 04966 | [Collecting duct acid secretion](http://www.genome.jp/kegg-bin/mark_pathway_www?@ko04966/reference%3Dwhite/default%3D%23bfffbf/K02145/K02147/K02148/K02149/K02150/K02151/K02153/K02154/K02155/K02146/K06573/K13627) | 12 |
| 213 | 04970 | [Salivary secretion](http://www.genome.jp/kegg-bin/mark_pathway_www?@ko04970/reference%3Dwhite/default%3D%23bfffbf/K04141/K04142/K04632/K08042/K08043/K08046/K08047/K08049/K04345/K04131/K04634/K05858/K02677/K04958/K04959/K04960/K02183/K12319/K07376/K01242/K01539/K01540/K05850/K10951/K04945/K13855/K05742) | 27 |
| 214 | 05010 | [Alzheimer's disease](http://www.genome.jp/kegg-bin/mark_pathway_www?@ko05010/reference%3Dwhite/default%3D%23bfffbf/K06704/K06059/K04520/K04532/K04529/K00134/K04521/K06170/K04522/K06171/K06172/K01389/K03942/K03945/K03948/K03949/K03950/K03951/K03952/K03953/K03954/K03955/K03958/K03959/K03960/K03961/K03962/K03963/K03964/K03965/K03966/K03934/K03935/K03936/K03937/K03938/K03939/K03941/K03968/K00234/K00235/K00236/K00237/K00411/K00412/K00415/K00416/K00417/K00420/K02262/K02256/K02261/K02263/K02264/K02265/K02266/K02267/K02268/K02270/K02271/K02272/K02136/K02134/K02135/K02126/K02127/K02128/K02131/K08683/K01059/K03158/K02373/K04398/K02183/K04348/K06268/K02158/K08738/K02084/K02187/K04634/K05858/K04371/K04958/K04959/K04960/K05853/K09054/K08852/K03853/K02090/K04380/K03083/K04397) | 94 |
| 215 | 05012 | [Parkinson's disease](http://www.genome.jp/kegg-bin/mark_pathway_www?@ko05012/reference%3Dwhite/default%3D%23bfffbf/K04552/K04554/K10578/K04555/K10575/K04556/K04557/K04558/K03878/K03879/K03883/K03942/K03945/K03948/K03949/K03950/K03951/K03952/K03953/K03954/K03955/K03958/K03959/K03960/K03961/K03962/K03963/K03964/K03965/K03966/K03934/K03935/K03936/K03937/K03938/K03939/K03941/K03968/K00234/K00235/K00236/K00237/K00411/K00412/K00415/K00416/K00417/K00420/K02262/K02256/K02261/K02263/K02264/K02265/K02266/K02267/K02268/K02270/K02271/K02272/K02136/K02134/K02135/K02126/K02127/K02128/K02131/K05688/K05687/K05862/K05863/K08738/K02084/K02187) | 74 |
| 216 | 05014 | [Amyotrophic lateral sclerosis (ALS)](http://www.genome.jp/kegg-bin/mark_pathway_www?@ko05014/reference%3Dwhite/default%3D%23bfffbf/K04348/K06268/K01370/K02161/K02159/K02158/K02084/K08738/K02187/K11518/K11519/K04426/K03158/K02308/K04441/K04569/K03781/K07887/K04392) | 19 |
| 217 | 05016 | [Huntington's disease](http://www.genome.jp/kegg-bin/mark_pathway_www?@ko05016/reference%3Dwhite/default%3D%23bfffbf/K04644/K04646/K04559/K11824/K11826/K11827/K04533/K04638/K04398/K02187/K04634/K05858/K04958/K05625/K06067/K03006/K03010/K03011/K03012/K03013/K03014/K03015/K03016/K03017/K03007/K03008/K03009/K10424/K10426/K10412/K04498/K03129/K03120/K05870/K09048/K08530/K11830/K11831/K04564/K02159/K03942/K03945/K03948/K03949/K03950/K03951/K03952/K03953/K03954/K03955/K03958/K03959/K03960/K03961/K03962/K03963/K03964/K03965/K03966/K03934/K03935/K03936/K03937/K03938/K03939/K03941/K03968/K00234/K00235/K00236/K00237/K00411/K00412/K00415/K00416/K00417/K00420/K02262/K02256/K02261/K02263/K02264/K02265/K02266/K02267/K02268/K02270/K02271/K02272/K02136/K02134/K02135/K02126/K02127/K02128/K02131/K05862/K05863/K08738/K02084) | 100 |
| 218 | 05020 | [Prion diseases](http://www.genome.jp/kegg-bin/mark_pathway_www?@ko05020/reference%3Dwhite/default%3D%23bfffbf/K09490/K06491/K05635/K04345/K02159/K05703/K03996/K04368/K04369/K04371/K04375/K09203) | 12 |
| 219 | 05100 | [Bacterial invasion of epithelial cells](http://www.genome.jp/kegg-bin/mark_pathway_www?@ko05100/reference%3Dwhite/default%3D%23bfffbf/K02105/K05691/K09593/K02649/K00922/K04438/K04393/K04392/K05747/K05748/K05757/K05758/K05756/K05755/K05754/K05692/K04707/K06279/K01528/K04644/K04646/K06278/K12959/K06106/K05725/K05760/K05717/K05719/K06272/K13728/K13744/K07863/K04513) | 33 |
| 220 | 05110 | [Vibrio cholerae infection](http://www.genome.jp/kegg-bin/mark_pathway_www?@ko05110/reference%3Dwhite/default%3D%23bfffbf/K02145/K02147/K02148/K02149/K02150/K02151/K02153/K02154/K02146/K02144/K03662/K02155/K10949/K10956/K09481/K07342/K04632/K08043/K08049/K04345/K10951/K05692/K05859/K02677/K05701/K06098/K10955) | 27 |
| 221 | 05120 | [Epithelial cell signaling in Helicobacter pylori infection](http://www.genome.jp/kegg-bin/mark_pathway_www?@ko05120/reference%3Dwhite/default%3D%23bfffbf/K07293/K05859/K05701/K06735/K06785/K06059/K08523/K04361/K04175/K05050/K06704/K05854/K04392/K04393/K04441/K04409/K04430/K04440/K04448/K04466/K04467/K02580/K04735/K08727/K08114/K02187/K02145/K02147/K02148/K02149/K02150/K02151/K02153/K02154/K02146/K02144/K03662/K02155) | 38 |
| 222 | 05130 | [Pathogenic Escherichia coli infection](http://www.genome.jp/kegg-bin/mark_pathway_www?@ko05130/reference%3Dwhite/default%3D%23bfffbf/K10168/K07374/K07375/K04513/K04514/K07365/K05747/K05757/K05758/K05756/K05755/K05754/K05692/K05703/K06106/K11294/K05719/K04393/K06088/K08007/K02105) | 21 |
| 223 | 05131 | [Shigellosis](http://www.genome.jp/kegg-bin/mark_pathway_www?@ko05131/reference%3Dwhite/default%3D%23bfffbf/K05719/K07863/K04392/K05748/K05754/K05755/K05756/K05757/K05758/K06106/K04438/K04393/K04514/K05747/K05692/K05759/K08339/K08727/K10165/K04440/K04371/K04441/K04467/K02580/K04735/K12836/K03362/K13728) | 28 |
| 224 | 05140 | [Leishmaniasis](http://www.genome.jp/kegg-bin/mark_pathway_www?@ko05140/reference%3Dwhite/default%3D%23bfffbf/K10159/K04730/K04733/K03175/K04427/K04403/K04404/K02580/K04735/K13376/K03990/K06461/K06498/K06483/K05719/K11987/K08010/K08012/K08009/K04371/K04375/K04379/K04448/K04441/K11217/K04447/K11220/K06752/K05697) | 29 |
| 225 | 05142 | [Chagas disease](http://www.genome.jp/kegg-bin/mark_pathway_www?@ko05142/reference%3Dwhite/default%3D%23bfffbf/K10159/K04730/K04733/K03175/K04430/K04440/K04371/K04441/K04379/K04448/K04467/K02580/K04735/K03456/K04354/K04382/K03158/K03990/K08057/K13376/K01283/K04634/K05858/K04630/K04534/K04632/K02649/K00922/K04456/K04388/K04674/K04500/K02373/K04398) | 34 |
| 226 | 05144 | [Malaria](http://www.genome.jp/kegg-bin/mark_pathway_www?@ko05144/reference%3Dwhite/default%3D%23bfffbf/K06508/K10159/K13376/K06471/K04659/K06496) | 6 |
| 227 | 05200 | Pathways in cancer | 138 |
| 228 | 05210 | [Colorectal cancer](http://www.genome.jp/kegg-bin/mark_pathway_www?@ko05210/reference%3Dwhite/default%3D%23bfffbf/K03083/K02157/K02105/K02085/K02620/K04491/K04503/K07827/K00922/K02649/K04456/K02158/K08845/K04366/K04368/K04371/K04448/K04379/K04392/K04513/K04440/K02187/K08733/K13376/K04674/K04388/K04500/K04501/K08734/K08735/K08736/K08737/K02159/K02161/K08738) | 35 |
| 229 | 05211 | [Renal cell carcinoma](http://www.genome.jp/kegg-bin/mark_pathway_www?@ko05211/reference%3Dwhite/default%3D%23bfffbf/K08268/K09095/K09592/K03871/K03872/K03873/K03868/K03870/K09097/K04498/K07299/K05448/K05449/K13376/K09593/K00922/K02649/K04456/K04438/K06277/K04353/K07293/K04364/K03099/K07827/K08845/K04366/K04368/K04369/K04371/K04448/K04392/K04393/K04409/K04410/K05734/K01679/K09594) | 38 |
| 230 | 05212 | [Pancreatic cancer](http://www.genome.jp/kegg-bin/mark_pathway_www?@ko05212/reference%3Dwhite/default%3D%23bfffbf/K07827/K00922/K02649/K04392/K02580/K04735/K04456/K04467/K02158/K08845/K04366/K04368/K04371/K04440/K07834/K07835/K08773/K04393/K04361/K11217/K04692/K11220/K05448/K05449/K02089/K02091/K04503/K06620/K13376/K04674/K04388/K04500/K04501/K08775/K04482) | 35 |
| 231 | 05213 | [Endometrial cancer](http://www.genome.jp/kegg-bin/mark_pathway_www?@ko05213/reference%3Dwhite/default%3D%23bfffbf/K04361/K00922/K02649/K01110/K06276/K06272/K04456/K02158/K09408/K04364/K03099/K07827/K08845/K04366/K04368/K04369/K04371/K04375/K08734/K02105/K05691/K02157/K02085/K03083/K02620/K04491/K04503) | 27 |
| 232 | 05214 | [Glioma](http://www.genome.jp/kegg-bin/mark_pathway_www?@ko05214/reference%3Dwhite/default%3D%23bfffbf/K04361/K04359/K05089/K05459/K05859/K02183/K04515/K02677/K06279/K04364/K03099/K07827/K08845/K04366/K04368/K04369/K04371/K00922/K02649/K04456/K07203/K01110/K06643/K06625/K04503/K02089/K02091/K06620) | 28 |
| 233 | 05215 | [Prostate cancer](http://www.genome.jp/kegg-bin/mark_pathway_www?@ko05215/reference%3Dwhite/default%3D%23bfffbf/K02206/K06626/K06620/K04359/K05459/K05089/K04362/K05093/K04361/K00922/K02649/K01110/K06276/K04456/K02158/K07201/K06625/K06643/K03083/K05870/K04374/K09048/K02105/K04498/K02620/K04491/K04503/K04467/K02580/K04735/K02161/K07203/K04364/K03099/K07827/K08845/K04366/K04368/K04369/K04371/K04079/K09487) | 42 |
| 234 | 05216 | [Thyroid cancer](http://www.genome.jp/kegg-bin/mark_pathway_www?@ko05216/reference%3Dwhite/default%3D%23bfffbf/K09288/K03176/K09290/K09291/K09292/K07827/K04368/K04369/K04371/K08530/K08524/K08525/K02105/K02620/K04491/K04503) | 16 |
| 235 | 05217 | [Basal cell carcinoma](http://www.genome.jp/kegg-bin/mark_pathway_www?@ko05217/reference%3Dwhite/default%3D%23bfffbf/K11988/K06225/K06226/K06229/K06230/K04662/K06231/K00444/K02432/K02354/K02376/K02353/K03083/K02157/K02085/K02105/K02620/K04491) | 18 |
| 236 | 05218 | [Melanoma](http://www.genome.jp/kegg-bin/mark_pathway_www?@ko05218/reference%3Dwhite/default%3D%23bfffbf/K04358/K05459/K04359/K04362/K05089/K04361/K07827/K08845/K04366/K04368/K04369/K04371/K02089/K00922/K02649/K04456/K02158/K01110/K06643/K06625/K04503/K02091/K06620/K09455) | 24 |
| 237 | 05219 | [Bladder cancer](http://www.genome.jp/kegg-bin/mark_pathway_www?@ko05219/reference%3Dwhite/default%3D%23bfffbf/K07827/K08845/K04366/K04368/K04369/K04371/K08803/K06643/K06625/K04503/K02089/K06620/K04361/K05448/K05449) | 15 |
| 238 | 05220 | [Chronic myeloid leukemia](http://www.genome.jp/kegg-bin/mark_pathway_www?@ko05220/reference%3Dwhite/default%3D%23bfffbf/K04438/K04707/K00922/K02649/K04456/K02158/K04467/K02580/K04735/K06643/K04364/K07293/K03099/K07827/K04366/K08845/K04368/K04369/K04371/K06279/K11224/K06625/K04503/K02089/K02091/K06620/K13376/K04674/K04388/K04501/K08367/K04496/K06067) | 33 |
| 239 | 05221 | [Acute myeloid leukemia](http://www.genome.jp/kegg-bin/mark_pathway_www?@ko05221/reference%3Dwhite/default%3D%23bfffbf/K05092/K00922/K02649/K04456/K04467/K02580/K04735/K02158/K07203/K07205/K04364/K03099/K07827/K08845/K04366/K04368/K04369/K04371/K04692/K11224/K08367/K08527/K09055/K02620/K04491/K04503/K04504) | 27 |
| 240 | 05222 | [Small cell lung cancer](http://www.genome.jp/kegg-bin/mark_pathway_www?@ko05222/reference%3Dwhite/default%3D%23bfffbf/K01522/K08524/K08525/K02161/K08738/K02084/K04453/K04706/K02089/K02091/K04503/K02219/K03875/K02206/K06626/K06620/K06237/K06242/K05635/K05717/K06476/K06487/K05719/K05725/K00922/K02649/K01110/K04456/K04467/K02580/K04735/K04725/K03175/K11987) | 34 |
| 241 | 05223 | [Non-small cell lung cancer](http://www.genome.jp/kegg-bin/mark_pathway_www?@ko05223/reference%3Dwhite/default%3D%23bfffbf/K01522/K08524/K08525/K02089/K02091/K04503/K06620/K07827/K00922/K02649/K06276/K04456/K02158/K09408/K04361/K04364/K03099/K08845/K04366/K04368/K04369/K04371/K05859/K02677) | 24 |
| 242 | 05310 | [Asthma](http://www.genome.jp/kegg-bin/mark_pathway_www?@ko05310/reference%3Dwhite/default%3D%23bfffbf/K06752/K07983) | 2 |
| 243 | 05320 | [Autoimmune thyroid disease](http://www.genome.jp/kegg-bin/mark_pathway_www?@ko05320/reference%3Dwhite/default%3D%23bfffbf/K06752/K07818/K01353) | 3 |
| 244 | 05322 | [Systemic lupus erythematosus](http://www.genome.jp/kegg-bin/mark_pathway_www?@ko05322/reference%3Dwhite/default%3D%23bfffbf/K01332/K03989/K11251/K11252/K11253/K11087/K11088/K10651/K11089/K11090/K05699/K06752/K01331/K03990/K03996/K06498) | 16 |
| 245 | 05330 | [Allograft rejection](http://www.genome.jp/kegg-bin/mark_pathway_www?@ko05330/reference%3Dwhite/default%3D%23bfffbf/K07818/K01353/K06752) | 3 |
| 246 | 05332 | [Graft-versus-host disease](http://www.genome.jp/kegg-bin/mark_pathway_www?@ko05332/reference%3Dwhite/default%3D%23bfffbf/K06752/K07818/K01353) | 3 |
| 247 | 05340 | [Primary immunodeficiency](http://www.genome.jp/kegg-bin/mark_pathway_www?@ko05340/reference%3Dwhite/default%3D%23bfffbf/K06478/K05653/K05856/K08061/K08063/K08062/K08060/K07370/K03648) | 9 |
| 248 | 05410 | [Hypertrophic cardiomyopathy (HCM)](http://www.genome.jp/kegg-bin/mark_pathway_www?@ko05410/reference%3Dwhite/default%3D%23bfffbf/K06476/K06483/K06584/K06585/K06487/K05719/K06493/K06588/K06590/K06591/K12566/K07610/K05692/K09290/K10375/K12568/K10351/K12641/K04859/K07198/K07199/K07200/K01283/K05459/K13376) | 25 |
| 249 | 05412 | [Arrhythmogenic right ventricular cardiomyopathy (ARVC)](http://www.genome.jp/kegg-bin/mark_pathway_www?@ko05412/reference%3Dwhite/default%3D%23bfffbf/K06476/K06483/K06584/K06585/K06487/K05719/K06493/K06588/K06590/K06591/K12566/K07610/K05692/K12641/K02105/K02620/K04491/K04859/K06736/K05691/K05699/K10381/K07372) | 23 |
| 250 | 05414 | [Dilated cardiomyopathy (DCM)](http://www.genome.jp/kegg-bin/mark_pathway_www?@ko05414/reference%3Dwhite/default%3D%23bfffbf/K06476/K06483/K06584/K06585/K06487/K05719/K06493/K06588/K06590/K06591/K12566/K07610/K05692/K09290/K10375/K12568/K10351/K12641/K04141/K04632/K08042/K08043/K08046/K08047/K08049/K04345/K04859/K05459/K13376) | 29 |
| 251 | 05416 | [Viral myocarditis](http://www.genome.jp/kegg-bin/mark_pathway_www?@ko05416/reference%3Dwhite/default%3D%23bfffbf/K05703/K06278/K04392/K12566/K05692/K03260/K04503/K04398/K08738/K02187/K10352/K06752/K07818) | 13 |
