# Supplementary material for: De Novo Characterization of the Spleen Transcriptome of the Large Yellow Croaker (Pseudosciaena crocea) and Analysis of the Immune Relevant Genes and Pathways Involved in the Antiviral Response
Source: PLoS One. 2014 May 12;9(5):e97471. doi: 10.1371/journal.pone.0097471 (PMC4018400; doi:10.1371/journal.pone.0097471)
Supplement: Table S4 — Immune-relevant genes. (DOC) [file pone.0097471.s005.doc]

**Table S4. Immune-relevant genes**

| gene | describe | length | identify | P value |
| --- | --- | --- | --- | --- |
| TLR1 | toll-like receptor 1 | 3671 | 52 | 1.00E-62 |
| TLR2 | toll-like receptor 2 | 1321 | 41 | 3.00E-92 |
| TLR3 | toll-like receptor 3 | 3528 | 41 | ------- |
| TLR5b | toll-like receptor 5b | 3033 | 35 | 5.00E-18 |
| TLR9 | toll-like receptor 9 | 3500 | 38 | -------- |
| TLR22 | toll-like receptor 22 | 2023 | 40 | 4.00E-09 |
| Ticam1 | toll-like receptor adaptor molecule 1 | 2117 | 72 | 7.00E-32 |
| IL-1b | interleukin 1, beta | 627 | 57 | 7.00E-11 |
| IL-2 | interleukin-2 | 1827 | 40 | 4.00E-10 |
| IL-6 | interleukin-6 | 1409 | 50 | 1.00E-07 |
| IL-8 | interleukin-8 | 462 | 78 | 1.00E-07 |
| IL-10 | interleukin 10 | 504 | 59 | 3.00E-15 |
| IL-12p35 | interleukin-12 p35 subunit | 806 | 58 | 5.00E-15 |
| IL-12p40 | interleukin-12 p40 chain | 115 | 63 | 1.00E-06 |
| IL-12b | interleukin 12B | 1376 | 32 | 9.00E-09 |
| IL-17d | interleukin 17d | 1055 | 43 | 9.00E-06 |
| IL-1R1 | interleukin 1 receptor-like 1 ligand | 164 | 77 | 1.00E-24 |
| IL-2Rb | interleukin 2 receptor, beta | 838 | 45 | 4.00E-06 |
| IL-2Rga | interleukin 2 receptor, gamma a | 169 | 50 | 1.00E-07 |
| IL-6R | interleukin 6 receptor | 2411 | 40 | 2.00E-14 |
| IL-7R | interleukin 7 receptor | 286 | 38 | 4.00E-09 |
| IL-10Rb | Interleukin-10 receptor beta chain | 156 | 61 | 6.00E-10 |
| IL-12Rb2 | interleukin 12 receptor, beta 2a, like | 652 | 43 | 9.00E-06 |
| IL-17R | interleukin-17 receptor | 123 | 65 | 8.00E-07 |
| IL-17RA | Interleukin-17 receptor A precursor | 199 | 50 | 1.00E-12 |
| IL-21R | interleukin-21 receptor | 1678 | 43 | 7.00E-13 |
| IRAK1 | interleukin 1 receptor-associated kinase 1 | 185 | 59 | 4.00E-14 |
| IRAK4 | interleukin-1 receptor-associated kinase 4 | 599 | 58 | 1.00E-25 |
|  | interleukin-1 receptor activated kinase 1 | 1180 | 62 | 8.00E-47 |
| IL-R1ap1b | interleukin 1 receptor accessory protein-like 1b | 897 | 37 | 7.00E-06 |
| IL-6st | interleukin 6 signal transducer | 214 | 61 | 2.00E-15 |
| ScarB1 | scavenger receptor class B, member 1 | 1662 | 49 | 2.00E-63 |
| ScarB2 | scavenger receptor class B, member 2 | 2260 | 50 | 1.00E-49 |
| ScarA5 | Scavenger receptor class A member 5 | 1165 | 50 | 1.00E-05 |
| ScarF1 | Scavenger receptor class F member 1 | 469 | 51 | 8.00E-24 |
|  | mannose receptor C1-like protein | 169 | 40 | 4.00E-07 |
| Lman2 | lectin, mannose-binding 2 | 2496 | 52 | 1.00E-56 |
| Nod1 | Nod1 protein | 766 | 52 | 6.00E-10 |
| Nod2 | nucleotide-binding oligomerization domain-containing protein 2 | 127 | 80 | 7.00E-09 |
| Nod3 | NOD3 protein | 168 | 61 | 1.00E-10 |
| Illr4 | immune-related, lectin-like receptor 4 | 695 | 42 | 5.00E-14 |
|  | Galectin-related protein | 414 | 31 | 3.00E-13 |
| MLEC | Malectin Precursor | 1351 | 80 |  |
|  | Collectin-12 | 2459 | 36 | 5.00E-09 |
|  | Galectin-5 (RL-18) | 694 | 42 | 8.00E-12 |
|  | erlectin | 2383 | 46 | 4.00E-60 |
|  | skin mucus lectin | 551 | 61 | 7.00E-12 |
|  | putative F-type lectin | 129 | 66 | 3.00E-08 |
|  | C-lectin-A | 201 | 62 | 2.00E-15 |
| Clec4C | C-type lectin domain family 4 member C | 248 | 47 | 4.00E-16 |
| Clec4E | C-type lectin domain family 4 member E | 101 | 68 | 5.00E-07 |
| Clec14A | C-type lectin domain family 14, member A | 1272 | 36 | 3.00E-10 |
| Clec16A | C-type lectin domain family 16, member A | 1602 | 54 | 3.00E-77 |
| Tollip | toll interacting protein | 2525 | 45 | 7.00E-34 |
| LBPa | lipopolysaccharide binding protein variant a | 312 | 51 | 4.00E-25 |
| WDHD1 | WD repeat and HMG-box DNA binding protein 1 | 954 | 76 | 2.00E-27 |
| HBP1 | HMG-box transcription factor 1 | 1460 | 96 | 8.00E-48 |
| Tcf7l1b | Transcription factor 7-like 1-B (HMG box transcription factor 3-B)(TCF-3-B) | 132 | 54 | 8.00E-07 |
| LRRC4 | leucine rich repeat containing 4 | 139 | 73 | 6.00E-13 |
| LRRC8A | leucine rich repeat containing 8, member A | 7736 | 85 | 0 |
| LRRC8D | leucine rich repeat containing 8, member D | 4427 | 47 | 1.00E-174 |
| LRRC15 | leucine rich repeat containing 15 | 1744 | 32 | 3.00E-08 |
| LRRC16 | leucine rich repeat containing 16 | 485 | 55 | 9.00E-09 |
| LRRC15A | leucine rich repeat containing 16A | 473 | 45 | 2.00E-12 |
| LRRC17 | leucine rich repeat containing 17 | 1436 | 62 | 6.00E-59 |
| LRRC20 | leucine rich repeat containing 20 | 1088 | 37 | 3.00E-21 |
| LRRC28 | leucine rich repeat containing 28 isoform 1 | 903 | 78 | 5.00E-19 |
| LRRC28 | leucine rich repeat containing 28 | 1026 | 88 | 2.00E-14 |
| LRRC47 | leucine rich repeat containing 47 | 2294 | 35 | 1.00E-72 |
| LRRC58 | leucine rich repeat containing 58 | 472 | 33 | 4.00E-06 |
| LRRC61 | leucine rich repeat containing 61 | 1372 | 46 | 8.00E-55 |
| Pglyrp5 | PGRP-SC1a protein | 376 | 61 | 2.00E-37 |
| FGBb | fibrinogen, B beta polypeptide | 119 | 78 | 1.00E-15 |
| FGl2 | fibrinogen-like 2 | 208 | 71 | 7.00E-24 |
| C1QBP | complement component 1, q subcomponent binding protein | 1594 | 73 | 2.00E-70 |
| C3b | complement component c3b | 5585 | 35 | 1.00E-12 |
| C6 | complement component 6 | 3928 | 48 | 0 |
| CFB | complement factor B | 2292 | 34 | 3.00E-12 |
|  | complement factor B/C2-B | 136 | 100 | 4.00E-19 |
| masp2 | mannan-binding lectin serine peptidase 2 | 476 | 36 | 2.00E-09 |
| ifnphi1 | interferon 1 | 669 | 35 | 3.00E-07 |
|  | TPA: type I interferon 2 | 460 | 83 | 9.00E-09 |
| irf1 | interferon regulatory factor 1 | 1179 | 69 | 4.00E-22 |
|  | interferon regulatory factor 2 | 1925 | 58 | 8.00E-16 |
| irf2a | interferon regulatory factor 2a | 197 | 85 | 1.00E-36 |
|  | interferon regulatory factor 2b | 155 | 84 | 2.00E-17 |
|  | interferon regulatory factor 3 | 790 | 66 | 1.00E-21 |
|  | interferon regulatory factor 4 | 2679 | 58 | 2.00E-12 |
| irf5 | interferon regulatory factor 5 | 1403 | 38 | 3.00E-42 |
| irf6 | interferon regulatory factor 6 | 203 | 63 | 5.00E-10 |
| irf7 | interferon regulatory factor 7 | 2403 | 46 | 5.00E-10 |
| irf8 | interferon regulatory factor 8 | 1663 | 90 | 5.00E-62 |
|  | Interferon regulatory factor 9 | 116 | 81 | 1.00E-10 |
| irf10 | interferon regulatory factor 10 | 1374 | 51 | 2.00E-09 |
| ifi30 | interferon gamma inducible protein 30 | 1308 | 42 | 5.00E-12 |
|  | interferon-induced protein 44-like | 488 | 65 | 6.00E-10 |
|  | interferon-inducible protein 56 | 641 | 49 | 6.00E-09 |
|  | interferon-inducible protein Gig1 | 2196 | 66 | 7.00E-38 |
|  | Interferon-induced very large GTPase 1 | 144 | 55 | 5.00E-07 |
|  | IRF-2-binding protein 2-B | 3177 | 57 | 1.00E-123 |
| irf2bp2 | IRF-2-binding protein 2-A | 2902 | 88 | 1.00E-104 |
|  | IRF-2-binding protein 1 | 2699 | 62 | 1.00E-149 |
| mxa | Interferon-induced GTP-binding protein MxA | 1210 | 56 | 1.00E-95 |
| mxb | Interferon-induced GTP-binding protein MxB | 1874 | 46 | 2.00E-29 |
| mxe | Interferon-induced GTP-binding protein MxE | 2659 | 61 | 6.00E-34 |
| irf9 | interferon-stimulated transcription factor 3, gamma 48kDa | 831 | 57 | 4.00E-07 |
| isg20l2 | interferon stimulated exonuclease gene 20-like 2 | 1855 | 46 | 2.00E-13 |
| ifrd1 | interferon-related developmental regulator 1 | 500 | 46 | 4.00E-43 |
| ifrd2 | interferon-related developmental regulator 2 | 3525 | 73 | 0 |
|  | novel IL-1 cytokine family member | 261 | 46 | 4.00E-11 |
| m17 | IL-6 subfamily cytokine M17 | 896 | 33 | 1.00E-13 |
| ik | IK cytokine | 1262 | 53 | 3.00E-70 |
|  | cytokine receptor CRFB1 | 1287 | 41 | 1.00E-13 |
|  | helical cytokine receptor CRFB7 | 570 | 53 | 3.00E-15 |
| crlf1b | cytokine receptor-like factor 1b | 228 | 34 | 3.00E-06 |
| crfb2 | cytokine receptor family member b2 | 1010 | 44 | 5.00E-09 |
| crfb6 | cytokine receptor family member b6 | 393 | 35 | 2.00E-06 |
|  | cytokine receptor CRFB4 | 126 | 71 | 2.00E-08 |
|  | cytokine receptor gamma chain | 184 | 48 | 1.00E-06 |
| crlf3 | Cytokine receptor-like factor 3 (Clf-3 protein) | 2473 | 69 | 0 |
|  | class I helical cytokine receptor member 3 | 435 | 53 | 4.00E-18 |
|  | class I helical cytokine receptor number 11 | 185 | 55 | 8.00E-08 |
|  | class I helical cytokine receptor member 12 | 754 | 50 | 1.00E-25 |
|  | class I helical cytokine receptor member 13 | 2173 | 52 | 2.00E-10 |
|  | class I helical cytokine receptor member 14 | 117 | 65 | 1.00E-07 |
|  | class I helical cytokine receptor number 17 | 1055 | 48 | 2.00E-09 |
|  | class I helical cytokine receptor number 22 | 957 | 60 | 4.00E-16 |
|  | class I helical cytokine receptor number 23 | 1502 | 48 | 9.00E-36 |
|  | class I helical cytokine receptor number 25 | 1303 | 56 | 2.00E-17 |
|  | class I helical cytokine receptor number 27 | 536 | 55 | 2.00E-12 |
|  | class I helical cytokine receptor number 26 | 113 | 72 | 9.00E-06 |
|  | cytokine receptor gamma chain | 142 | 52 | 1.00E-06 |
| socs1 | suppressor of cytokine signaling 1 | 1357 | 51 | 1.00E-14 |
| socs2 | suppressor of cytokine signaling 2 | 156 | 82 | 3.00E-19 |
| socs5b | suppressor of cytokine signaling 5b | 791 | 45 | 8.00E-08 |
| socs3b | suppressor of cytokine signaling 3b | 2217 | 62 | 3.00E-61 |
| socs6b | suppressor of cytokine signaling 6b | 3802 | 60 | 9.00E-13 |
| socs9 | suppressor of cytokine signaling 9 | 4526 | 63 | 3.00E-40 |
| crfb5 | cytokine receptor family member b5 | 658 | 46 | 1.00E-11 |
| crfb9 | cytokine receptor family member b9 | 1458 | 39 | 2.00E-06 |
|  | small inducible cytokine subfamily E, member 1 | 855 | 89 | 1.00E-104 |
|  | cytokine induced protein 29 kDa | 1387 | 39 | 4.00E-30 |
| dock1 | dedicator of cytokinesis 1 | 1752 | 62 | 3.00E-79 |
| dock4b | dedicator of cytokinesis 4b | 6343 | 71 | 0 |
| dock5 | dedicator of cytokinesis 5 | 763 | 38 | 9.00E-15 |
|  | dedicator of cytokinesis 7 | 125 | 58 | 1.00E-07 |
|  | dedicator of cytokinesis 8 isoform 1 | 256 | 53 | 1.00E-14 |
|  | dedicator of cytokinesis 8 | 324 | 57 | 3.00E-12 |
|  | dedicator of cytokinesis protein 9 | 224 | 45 | 9.00E-14 |
|  | dedicator of cytokinesis 10, isoform CRA_c | 115 | 71 | 7.00E-06 |
|  | dedicator of cytokinesis 10, isoform CRA_d | 543 | 49 | 5.00E-10 |
|  | dedicator of cytokinesis 11 | 1055 | 64 | 1.00E-113 |
|  | CC chemokine | 175 | 84 | 4.00E-10 |
|  | CC chemokine | 435 | 64 | 4.00E-10 |
|  | CC chemokine | 1716 | 100 | 2.00E-13 |
|  | C-C motif chemokine 3 precursor | 857 | 50 | 2.00E-14 |
|  | chemokine (C-C motif) ligand 20-like | 850 | 38 | 1.00E-06 |
|  | chemokine CXC-like protein | 109 | 71 | 5.00E-08 |
|  | chemokine CXC-like protein | 1175 | 52 | 2.00E-09 |
| cxcl14 | chemokine (C-X-C motif) ligand 14 | 2457 | 56 | 4.00E-15 |
| cxcl12a | chemokine (C-X-C motif) ligand 12a | 122 | 67 | 5.00E-14 |
| cxcl12b | chemokine (C-X-C motif) ligand 12b | 3853 | 66 | 2.00E-15 |
| cmklr1 | chemokine-like receptor 1 | 852 | 47 | 2.00E-21 |
| cxcr7b | chemokine (C-X-C motif) receptor 7b | 197 | 32 | 1.00E-07 |
| cxcr3.1 | chemokine (C-X-C motif) receptor 3.1 | 145 | 39 | 2.00E-07 |
| cxcr3.2 | chemokine (C-X-C motif) receptor 3.2 | 2147 | 45 | 1.00E-24 |
| cxcr4a | chemokine (C-X-C motif) receptor 4a | 1248 | 31 | 1.00E-48 |
| cxcr4b | chemokine (C-X-C motif), receptor 4b | 721 | 44 | 6.00E-12 |
| ccr6a | chemokine (C-C motif) receptor 6a | 1530 | 31 | 7.00E-28 |
| ccr7 | chemokine (C-C motif) receptor 7 | 3460 | 31 | 9.00E-07 |
| ccr12.3 | C-C chemokine receptor family-like | 479 | 47 | 3.00E-06 |
|  | CC Chemokine LARC specific receptor | 165 | 46 | 2.00E-06 |
| egfr | epidermal growth factor receptor | 6416 | 46 | 0 |
| tnfb | TNF superfamily, member 2 | 179 | 48 | 6.00E-07 |
|  | tumor necrosis factor receptor superfamily, member 9 | 1513 | 43 | 1.00E-08 |
| tnfrsf21 | tumor necrosis factor receptor superfamily, member 21 | 3440 | 43 | 3.00E-36 |
|  | tumor necrosis factor (ligand) superfamily, member 12 | 1049 | 47 | 6.00E-22 |
|  | tumor necrosis factor superfamily, member 13b | 450 | 42 | 8.00E-07 |
|  | Tumor necrosis factor ligand superfamily member 6 | 1208 | 59 | 8.00E-10 |
|  | tumor necrosis factor alpha | 224 | 76 | 2.00E-08 |
|  | tumor necrosis factor beta | 1605 | 60 | 8.00E-06 |
|  | tumor necrosis factor ligand superfamily member 13B | 155 | 62 | 1.00E-09 |
| tnfrsfa | tumor necrosis factor receptor superfamily, member a | 249 | 37 | 7.00E-09 |
| tnfrsf1a | tumor necrosis factor receptor superfamily, member 1a | 1069 | 39 | 1.00E-10 |
|  | Tumor necrosis factor receptor superfamily member 11B precursor | 943 | 40 | 2.00E-08 |
|  | tumor necrosis factor receptor-2 | 249 | 58 | 3.00E-17 |
|  | tumor necrosis factor receptor superfamily, member 11b | 472 | 51 | 5.00E-07 |
|  | Tumor necrosis factor receptor superfamily member 14 precursor | 103 | 66 | 1.00E-06 |
| relt | RELT tumor necrosis factor receptor | 819 | 58 | 4.00E-18 |
|  | tumor necrosis factor, alpha-induced protein 2 | 1621 | 33 | 1.00E-12 |
| tnfaip6 | tumor necrosis factor, alpha-induced protein 6 | 1538 | 43 | 1.00E-18 |
|  | Tumor necrosis factor, alpha-induced protein 8-like protein 2 B | 843 | 34 | 1.00E-06 |
| tnfaip1 | tumor necrosis factor, alpha-induced protein 1 | 883 | 47 | 9.00E-37 |
| tnfaip8 | Tumor necrosis factor, alpha-induced protein 8-like protein 1 | 4633 | 53 | 5.00E-56 |
|  | Tumor necrosis factor superfamily member 5-induced protein 1 | 1309 | 47 | 2.00E-25 |
|  | Tumor necrosis factor, alpha-induced protein 2 | 168 | 50 | 1.00E-07 |
|  | tumor necrosis factor, alpha-induced protein 3 | 118 | 57 | 5.00E-06 |
| cflar | CASP8 and FADD-like apoptosis regulator | 7210 | 30 | 4.00E-07 |
|  | EGF-like-domain, multiple 4, isoform CRA_b | 492 | 67 | 7.00E-17 |
| egfl6 | EGF-like-domain, multiple 6 | 186 | 45 | 1.00E-07 |
| egfr | epidermal growth factor receptor | 6416 | 46 | 0 |
| fgf13l | fibroblast growth factor 13, like | 189 | 34 | 2.00E-07 |
|  | fibroblast growth factor 1 (acidic) | 327 | 47 | 3.00E-21 |
| fgf3 | Fibroblast growth factor 3 Precursor (FGF-3) | 1488 | 42 | 8.00E-07 |
| flt1 | fms-related tyrosine kinase 1 | 1231 | 68 | 1.00E-116 |
|  | GATA zinc finger domain containing 2B-like | 1456 | 60 | 1.00E-73 |
| gatad1 | GATA zinc finger domain-containing protein 1 | 1281 | 84 | 1.00E-143 |
| gdf11 | growth differentiation factor 11 | 1291 | 91 | 1.00E-40 |
| gfi1.1 | growth factor independent 1.1 | 2139 | 85 | 1.00E-128 |
| gfi1.2 | growth factor independent 1.2 | 1223 | 69 | 1.00E-32 |
| inhbb | inhibin, beta B precursor | 5869 | 61 | 1.00E-123 |
|  | integrin, alpha 3 | 433 | 46 | 9.00E-06 |
|  | similar to integrin, alpha D | 482 | 46 | 6.00E-12 |
| itga5 | integrin alpha 5 | 101 | 69 | 9.00E-11 |
| itga6b | integrin, alpha 6b | 1345 | 44 | 7.00E-06 |
| itgav | integrin, alpha V | 142 | 44 | 4.00E-08 |
|  | integrin alpha X precursor | 406 | 41 | 1.00E-20 |
|  | alpha M integrin | 4571 | 61 | 1.00E-08 |
| itgbl1 | integrin, beta-like 1 | 1094 | 33 | 3.00E-06 |
| itgb4 | integrin, beta 4 | 748 | 41 | 3.00E-17 |
|  | integrin, beta 8 | 700 | 57 | 2.00E-07 |
| lifra | leukemia inhibitory factor receptor alpha | 5771 | 40 | 1.00E-71 |
| lifrb | leukemia inhibitory factor receptor alpha b | 154 | 51 | 3.00E-06 |
| mafba | Transcription factor MafB (Maf-B) | 1449 | 51 | 3.00E-28 |
| mif | macrophage migration inhibitory factor | 644 | 86 | 2.00E-65 |
| pcgf1 | Polycomb group RING finger protein 1 | 152 | 58 | 7.00E-15 |
| pcgf6 | polycomb group ring finger 6 | 967 | 78 | 3.00E-10 |
| sdf2l1 | stromal cell-derived factor 2-like 1 | 1513 | 42 | 2.00E-49 |
| sdf2 | stromal cell-derived factor 2 | 1724 | 74 | 2.00E-94 |
| sdf4 | Stromal cell-derived factor 4 | 2391 | 91 | 0 |
| tgfb1 | transforming growth factor, beta 1 | 479 | 77 | 1.00E-37 |
| tgfb2 | transforming growth factor, beta 2 | 135 | 82 | 2.00E-12 |
|  | vascular endothelial growth factor Aa (vegfaa), transcript variant 2 | 1878 | 64 | 2.00E-61 |
| vegfab | vascular endothelial growth factor Ab | 2569 | 54 | 1.00E-07 |
| vegfc | vascular endothelial growth factor c, mRNA | 1252 | 48 | 2.00E-16 |
| kdrl | Vascular endothelial growth factor receptor kdr-like Precursor | 3479 | 43 | 0 |
| flt4 | Vascular endothelial growth factor receptor 3 Precursor | 2438 | 50 | 1.00E-163 |
| kdr | Vascular endothelial growth factor receptor 2 Precursor | 5316 | 67 | 0 |
|  | death effector domain-containing protein | 3063 | 43 | 6.00E-40 |
| dedd1 | death effector domain-containing 1 | 2348 | 78 | 6.00E-12 |
| eif2ak2 | eukaryotic translation initiation factor 2-alpha kinase 2 | 2315 | 48 | 9.00E-08 |
| hmox1 | heme oxygenase (decyclizing) 1 | 846 | 65 | 4.00E-08 |
|  | heme oxygenase (decyclizing) 2 | 3377 | 71 | 1.00E-132 |
| irak4 | interleukin-1 receptor-associated kinase 4 | 599 | 58 | 1.00E-25 |
|  | granulocyte colony stimulating factor 1 | 175 | 62 | 3.00E-08 |
|  | granulocyte colony stimulating factor 2 | 525 | 59 | 3.00E-09 |
| csf3 | colony stimulating factor 3 (granulocyte) | 1806 | 45 | 2.00E-10 |
| csf1r | Macrophage colony-stimulating factor 1 receptor Precursor | 4778 | 44 | 1.00E-134 |
| csf2rb | colony stimulating factor 2 receptor, beta, low-affinity | 1633 | 37 | 1.00E-45 |
|  | colony stimulating factor 3 receptor (granulocyte) | 3717 | 38 | 1.00E-39 |
| myd88 | myeloid differentiation primary response gene 88 | 284 | 51 | 1.00E-07 |
|  | MyD88 adaptor-like protein | 3150 | 62 | 1.00E-11 |
| nr1d1 | nuclear receptor subfamily 1, group D, member 1 | 4080 | 50 | 3.00E-61 |
| nr1d2a | nuclear receptor subfamily 1, group D, member 2a | 2456 | 70 | 3.00E-81 |
| nr1d2b | nuclear receptor subfamily 1, group D, member 2b | 2193 | 70 | 1.00E-156 |
| nr1h4 | nuclear receptor subfamily 1, group H, member 4 | 1712 | 41 | 3.00E-18 |
| nr1h3 | nuclear receptor subfamily 1, group H, member 3 | 1561 | 45 | 7.00E-36 |
| nr2c1 | nuclear receptor subfamily 2, group C, member 1 | 2496 | 81 | 0 |
| nr2c2 | nuclear receptor subfamily 2, group C, member 2 | 1230 | 74 | 4.00E-08 |
| nr2f1a | Nuclear receptor subfamily 2 group F member 1-A | 541 | 100 | 1.00E-88 |
| nr2f1b | Nuclear receptor subfamily 2 group F member 1-B | 490 | 91 | 1.00E-15 |
| nr2f2 | nuclear receptor subfamily 2, group F, member 2 | 453 | 80 | 2.00E-18 |
| nr3c1 | nuclear receptor subfamily 3, group C, member 1 | 7580 | 79 | 1.00E-174 |
| nr3c2 | nuclear receptor subfamily 3, group C, member 2 | 510 | 75 | 1.00E-17 |
| nr4a2a | nuclear receptor subfamily 4, group A, member 2a | 892 | 66 | 8.00E-79 |
| nr4a1 | nuclear receptor subfamily 4, group A, member 1 | 1071 | 44 | 5.00E-31 |
| nr4a2b | nuclear receptor subfamily 4, group A, member 2b | 486 | 44 | 3.00E-07 |
| nr4a3 | nuclear receptor subfamily 4, group A, member 3 | 1987 | 77 | 1.00E-179 |
| nr5a1b | nuclear receptor subfamily 5, group A, member 1b | 6072 | 68 | 0 |
|  | pellino protein | 4005 | 40 | 1.00E-48 |
| peli2 | pellino homolog 2 | 1163 | 94 | 0 |
| plekha1 | pleckstrin homology domain containing, family A member 1 | 1358 | 72 | 1.00E-105 |
|  | pleckstrin homology domain containing, family A member 2 | 2145 | 53 | 7.00E-72 |
|  | pleckstrin homology domain containing, family A member 3 | 2367 | 62 | 2.00E-33 |
|  | pleckstrin homology domain containing, family A member 7 | 162 | 52 | 2.00E-07 |
| plekha8 | Pleckstrin homology domain-containing family A member 8 | 2219 | 81 | 1.00E-81 |
|  | peroxisome proliferator-activated receptor beta | 303 | 63 | 4.00E-12 |
| pparg | peroxisome proliferator activated receptor gamma | 2131 | 40 | 8.00E-31 |
| ppardb | peroxisome proliferator-activated receptor delta b | 3181 | 53 | 5.00E-89 |
| rhoaa | ras homolog gene family, member A | 3625 | 87 | 1.00E-120 |
| rhoac | ras homolog gene family, member Ac | 409 | 46 | 2.00E-28 |
| rhoad | ras homolog gene family, member Ad | 219 | 64 | 6.00E-18 |
| rhoae | ras homolog gene family, member Ae | 121 | 52 | 4.00E-06 |
| rhogb | ras homolog gene family, member G | 2141 | 47 | 4.00E-31 |
| rhoga | ras homolog gene family, member Ga | 116 | 55 | 7.00E-09 |
| rhoq | ras homolog gene family, member Q | 3354 | 69 | 5.00E-39 |
| kcnh5a | ras homolog gene family, member J | 4522 | 55 | 2.00E-28 |
| rhot1b | ras homolog gene family, member T1b | 2081 | 85 | 0 |
| rhoua | ras homolog gene family, member Ua | 2493 | 48 | 2.00E-46 |
| rhov | ras homolog gene family, member V | 2523 | 74 | 3.00E-96 |
| smad2 | Mothers against decapentaplegic homolog 2 | 1371 | 47 | 3.00E-52 |
| smad3b | mothers against decapentaplegic homolog 3b | 445 | 82 | 1.00E-76 |
| smad5 | Mothers against decapentaplegic homolog 5 | 2478 | 49 | 2.00E-59 |
| smad7 | mothers against decapentaplegic homolog 7 | 2811 | 52 | 1.00E-115 |
| smad9 | mothers against decapentaplegic homolog 8 | 2153 | 42 | 9.00E-29 |
| tradd | Tumor necrosis factor receptor type 1-associated DEATH domain protein | 1278 | 35 | 2.00E-18 |
| trap1 | TNF receptor-associated protein 1 | 4085 | 90 | 0 |
| traf3 | TNF receptor-associated factor 3 | 1243 | 67 | 5.00E-32 |
|  | TNF receptor-associated factor 5 | 462 | 55 | 6.00E-08 |
| traf6 | TNF receptor-associated factor 6 | 772 | 68 | 1.00E-103 |
| traf7 | TNF receptor-associated factor 7 | 4471 | 99 | 0 |
| ube2l3 | ubiquitin-conjugating enzyme E2L 3 | 2183 | 81 | 2.00E-78 |
|  | Ubiquitin-conjugating enzyme E2 Z | 5680 | 56 | 4.00E-22 |
| ube2r2 | ubiquitin-conjugating enzyme E2R 2 | 958 | 62 | 2.00E-07 |
|  | ubiquitin-conjugating enzyme E2E 2 | 1061 | 57 | 4.00E-41 |
|  | Ubiquitin-conjugating enzyme E2 variant 3 | 3836 | 62 | 9.00E-17 |
| ube2k | ubiquitin-conjugating enzyme E2K | 953 | 90 | 1.00E-110 |
|  | ubiquitin-conjugating enzyme E2G 2 | 688 | 92 | 1.00E-106 |
| ube2q1 | ubiquitin-conjugating enzyme E2Q (putative) 1 | 4076 | 53 | 1.00E-18 |
|  | ubiquitin-conjugating enzyme E2S | 450 | 43 | 1.00E-13 |
|  | ubiquitin-conjugating enzyme E2 variant 1 | 1189 | 53 | 1.00E-48 |
|  | ubiquitin-conjugating enzyme E2C | 719 | 85 | 6.00E-89 |
|  | ubiquitin-conjugating enzyme E2W | 3209 | 57 | 5.00E-45 |
|  | ubiquitin-conjugating enzyme E2T | 298 | 39 | 5.00E-19 |
| ube2l3l | ubiquitin-conjugating enzyme E2L 3, like | 100 | 54 | 6.00E-07 |
|  | ubiquitin-conjugating enzyme E2 D4 | 358 | 43 | 2.00E-20 |
| ube2d1 | ubiquitin-conjugating enzyme E2D 1 | 3460 | 46 | 5.00E-34 |
| ube2d2 | ubiquitin-conjugating enzyme E2D 3 | 2570 | 56 | 2.00E-39 |
| ube2a | ubiquitin-conjugating enzyme E2A | 2336 | 89 | 1.00E-58 |
| ube2h | ubiquitin-conjugating enzyme E2H | 1635 | 53 | 3.00E-35 |
| ube2q2 | ubiquitin-conjugating enzyme E2Q 2 | 666 | 57 | 2.00E-35 |
| ube2n | ubiquitin-conjugating enzyme E2N | 1754 | 64 | 2.00E-63 |
| ube2v2 | Ubiquitin-conjugating enzyme E2 variant 2 | 621 | 69 | 2.00E-72 |
| ube2g1 | ubiquitin-conjugating enzyme E2G 1 | 3310 | 44 | 5.00E-43 |
|  | ubiquitin-conjugating enzyme E2, J1 | 1937 | 60 | 1.00E-14 |
|  | Ubiquitin-conjugating enzyme E2 O | 806 | 75 | 8.00E-06 |
|  | Ubiquitin conjugation factor E4 A isoform 7 | 240 | 80 | 5.00E-06 |
| stat1a | signal transduction and activation of transcription 1a | 492 | 55 | 2.00E-12 |
| stat1b | signal transducer and activator of transcription 1b | 801 | 44 | 2.00E-18 |
| stat3 | signal transduction and activation of transcription 3 | 4516 | 84 | 2.00E-21 |
| stat4 | signal transducer and activator of transcription 4 | 2661 | 47 | 5.00E-23 |
| stat5.1 | signal transducer and activator of transcription 5.1 | 6816 | 44 | 1.00E-111 |
| stat5.2 | signal transducer and activator of transcription 5.2 | 303 | 47 | 8.00E-18 |
| stat6 | signal transducer and activator of transcription 6 | 3456 | 73 | 1.00E-27 |
| nfkb2 | nuclear factor of kappa light polypeptide gene enhancer in B-cells 2, p49/p100 | 1692 | 83 | 2.00E-29 |
|  | nuclear factor of kappa light polypeptide gene enhancer in B-cells inhibitor, epsilon | 975 | 71 | 7.00E-24 |
|  | Nuclear factor of kappa light polypeptide gene enhancer in B-cells inhibitor-like 2 | 503 | 65 | 1.00E-25 |
| nfkbiaa | nuclear factor of kappa light polypeptide gene enhancer in B-cells inhibitor, alpha a | 336 | 70 | 2.00E-50 |
| nfkbiab | nuclear factor of kappa light polypeptide gene enhancer in B-cells inhibitor, alpha b | 1585 | 67 | 7.00E-06 |
|  | nuclear factor kappa B | 168 | 53 | 3.00E-07 |
|  | nuclear factor kappa-B, subunit 1 isoform 9 | 159 | 57 | 6.00E-08 |
| mapk1 | mitogen-activated protein kinase 1 | 2224 | 56 | 5.00E-06 |
|  | mitogen-activated protein kinase 11 | 4059 | 41 | 1.00E-39 |
| mapk3 | mitogen-activated protein kinase 3 | 662 | 74 | 1.00E-115 |
| mapk6 | mitogen-activated protein kinase 6 | 336 | 49 | 3.00E-28 |
| mapk7 | mitogen-activated protein kinase 7 | 4153 | 32 | 9.00E-07 |
|  | mitogen-activated protein kinase 8 | 1399 | 54 | 1.00E-118 |
| mapk10 | mitogen-activated protein kinase 10 | 4256 | 62 | 1.00E-125 |
|  | mitogen-activated protein kinase 12 | 1690 | 40 | 3.00E-53 |
| mapk14 | Mitogen-activated protein kinase 14a | 1767 | 34 | 1.00E-15 |
|  | mitogen-activated protein kinase 15 | 1116 | 37 | 1.00E-15 |
|  | Mitogen-activated protein kinase organizer 1 | 481 | 86 | 1.00E-12 |
|  | mitogen-activated protein kinase kinase 2 | 2005 | 48 | 1.00E-98 |
| map2k6 | Dual specificity mitogen-activated protein kinase kinase 6 | 3431 | 39 | 6.00E-74 |
| map2k4 | mitogen-activated protein kinase kinase 4 | 5981 | 52 | 2.00E-47 |
| map4k2l | mitogen-activated protein kinase kinase kinase kinase 2-like | 2983 | 34 | 1.00E-06 |
| map3k7 | mitogen-activated protein kinase kinase kinase 7 | 3919 | 44 | 5.00E-90 |
|  | Mitogen-activated protein kinase kinase kinase 8 | 2261 | 55 | 5.00E-34 |
|  | mitogen-activated protein kinase kinase kinase kinase 2 | 2734 | 82 | 0 |
| map3k5 | mitogen-activated protein kinase kinase kinase 5 | 3845 | 92 | 0 |
| map3k12 | mitogen-activated protein kinase kinase kinase 12 | 3705 | 37 | 2.00E-06 |
|  | Mitogen-activated protein kinase kinase kinase 14 | 164 | 66 | 2.00E-13 |
|  | mitogen-activated protein kinase kinase kinase 4 | 2218 | 68 | 1.00E-32 |
|  | mitogen-activated protein kinase associated protein 1 isoform 3 isoform 5 | 2913 | 72 | 0 |
|  | Mitogen-activated protein kinase kinase kinase 11 | 107 | 91 | 9.00E-09 |
|  | mitogen-activated protein kinase kinase kinase kinase 3 | 120 | 76 | 1.00E-09 |
|  | mitogen-activated protein kinase kinase kinase kinase 4-like protein | 538 | 52 | 2.00E-08 |
| mapkapk2a | mitogen-activated protein kinase-activated protein kinase 2a | 2794 | 43 | 2.00E-28 |
| mapkapk2b | mitogen-activated protein kinase-activated protein kinase 2b | 602 | 66 | 2.00E-14 |
| mapkapk3 | mitogen-activated protein kinase-activated protein kinase 3 | 706 | 37 | 9.00E-08 |
|  | mitogen-activated protein kinase 8 interacting protein 3 | 338 | 83 | 6.00E-52 |
| MAPKBP1 | vertebrate mouse mitogen-activated protein kinase binding protein 1-like | 845 | 42 | 6.00E-07 |
|  | Mitogen-activated protein-binding protein-interacting protein | 416 | 42 | 2.00E-25 |
| map3k7ip3l | mitogen-activated protein kinase kinase kinase 7 interacting protein 3 | 1226 | 71 | 6.00E-10 |
| junb | jun B proto-oncogene | 201 | 51 | 2.00E-10 |
| jun | c-Jun protein | 916 | 80 | 8.00E-89 |
| jund | jun D proto-oncogene | 2030 | 93 | 1.00E-106 |
|  | T cell receptor alpha | 4194 | 68 | 1.00E-10 |
|  | T-cell receptor alpha chain V region HPB-MLT precursor | 4788 | 47 | 3.00E-08 |
|  | T-cell receptor beta chai | 484 | 68 | 3.00E-08 |
|  | T cell receptor beta chain constant region | 1158 | 68 | 4.00E-11 |
|  | T-cell receptor beta chain precursor | 489 | 42 | 9.00E-22 |
|  | T cell receptor beta chain VDJC | 493 | 80 | 3.00E-07 |
|  | T cell receptor V alpha chain | 147 | 57 | 2.00E-06 |
|  | T-cell receptor V-alpha6 chain precursor | 167 | 51 | 5.00E-07 |
|  | TCR-gamma constant region 4 | 468 | 50 | 1.00E-15 |
| bcl2 | bcl2 | 1472 | 45 | 2.00E-09 |
| bcl2l13 | BCL2-like 13 | 6225 | 57 | 1.00E-104 |
| bcl6 | B-cell lymphoma 6 protein | 1665 | 64 | 3.00E-16 |
| bcl7b | B-cell CLL/lymphoma 7b | 177 | 85 | 7.00E-21 |
| bcl9l | B-cell CLL/lymphoma 9-like protein | 2198 | 61 | 2.00E-50 |
| bcl11a | B-cell CLL/lymphoma 11A | 4685 | 54 | 1.00E-25 |
|  | immunoglobulin superfamily, member 3 | 501 | 60 | 1.00E-08 |
|  | immunoglobulin D | 211 | 55 | 2.00E-14 |
|  | immunoglobulin delta heavy chain | 3356 | 55 | 1.00E-14 |
|  | immunoglobulin mu heavy chain precursor | 1382 | 61 | 2.00E-09 |
|  | immunoglobulin heavy chain IgH | 362 | 42 | 7.00E-09 |
|  | immunoglobulin heavy chain | 1994 | 63 | 0 |
|  | immunoglobulin heavy chain variable region | 166 | 60 | 4.00E-09 |
|  | immunoglobulin light chain variable region | 433 | 54 | 6.00E-19 |
|  | immunoglobulin light chain type 1 | 1336 | 73 | 3.00E-06 |
|  | Immunoglobulin superfamily member 6 precursor | 327 | 43 | 2.00E-12 |
| IGBP1 | immunoglobulin binding protein 1 | 1143 | 46 | 8.00E-64 |
|  | CD3 epsilon | 1774 | 53 | 4.00E-12 |
|  | CD3 gamma/delta | 165 | 53 | 4.00E-07 |
|  | CD4-2 protein | 641 | 40 | 6.00E-34 |
|  | cd4-4 protein | 255 | 38 | 3.00E-08 |
|  | CD8 beta | 477 | 53 | 5.00E-09 |
|  | CD9 antigen | 412 | 62 | 2.00E-06 |
|  | CD11-2 | 743 | 48 | 2.00E-06 |
|  | CD18 protein | 150 | 58 | 4.00E-06 |
|  | CD18 type 2 | 197 | 40 | 4.00E-06 |
|  | CD22 molecule | 253 | 40 | 3.00E-07 |
|  | Myeloid cell surface antigen CD33 precursor | 1505 | 73 | 2.00E-17 |
|  | CD36 antigen | 124 | 51 | 4.00E-11 |
|  | CD40 antigen precursor | 720 | 40 | 3.00E-07 |
|  | CD45 | 138 | 69 | 1.00E-06 |
|  | CD48 antigen precursor | 701 | 4 | 6.00E-35 |
|  | leukocyte surface antigen CD53 | 664 | 93 | 3.00E-12 |
| cd63 | Cd63 antigen | 1192 | 51 | 1.00E-09 |
|  | CD81 antigen | 185 | 50 | 2.00E-06 |
|  | CD82 antigen | 6829 | 58 | 1.00E-26 |
|  | CD83 | 172 | 56 | 2.00E-06 |
|  | CD97 antigen precursor | 529 | 55 | 3.00E-16 |
|  | CD99 antigen-like protein 2 Precursor | 492 | 75 | 1.00E-20 |
|  | CD151 molecule | 1196 | 30 | 9.00E-06 |
|  | CD166 antigen homolog Precursor | 643 | 47 | 4.00E-25 |
|  | similar to CD200 molecule | 205 | 42 | 2.00E-07 |
|  | CD205 | 140 | 80 | 1.00E-15 |
|  | CD226 molecule | 1435 | 54 | 4.00E-07 |
|  | CD302 antigen precursor | 277 | 51 | 4.00E-25 |
|  | CD209 antigen-like protein A | 482 | 71 | 7.00E-06 |
|  | CD300 antigen-like family member | 336 | 42 | 4.00E-08 |
| blnk | B-cell linker | 2817 | 41 | 1.00E-07 |
| aicda | activation-induced cytidine deaminase | 115 | 65 | 1.00E-06 |
| dram1 | damage-regulated autophagy modulator | 1019 | 38 | 7.00E-40 |
| hdac1 | histone deacetylase 1 | 1509 | 80 | 1.00E-172 |
| hdac3 | Histone deacetylase 3 | 2554 | 92 | 0 |
| hdac4 | histone deacetylase 4 | 4656 | 80 | 1.00E-104 |
| hdac8 | histone deacetylase 8 | 929 | 78 | 4.00E-55 |
| sap30l | Histone deacetylase complex subunit SAP30L | 2999 | 67 | 7.00E-56 |
| klf2a | Kruppel-like factor 2a | 118 | 70 | 2.00E-10 |
| klf2b | Kruppel-like factor 2b | 2610 | 68 | 2.00E-64 |
| klf3 | Kruppel-like factor 3 (basic) | 3765 | 75 | 2.00E-43 |
| klfd | Kruppel-like factor d | 1394 | 46 | 5.00E-08 |
| copeb | Kruppel-like factor 6 | 1733 | 45 | 9.00E-27 |
| klf7 | Kruppel-like factor 7 | 136 | 76 | 1.00E-13 |
| klf11a | Kruppel-like factor 11a | 144 | 57 | 1.00E-08 |
| klf11b | Kruppel-like factor 11b | 1068 | 77 | 9.00E-60 |
| klf12a | Kruppel-like factor 12 | 497 | 88 | 3.00E-57 |
| klf12b | Kruppel-like factor 12b | 124 | 60 | 4.00E-10 |
| klf13l | Kruppel-like factor 13 like | 1161 | 40 | 1.00E-06 |
| jag1a | Protein jagged-1a Precursor | 2457 | 46 | 4.00E-83 |
| jag1b | Protein jagged-1b Precursor | 789 | 85 | 6.00E-14 |
| jag2 | jagged 2 isoform 1 | 4712 | 67 | 0 |
| pawrl | PRKC, apoptosis, WT1, regulator like | 109 | 50 | 2.00E-16 |
| prkcz | protein kinase C, zeta | 848 | 39 | 1.00E-17 |
| prkch | protein kinase C, eta | 591 | 68 | 6.00E-67 |
| prkcq | protein kinase C, theta | 1096 | 65 | 3.00E-30 |
| prkci | Protein kinase C iota type | 2195 | 44 | 1.00E-69 |
| prkcb1 | protein kinase C, beta | 935 | 52 | 7.00E-59 |
| prkcb1l | protein kinase C, beta 1, like | 5663 | 81 | 0 |
| prkcd | protein kinase C, delta | 3507 | 42 | 1.00E-148 |
| prlrb | prolactin receptor b | 2562 | 58 | 3.00E-14 |
| rgs20 | regulator of G-protein signaling 20 | 2911 | 68 | 2.00E-63 |
| rgs16 | regulator of G-protein signaling 16 | 1045 | 44 | 5.00E-11 |
| rgs5 | regulator of G-protein signaling 5 | 287 | 62 | 5.00E-10 |
|  | regulator of G-protein signaling 9 | 789 | 61 | 2.00E-28 |
|  | regulator of G-protein signaling 3, partial | 573 | 62 | 2.00E-35 |
| rgs11 | regulator of G-protein signalling 11 | 488 | 72 | 3.00E-06 |
| rgs19 | regulator of G-protein signalling 19 | 1159 | 39 | 6.00E-33 |
| rgs4 | regulator of G-protein signalling 4 | 1756 | 52 | 2.00E-35 |
| rgs14 | regulator of G-protein signalling 14 | 1564 | 38 | 9.00E-13 |
| rgs12 | RGS12TS-S | 474 | 97 | 2.00E-41 |
| wasla | Wiskott-Aldrich syndrome-like a | 907 | 66 | 1.00E-25 |
| wasb | Wiskott-Aldrich syndrome b | 878 | 36 | 4.00E-08 |
| mhc2a | major histocompatibility complex class II integral membrane alpha chain gene | 1934 | 43 | 5.00E-26 |
|  | major histocompatibility complex class I UDA gene | 1605 | 43 | 1.00E-57 |
|  | MHC class I antigen | 131 | 62 | 5.00E-07 |
|  | MHC class II beta antigen | 102 | 72 | 9.00E-06 |
|  | MHC class IA antigen | 257 | 70 | 9.00E-06 |
| psma2 | proteasome subunit alpha type 2 | 606 | 98 | 1.00E-132 |
| psma5 | proteasome subunit, alpha type, 5 | 332 | 52 | 5.00E-18 |
| psmb7 | proteasome subunit, beta type, 7 | 526 | 56 | 1.00E-35 |
| psmb10 | proteasome subunit, beta type, 10 | 461 | 91 | 5.00E-51 |
| lgmn | legumain | 5227 | 51 | 1.00E-31 |
|  | itchy homolog E3 ubiquitin protein ligase | 134 | 62 | 4.00E-06 |
| rfx1a | regulatory factor X1 | 1223 | 41 | 7.00E-13 |
|  | regulatory factor X domain containing 2 homolog | 1658 | 70 | 5.00E-06 |
|  | regulatory factor X, 3 | 3227 | 71 | 0 |
|  | TAP binding protein-like, isoform CRA_c | 293 | 41 | 4.00E-08 |
| ppp1r13b | apoptosis-stimulating protein of p53, 1 | 1591 | 73 | 1.00E-35 |
| rac2 | ras-related C3 botulinum toxin substrate 2 | 184 | 58 | 1.00E-11 |
| rac3 | ras-related C3 botulinum toxin substrate 3 | 2410 | 49 | 2.00E-47 |
| cdk1 | cyclin-dependent kinase 1 | 627 | 75 | 6.00E-78 |
| cdk2 | cyclin-dependent kinase 2 | 476 | 94 | 4.00E-17 |
| cdk2 | cyclin-dependent kinase 2 | 702 | 42 | 7.00E-19 |
| cdk5 | cyclin-dependent protein kinase 5 | 3492 | 99 | 0 |
| cdk7 | cyclin-dependent kinase 7 | 2271 | 43 | 5.00E-62 |
| cdk9 | cyclin-dependent kinase 9 | 1438 | 56 | 1.00E-114 |
|  | cyclin-dependent kinase 10 | 1868 | 90 | 0 |
|  | cyclin-dependent kinase inhibitor 1b, like | 1589 | 30 | 4.00E-08 |
| cdkn1c | cyclin-dependent kinase inhibitor 1C | 142 | 70 | 3.00E-19 |
| cdkn3 | cyclin-dependent kinase inhibitor 3 | 715 | 69 | 2.00E-83 |
|  | CTLA4-like protein | 1436 | 55 | 2.00E-07 |
|  | apolipoprotein A1 | 122 | 75 | 7.00E-09 |
|  | Apolipoprotein Eb Precursor (Apo-Eb) | 321 | 50 | 4.00E-17 |
| apoob | apolipoprotein O, b | 679 | 56 | 5.00E-37 |
|  | Apolipoprotein-L3 | 192 | 57 | 7.00E-11 |
| hells | helicase, lymphoid-specific | 6046 | 40 | 1.00E-11 |
|  | inosine monophosphate dehydrogenase 1 | 5727 | 50 | 1.00E-123 |
| impdh2 | inosine monophosphate dehydrogenase 2 | 2228 | 82 | 0 |
| fosl1 | FOS-like antigen 1 | 1621 | 35 | 9.00E-12 |
|  | FOS-like antigen 2 | 2812 | 83 | 2.00E-55 |
| egr1 | Early growth response protein 1 | 2056 | 63 | 4.00E-12 |
| egr2b | Early growth response protein 2b | 793 | 55 | 3.00E-24 |
|  | early growth response 3 | 1197 | 37 | 1.00E-29 |
| pea3 | ETS domain-containing transcription factor PEA3 | 2391 | 84 | 0 |
|  | ETS domain-containing protein Elk-3 | 976 | 77 | 3.00E-06 |
| ikbkb | inhibitor of nuclear factor kappa B kinase beta subunit | 3272 | 73 | 0 |
| chuk | Inhibitor of nuclear factor kappa-B kinase subunit alpha | 1847 | 45 | 2.00E-91 |
| jak1 | Tyrosine-protein kinase Jak1 | 2242 | 78 | 3.00E-60 |
| jak2a | Janus kinase 2a | 2720 | 35 | 9.00E-13 |
| jak2b | Janus kinase 2 | 4805 | 47 | 0 |
|  | Janus kinase family | 116 | 60 | 1.00E-06 |
| malt1 | mucosa associated lymphoid tissue lymphoma translocation gene 1 | 1968 | 41 | 2.00E-14 |
| nfat5 | nuclear factor of activated T-cells 5, tonicity-responsive | 8281 | 42 | 3.00E-71 |
|  | nuclear factor of activated T-cells, cytoplasmic, calcineurin-dependent 3 | 1353 | 70 | 4.00E-06 |
| nfatc1 | nuclear factor of activated T-cells, cytoplasmic, calcineurin-dependent 1 | 3078 | 48 | 3.00E-41 |
| ppm1a | protein phosphatase 1A, magnesium dependent, alpha | 1274 | 87 | 1.00E-180 |
| ppm1d | protein phosphatase 1D magnesium-dependent, delta isoform | 531 | 32 | 3.00E-12 |
| ppm1g | protein phosphatase 1G (formerly 2C), magnesium-dependent, gamma isoform | 3971 | 47 | 2.00E-65 |
| ppme1 | protein phosphatase methylesterase 1 | 1848 | 57 | 2.00E-85 |
| ppm1k | protein phosphatase 1K | 1061 | 75 | 5.00E-34 |
| dstyk | Receptor-interacting serine/threonine-protein kinase 5 | 2452 | 36 | 7.00E-07 |
|  | Receptor-interacting serine/threonine-protein kinase 4 | 1180 | 69 | 2.00E-11 |
| sp1 | sp1 transcription factor | 3140 | 75 | 1.00E-61 |
| sp4 | sp4 transcription factor | 5598 | 71 | 1.00E-141 |
| tbk1 | TANK-binding kinase 1 | 619 | 97 | 1.00E-137 |
| tbx2a | T-box gene 2a | 158 | 69 | 3.00E-09 |
| tbr1 | T-box 1, brain | 1814 | 61 | 5.00E-60 |
| tbx2b | T-box transcription factor TBX2b | 450 | 60 | 4.00E-20 |
| tbx16 | T-box gene 16 | 988 | 54 | 8.00E-40 |
| tbx21 | T-box 21 | 1730 | 79 | 1.00E-23 |
| tmed1 | transmembrane emp24 protein transport domain containing 1 | 352 | 40 | 1.00E-09 |
| tmed3 | transmembrane emp24 protein transport domain containing 3 | 455 | 61 | 7.00E-48 |
|  | transmembrane emp24 protein transport domain containing 4 | 2627 | 81 | 1.00E-116 |
| tmed5 | transmembrane emp24 protein transport domain containing 5 | 550 | 39 | 4.00E-26 |
| tmed7 | transmembrane emp24 protein transport domain containing 7 | 1782 | 53 | 3.00E-32 |
| tmed9 | transmembrane emp24 protein transport domain containing 9 | 137 | 95 | 8.00E-25 |
| tmed10 | transmembrane emp24 domain-containing protein 10 | 502 | 69 | 2.00E-21 |
|  | Transmembrane emp24 domaincontaining | 959 | 71 | 1.00E-12 |
|  | tripartite motif-containing 2 | 4433 | 45 | 4.00E-18 |
| trim13 | tripartite motif-containing 13 | 5769 | 33 | 2.00E-47 |
| trim3b | tripartite motif-containing 3b | 2415 | 43 | 1.00E-109 |
|  | Tripartite motif-containing protein 16 | 364 | 52 | 3.00E-06 |
| trim24 | tripartite motif-containing 24 | 2345 | 35 | 7.00E-16 |
| trim25 | tripartite motif-containing 25 | 934 | 41 | 1.00E-35 |
| trim32 | tripartite motif-containing 32 | 214 | 54 | 6.00E-06 |
| trim35 | tripartite motif-containing 35 | 1905 | 36 | 2.00E-38 |
|  | tripartite motif-containing 41 | 3872 | 78 | 0 |
| cdc37l1 | Hsp90 co-chaperone Cdc37-like 1 | 477 | 48 | 1.00E-33 |
|  | J-type co-chaperone HSC20 | 947 | 54 | 4.00E-54 |
|  | ferritin heavy chain | 992 | 76 | 2.00E-14 |
| hspa4 | heat shock protein 4 | 591 | 38 | 2.00E-24 |
| hspa9 | heat shock protein 9 | 3009 | 73 | 0 |
| hspa12b | heat shock protein 12B | 1275 | 68 | 1.00E-16 |
| hspa14 | heat shock protein 14 | 3579 | 41 | 2.00E-06 |
|  | Heat shock protein 67B2 | 328 | 40 | 2.00E-14 |
| hsp90a.1 | Heat shock protein HSP 90-alpha 1 | 541 | 90 | 4.00E-38 |
| hsp90a.2 | heat shock protein 90-alpha 2 | 896 | 56 | 2.00E-42 |
| hsp90ab1 | Heat shock protein HSP 90-beta | 116 | 76 | 2.00E-11 |
| hsp90b1 | heat shock protein 90kDa beta, member 1 | 462 | 55 | 4.00E-29 |
| a2ml | alpha-2 macroglobulin-like | 6423 | 56 | 0 |
| mapre1 | microtubule-associated protein, RP/EB family, member 1 | 1009 | 40 | 6.00E-30 |
|  | microtubule-associated protein, RP/EB family, member 2 | 1944 | 52 | 1.00E-28 |
| map1lc3a | microtubule-associated protein 1 light chain 3 alpha | 282 | 89 | 2.00E-52 |
|  | microtubule-associated protein 1-light chain 3C | 106 | 65 | 1.00E-08 |
| plp2 | proteolipid protein 2 | 2591 | 53 | 1.00E-33 |
|  | 6.8 kDa mitochondrial proteolipid-like | 299 | 59 | 1.00E-13 |
| phb | prohibitin | 796 | 79 | 1.00E-115 |
| phb2 | prohibitin 2 | 514 | 44 | 3.00E-28 |
| sepn1 | Selenoprotein N (SelN)(SePN) | 748 | 60 | 2.00E-14 |
|  | Selenoprotein K (SelK) | 306 | 56 | 8.00E-20 |
|  | Selenoprotein O (SelO) | 2770 | 42 | 1.00E-120 |
|  | Selenoprotein S (SelS) | 213 | 53 | 3.00E-14 |
| selt2 | Selenoprotein T2 Precursor | 994 | 69 | 3.00E-84 |
| sepm | Selenoprotein M Precursor (SelM) | 442 | 67 | 7.00E-24 |
| selt1a | Selenoprotein T1a Precursor | 435 | 92 | 1.00E-86 |
|  | 15 kDa selenoprotein Precursor | 431 | 74 | 1.00E-60 |
| sepw2a | selenoprotein W, 2a | 427 | 75 | 1.00E-35 |
|  | selenoprotein P, plasma, 1a (sepp1a) | 135 | 55 | 1.00E-13 |
|  | selenoprotein I | 1165 | 64 | 1.00E-21 |
| CAMP1 | Calmodulin-regulated spectrin-associated protein 1 | 517 | 50 | 1.00E-13 |
|  | caspase recruitment domain protein 9 | 4023 | 35 | 5.00E-42 |
|  | caspase recruitment domain family, member 15 | 397 | 68 | 3.00E-10 |
|  | caspase recruitment domain family, member 6 | 178 | 54 | 5.00E-06 |
|  | caspase recruitment domain family, member 11 | 303 | 55 | 1.00E-19 |
|  | caspase recruitment domain 15 protein | 997 | 40 | 1.00E-06 |
| cebp1 | CCAAT/enhancer binding protein (C/EBP) 1 | 2941 | 64 | 5.00E-29 |
|  | CCAAT/enhancer binding protein zeta | 520 | 67 | 1.00E-14 |
| cebpb | CCAAT/enhancer binding protein beta | 2366 | 62 | 6.00E-07 |
| cebpg | CCAAT/enhancer binding protein gamma | 1128 | 47 | 1.00E-14 |
| cebpd | CCAAT/enhancer binding protein (C/EBP), delta | 515 | 76 | 2.00E-44 |
| Dmbp1 | deleted in malignant brain tumors 1 | 467 | 52 | 9.00E-22 |
| s100a1 | S100 calcium binding protein A1 | 327 | 47 | 2.00E-09 |
| s100a10a | S100 calcium binding protein A10a | 184 | 82 | 8.00E-15 |
| s100v2 | S100 calcium binding protein V2 | 1304 | 59 | 5.00E-25 |
| s100v2 | S100 calcium binding protein V2 | 656 | 48 | 2.00E-09 |
| s100u | S100 calcium binding protein U | 415 | 50 | 2.00E-23 |
|  | Caspase-1 precursor | 2000 | 40 | 3.00E-14 |
| casp2 | caspase 2, apoptosis-related cysteine protease | 2304 | 72 | 1.00E-147 |
| casp3a | caspase 3, apoptosis-related cysteine protease a | 2523 | 79 | 1.00E-123 |
| casp3b | caspase 3, apoptosis-related cysteine protease b | 151 | 43 | 2.00E-06 |
| casp6 | caspase 6 | 524 | 63 | 9.00E-65 |
|  | caspase 7, apoptosis-related cysteine peptidase | 3157 | 55 | 5.00E-51 |
| casp8 | caspase 8, apoptosis-related cysteine peptidase | 900 | 53 | 6.00E-12 |
|  | caspase 8 short isoform | 1031 | 47 | 5.00E-06 |
| casp9 | caspase 9, apoptosis-related cysteine protease | 1512 | 79 | 1.00E-177 |
| caspb | caspase b | 1614 | 41 | 2.00E-15 |
| caspc | caspase c | 786 | 32 | 1.00E-07 |
|  | caspase recruitment domain family, member 6 | 178 | 54 | 5.00E-06 |
|  | caspase recruitment domain protein 9 | 4023 | 35 | 5.00E-42 |
|  | caspase recruitment domain family, member 11 | 303 | 55 | 1.00E-19 |
|  | caspase recruitment domain 15 protein | 997 | 40 | 1.00E-06 |
|  | adenosine receptor A2a.2 | 1722 | 75 | 5.00E-85 |
|  | Fas apoptotic inhibitory molecule | 866 | 69 | 4.00E-91 |
|  | Fas apoptotic inhibitory molecule 2 | 513 | 50 | 5.00E-15 |
| apaf1 | Apoptotic protease-activating factor 1 | 377 | 51 | 6.00E-21 |
| acin1a | apoptotic chromatin condensation inducer 1a | 7315 | 64 | 1.00E-132 |
|  | apoptotic chromatin condensation inducer 1 | 674 | 82 | 6.00E-08 |
| Irg1 | novel protein similar to mouse immunoresponsive gene 1 | 1340 | 43 | 3.00E-18 |
|  | oxidative stress responsive 1 | 588 | 68 | 5.00E-07 |
| oxsr1a | oxidative-stress responsive 1a | 2715 | 52 | 9.00E-25 |
| oxsr1b | oxidative-stress responsive 1b | 1699 | 90 | 0 |
| WSCD2 | WSC domain-containing protein 2 | 4229 | 78 | 0 |
|  | oxidative stress induced growth inhibitor 1 | 3175 | 58 | 4.00E-31 |
