# Supplementary material for: De Novo Characterization of the Spleen Transcriptome of the Large Yellow Croaker (Pseudosciaena crocea) and Analysis of the Immune Relevant Genes and Pathways Involved in the Antiviral Response
Source: PLoS One. 2014 May 12;9(5):e97471. doi: 10.1371/journal.pone.0097471 (PMC4018400; doi:10.1371/journal.pone.0097471)
Supplement: Table S5 — Primers for quantitative real-time PCR. Primer set was designed based on each identified gene sequence of transcriptome library by Primer Primer 5.0. (DOC) [file pone.0097471.s006.doc]

**Table S5.** Primers for relative quantitative real-time PCR

| **Gene name** | **Forward Primer(5’----3’)** | **Reverse Primer(5’----3’)** |
| --- | --- | --- |
| β-actin | GACCTGACAGACTACCTCATG | AGTTGAAGGTGGTCTCGTGGA |
| TLR3 | CGGCCTCTCGTGCAAAGATAT | ACCGCACCAGAAGTGCAGTAAC |
| TLR22 | AGCACCGACTTCATCTGCTTTG | TGGTCTTCCTGCTCGCATAGATG |
| MDA5 | GCCAGAAGCCAAGAAAAAGC | TGTTCACCAGGACGACCACT |
| TRAF3 | GGTCCGTCAAGGAAACAC | CAAGAAGAAGAGCAGCAAG |
| TBK1 | GCAGTCTGTCCAAGGGTCT | CGTGTTGTACTCGTGGATGTAG |
| IRF3 | GTAGCAGACAGCCCATAGAGC | CCATTGAAGTCAGGACCACC |
| IRF7 | ATCTCGCACAACAGCCTCTA | TTCATTTCTATTCTTCCACT |
| IFN1 | GGACTGGACAGGAGTGTCGAG | CAGGAAGCAGAGGTGAGGTTG |
| IL-12 | TGCTCCACAGAAATCAAC | AGGCTGGACAGAACTGAT |
| MxA | GATGCTATAAGCCTCACCACA | GTTGATAAATCCTGGCAGTTC |
| viperin | TTCAGGCATGGAAAAGAT | GACGATGCTGACACTTGG |
| PRK | TCCCAAGACTTCACAAAT | TAGCGACAATGACTAAACC |
| STAT1 | ACCCGCTCCGCTTCCTCTAC | GACATCGGCATCATGTTGTC |
| STAT3 | TCTCCCAGATCCCCTTCCAT | GCCTCCAGTGTGTTCTCCTT |
| STAT6 | ACTGATGCTGACGGGAGAAC | GTGACGAGGAAGAGAGGGAA |
| TCRα | GAACTCTGCTGAATATGGAAG | CCTCATCATGCAGTGTCAAC |
| TCRβ | GGTGACAACGACTATCCCTA | GAACTCGCTGCACAGAAG |
| SPL76  SPL76  SPL76  SPL76  SPL76  SPL76  SPL76  SPL76  SPL76 | TCACCGCAACACCACATC | TGGAGAAGGACGAAACGA |
